# Supplementary material for: “Antimicrobial utilization in a paediatric intensive care unit in India: A step towards strengthening antimicrobial stewardship practices"
Source: PLoS One. 2024 Sep 19;19(9):e0310515. doi: 10.1371/journal.pone.0310515 (PMC11412675; doi:10.1371/journal.pone.0310515)
Supplement: S1 Table — Note: B = Blood, U = Urine, TBA = Tracheobronchial aspirate, P = Pus, S = Sputum, ST = Stool. * Denominator taken is total number of isolates (= 46). (DOCX) [file pone.0310515.s001.docx]

**S1 Table.** Isolated pathogens and targeted antimicrobial therapy

| Microorganisms | Sample | | No. (%) of isolates* | Targeted therapy |
| --- | --- | --- | --- | --- |
|  | | | | **Targeted therapy used against isolated organisms* n (%)** |
| Gram positive organisms | | | | |
| Methicillin-sensitive *Staphylococcus aureus (MSSA)* | | B = 1, TBA = 1 | 2 (4.3%) | 1 (2.17%) |
| Methicillin-resistant *S. aureus (MRSA)* | | B = 1, TBA = 1, P = 1, S = 1 | 4 (8.7%) | 4 (8.70%) |
| Coagulase-negative *Staphylococcus (CoNS)* | | B = 9 | 9 (19.6%) | 4 (8.70%) |
| *Enterococcus species* | | B = 1, U = 1 | 2 (4.3%) | 2 (4.35%) |
| Gram negative organisms | | | | |
| *Escherichia coli* | | B = 3, P = 4, U = 2, ST = 1 | 10 (21.7%) | 4 (8.70%) |
| *Klebsiella species* | | B = 1, TBA = 2, U = 1 | 4 (8.7%) | 1 (2.17%) |
| *Pseudomonas species* | | B = 1, TBA = 2, U = 1 | 4 (8.7%) | 1 (2.17%) |
| *Acinetobacter species* | | B = 3, TBA = 6 | 9 (19.6%) | 4 (8.70%) |
| Fungi | | | | |
| *Candida* species | |  | 2 (4.3%) | 1(2.17%) |
| *Candida tropicalis* | | U = 1 | 1 (2.2%) | 1 (2.17%) |
| *Candida krusei* | | B = 1 | 1 (2.2%) | 0 (0) |
| Total | | | 46 | 22 (47.8%) |

**Note:** B = Blood, U = Urine, TBA = Tracheobronchial aspirate, P = Pus, S = Sputum, ST = Stool. ^*^ Denominator taken is total number of isolates (=46)
