# Supplementary material for: “Antimicrobial utilization in a paediatric intensive care unit in India: A step towards strengthening antimicrobial stewardship practices"
Source: PLoS One. 2024 Sep 19;19(9):e0310515. doi: 10.1371/journal.pone.0310515 (PMC11412675; doi:10.1371/journal.pone.0310515)
Supplement: S1 Fig — Note: Category 1 = Number of patients who were prescribed 1 antimicrobial agent, Category 2 = Number of patients who were prescribed 2 antimicrobial agents, Category 3 = Number of patients who were prescribed ≥3 antimicrobial agents. (DOCX) [file pone.0310515.s002.docx]

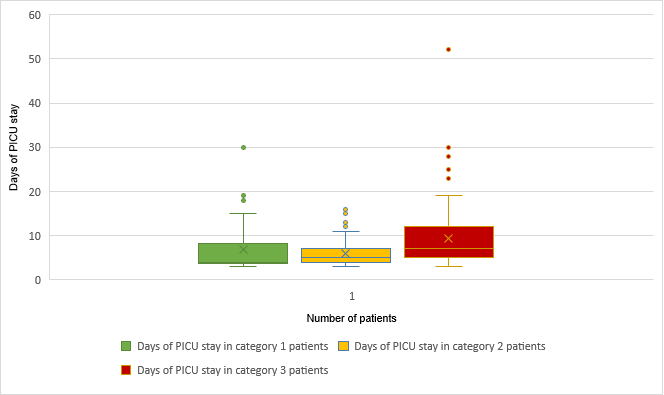


**Note:** Category 1= Number of patients who were prescribed 1 antimicrobial agent, Category 2 = Number of patients who were prescribed 2 antimicrobial agents, Category 3 = Number of patients who were prescribed ≥3 antimicrobial agents

**S1 Fig.** Box and Whisker plot for PICU stay by number of antimicrobial agents prescribed
