# Supplementary material for: “Antimicrobial utilization in a paediatric intensive care unit in India: A step towards strengthening antimicrobial stewardship practices"
Source: PLoS One. 2024 Sep 19;19(9):e0310515. doi: 10.1371/journal.pone.0310515 (PMC11412675; doi:10.1371/journal.pone.0310515)
Supplement: S2 Fig — Note: Category 1 = Antimicrobial treatment days ≤7 days, Category 2 = Antimicrobial treatment days 8–14 days, Category 3 = Antimicrobial treatment days ≥15 days. (DOCX) [file pone.0310515.s003.docx]

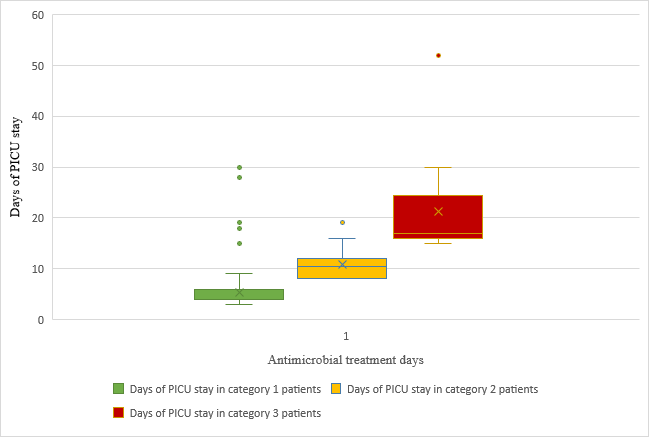


**Note:** Category 1= Antimicrobial treatment days ≤7 days, Category 2= Antimicrobial treatment days 8-14 days, Category 3= Antimicrobial treatment days ≥15 days

**S2 Fig**. Box and Whisker plot for PICU stay by antimicrobial treatment days
