# Supplementary material for: “Antimicrobial utilization in a paediatric intensive care unit in India: A step towards strengthening antimicrobial stewardship practices"
Source: PLoS One. 2024 Sep 19;19(9):e0310515. doi: 10.1371/journal.pone.0310515 (PMC11412675; doi:10.1371/journal.pone.0310515)
Supplement: S3 Fig — Note: *Denominator for ‘overall percentage’: Total number of patients under surveillance (= 216) and for ‘percentage of patients prescribed as empirical therapy’: Total number of patients who received respective antimicrobial agent. (DOCX) [file pone.0310515.s004.docx]

**Note:** *Denominator for ‘overall percentage’: Total number of patients under surveillance (=216) and for ‘percentage of patients prescribed as empirical therapy’: Total number of patients who received respective antimicrobial agent

**S3 Fig.** Usage of antimicrobial agents as empirical therapy
