# Supplementary material for: “Antimicrobial utilization in a paediatric intensive care unit in India: A step towards strengthening antimicrobial stewardship practices"
Source: PLoS One. 2024 Sep 19;19(9):e0310515. doi: 10.1371/journal.pone.0310515 (PMC11412675; doi:10.1371/journal.pone.0310515)
Supplement: S4 Fig — NOTE: R = RESISTANT, S = SENSITIVE, I = INTERMEDIATE, P = PENICILLIN, OX = OXACILLIN, AM = AMPICILLIN, CRO = CEFTRIAXONE, FEP = CEFEPIME, COT = COTRIMOXAZOLE, VA = VANCOMYCIN, E = ERYTHROMYCIN, DO = DOXYCYCLINE, GM = GENTAMICIN, CM = CLINDAMICIN, NX = NORFLOXACIN, FM = NITROFURANTOIN. (DOCX) [file pone.0310515.s005.docx]

**NOTE:** R= RESISTANT, S= SENSITIVE, I= INTERMEDIATE, P= PENICILLIN, OX= OXACILLIN, AM= AMPICILLIN, CRO= CEFTRIAXONE, FEP= CEFEPIME, COT= COTRIMOXAZOLE, VA= VANCOMYCIN, E=ERYTHROMYCIN, DO= DOXYCYCLINE, GM= GENTAMICIN, CM= CLINDAMICIN, NX= NORFLOXACIN, FM= NITROFURANTOIN

**S4 Fig.** Stacked bar chart showing Gram positive organisms isolated and their sensitivity pattern for the antimicrobials in percentage
