# Supplementary material for: “Antimicrobial utilization in a paediatric intensive care unit in India: A step towards strengthening antimicrobial stewardship practices"
Source: PLoS One. 2024 Sep 19;19(9):e0310515. doi: 10.1371/journal.pone.0310515 (PMC11412675; doi:10.1371/journal.pone.0310515)
Supplement: S5 Fig — NOTE: R = RESISTANT, S = SENSITIVE, I = INTERMEDIATE, P = PENICILLIN, AM = AMPICILLIN, AMC = AMOXICILLIN+CLAVULANATE, TZP = PIPERACILLIN+TAZOBACTAM, CRO = CEFTRIAXONE, CTX = CEFOTAXIME, CAZ = CEFTAZIDIME, CXM = CEFUROXIME, CZ = CEFAZOLIN, CFM = CEFEXIME, FEP = CEFEPIME, COT = COTRIMOXAZOLE, VA = VANCOMYCIN, E = ERYTHROMYCIN, IPM = IMIPENEM, MEM = MEROPENEM, DO = DOXYCYCLINE, AN = AMIKACIN, GM = GENTAMICIN, TM = TOBRAMYCIN, CL = COLISTIN, ATM = AZTREONAM, CM = CLINDAMICIN, LVX = LEVOFLOXACIN, NX = NORFLOXACIN, FM = NITROFURANTOIN. (DOCX) [file pone.0310515.s006.docx]

**NOTE:** R= RESISTANT, S= SENSITIVE, I= INTERMEDIATE, P= PENICILLIN, AM= AMPICILLIN, AMC= AMOXICILLIN+CLAVULANATE, TZP= PIPERACILLIN+TAZOBACTAM, CRO= CEFTRIAXONE, CTX= CEFOTAXIME, CAZ= CEFTAZIDIME, CXM= CEFUROXIME, CZ=CEFAZOLIN, CFM=CEFEXIME, FEP= CEFEPIME, COT= COTRIMOXAZOLE, VA= VANCOMYCIN, E= ERYTHROMYCIN, IPM= IMIPENEM, MEM= MEROPENEM, DO= DOXYCYCLINE, AN= AMIKACIN, GM= GENTAMICIN, TM= TOBRAMYCIN, CL= COLISTIN, ATM= AZTREONAM, CM= CLINDAMICIN, LVX= LEVOFLOXACIN, NX= NORFLOXACIN, FM= NITROFURANTOIN

**S5 Fig.** Stacked bar chart showing Gram negative organisms isolated and their sensitivity pattern for the antimicrobials in percentage
