# Supplementary material for: “Antimicrobial utilization in a paediatric intensive care unit in India: A step towards strengthening antimicrobial stewardship practices"
Source: PLoS One. 2024 Sep 19;19(9):e0310515. doi: 10.1371/journal.pone.0310515 (PMC11412675; doi:10.1371/journal.pone.0310515)
Supplement: S1 File — (DOCX) [file pone.0310515.s007.docx]

**Supporting Information**

**Operational definitions:**

1. **Days of therapy (DOT):** The number of days that a person receives an antimicrobial agent (regardless of the dose).
2. **De-escalation of therapy:** Antimicrobial therapy was considered as de-escalated, if any one of the following criteria were met:
   1. Withdrawal of ≥ 1 antimicrobial agent from empirical therapy
   2. Withdrawal of at least one antimicrobial agent plus addition of narrow spectrum antimicrobial agents
   3. Stopping empirical therapy and switching to narrow spectrum antimicrobial agent
3. **Escalation of therapy:** Antimicrobial therapy was considered as escalated, if any one of the following criteria were met:
   1. Addition of ≥ 1 antimicrobial agent to empiric antimicrobial therapy
   2. Switching from narrow spectrum to broad-spectrum antimicrobial agents
   3. Withdrawal of ≥ 1 antimicrobial agent from empirical therapy, but addition of ≥ 1 broad-spectrum antimicrobial agent to antimicrobial therapy
4. **Empiric therapy**: Empiric therapy was defined as initial therapy started in the absence of definitive microbiologic pathogen identification. Empiric therapy may be mono-, combination, or broad-spectrum, and/or multidrug in nature.
5. **Targeted antimicrobial therapy:** Targeted antimicrobial therapy (or definitive therapy) was defined as therapy targeted to a specific pathogen (usually after microbiologic identification). It may be mono- or combination, but is not intended to be broad-spectrum. Microbiology result can be any culture and/or sensitivity result from a relevant clinical (e.g., blood, sputum, etc.,) [but not screening] specimen as well as any other microbiology result like for example Legionella Urinary Antigen. Patients were counted twice depending on the number of targeted antimicrobials administered for more than one resistant micro-organism.
6. **Access group antimicrobials**: This group includes antibiotics that have activity against a wide range of commonly encountered susceptible pathogens while also showing lower resistance potential than antibiotics in the other groups. Detailed information on the WHO AWaRe classification system is available at <https://www.who.int/publications/i/item/2021-aware-classification>.
7. **Watch group antimicrobials**: This group includes antibiotic classes that have higher resistance potential and includes most of the highest priority agents among the Critically Important Antimicrobials for Human Medicine and/or antibiotics that are at relatively high risk of selection of bacterial resistance. These medicines should be prioritized as key targets of stewardship programs and monitoring. Detailed information on the WHO AWaRe classification system is available at <https://www.who.int/publications/i/item/2021-aware-classification>.
8. **Reserve group antimicrobials**: This group includes antibiotics and antibiotic classes that should be reserved for treatment of confirmed or suspected infections due to multi-drug-resistant organisms. Reserve group antibiotics should be treated as “last resort” options. These antibiotics should be accessible, but their use should be tailored to highly specific patients and settings, when all alternatives have failed or are not suitable. These medicines could be protected and prioritized as key targets of national and international stewardship programs involving monitoring and utilization reporting, to preserve their effectiveness. Detailed information on the WHO AWaRe classification system is available at <https://www.who.int/publications/i/item/2021-aware-classification>.
9. **Incidence Rate of Hospital acquired MRSA, VRSA and VRE based on clinical cultures:** Isolates from first positive cultures of MRSA, VRSA, and VRE identified after 48 hours of hospital admission.
10. **Afebrile**: Patient with body temperature 36.5°C (97.7°F) - 37.5°C (99.5°F) for more than 24 hours.
11. **Readmission**: Patients after getting discharged/transferred to ward from PICU, if getting readmitted in PICU with complaints of Index diagnosis within 7 days of discharge/transfer to ward
12. **In-hospital Mortality**: Death due to any cause in selected patients during their PICU stay.
