# Supplementary material for: “Antimicrobial utilization in a paediatric intensive care unit in India: A step towards strengthening antimicrobial stewardship practices"
Source: PLoS One. 2024 Sep 19;19(9):e0310515. doi: 10.1371/journal.pone.0310515 (PMC11412675; doi:10.1371/journal.pone.0310515)
Supplement: S2 File — (DOCX) [file pone.0310515.s008.docx]

**Datasheet-I**

| **Column** | **Parameter** | **Code** |
| --- | --- | --- |
| A | Patient code | - |
| B | Age in years | - |
| C | Sex | Male=1, Female=2 |
| D | Diagnosis | - |
| E | Intervention | Mechanical Ventilation=1, Central Line=2, Urinary Catheter=3, |
| F | Ventilator days | - |
| G | PICU Stay (in days) | - |
| H | Antimicrobial agent/s (AMA) prescribed | - |
| I | AMA Prescribed by Generic Name | Yes=1, No=0 |
| J | AMA Listed in essential medicine list | NLEM=1, WHO EML=2, Not listed in either list=0 |
| K | WHO AWaRe classification | Access group=1, Watch group=2, Reserve group=3 |
| L | Dosage form | Intravenous=1, Oral/Nasogastric=2 |
| M | Days of Therapy for AMA (in days) | - |
| N | Length of therapy (in days) | - |
| O | Number of antimicrobials of same generic | - |
| P | Frequency | OD=1, BD=2, TID=3, QID=4 |
| Q | Doses Prescribed | - |
| R | Doses actually administered | - |

| **A** | **B** | **C** | **D** | **E** | **F** | **G** | **H** | **I** | **J** | **K** | **L** | **M** | **N** | **O** | **P** | **Q** | **R** |
| --- | --- | --- | --- | --- | --- | --- | --- | --- | --- | --- | --- | --- | --- | --- | --- | --- | --- |
| 001/2019 | 2 | 2 | TBM, RESPIRATORY FAILURE, SHOCK | 1,2,3 | 5 | 5 | CEFTRIAXONE | 1 | 1,2 | 2 | 1 | 5 | 5 | 1 | 3 | 15 | 15 |
|  |  |  |  |  |  |  | MEROPENEM | 1 | 2 | 2 | 1 | 5 |  | 1 | 3 | 15 | 15 |
|  |  |  |  |  |  |  | COLISTIN | 1 | 2 | 3 | 1 | 3 |  | 1 | 3 | 9 | 9 |
| 002/2019 | 11 | 2 | AGN, RPGN, NEPHROTIC SYNDROME, ENCEPHALITIS | 2 | 2 | 18 | CEFTRIAXONE | 1 | 1,2 | 2 | 1 | 5 | 5 | 1 | 2 | 12 | 12 |
| 003/2019 | 2 | 1 | DEMYELINATING D/O, ENCEPHALOMYELITIS, AKI, RESP FAILURE | 1,2,3 | 4 | 4 | MEROPENEM | 1 | 2 | 2 | 1 | 3 | 3 | 1 | 2 | 6 | 6 |
|  |  |  |  |  |  |  | DOXYCYCLINE | 1 | 1,2 | 1 | 2 | 2 |  | 1 | 2 | 4 | 3 |
|  |  |  |  |  |  |  | TEICOPLANIN | 1 | 2 | 2 | 1 | 2 |  | 1 | 2 | 4 | 4 |
| 004/2019 | 1 | 1 | BRONCHIOLITIS, LOWER LOBE PNEUMONIA | 2 | 2 | 3 | AMOXYCILLIN+CLAVULANATE | 1 | 1,2 | 1 | 1 | 1 | 3 | 1 | 3 | 3 | 3 |
|  |  |  |  |  |  |  | CEFTRIAXONE | 1 | 1,2 | 2 | 1 | 2 |  | 1 | 2 | 4 | 2 |
|  |  |  |  |  |  |  | AMIKACIN | 1 | 2 | 1 | 1 | 1 |  | 1 | 1 | 1 | 1 |
| 005/2019 | 11 | 2 | ANASARCA, TI NEPHRITIS, RT CCF, DEXTROCARDIA, PAH | 2,3 | 2 | 12 | CEFTRIAXONE | 1 | 1,2 | 2 | 1 | 11 | 12 | 1 | 2 | 22 | 22 |
|  |  |  |  |  |  |  | AMIKACIN | 1 | 2 | 1 | 1 | 4 |  | 1 | 1 | 4 | 4 |
|  |  |  |  |  |  |  | PIPERACILLIN+TAZOBACTAM | 1 | 1,2 | 2 | 1 | 2 |  | 1 | 3 | 6 | 5 |
| 006/2019 | 2.16 | 2 | POSTOP INTESTINAL OBSTRUCTION, ANEMIA, CC IN SITU | 2 | 2 | 13 | MEROPENEM | 1 | 2 | 2 | 1 | 13 | 13 | 1 | 3 | 39 | 37 |
|  |  |  |  |  |  |  | AMPICILLIN | 1 | 1,2 | 1 | 1 | 12 |  | 1 | 3 | 36 | 34 |
| 007/2019 | 8 | 1 | WILSONS DISEASE, PORTAL HYPERTENSION | 2 | 2 | 3 | CEFOTAXIME | 1 | 1,2 | 2 | 1 | 2 | 2 | 1 | 3 | 3 | 2 |
| 008/2019 | 2 | 2 | VSD, PDA, PAH, PNEUMONIA | 2 | 2 | 4 | AMOXYCILLIN+CLAVULANATE | 1 | 1,2 | 1 | 1 | 4 | 4 | 1 | 3 | 12 | 12 |
| 009/2019 | 2.16 | 1 | LOS, LT OTITIS MEDIA, SEPSIS | 2 | 2 | 6 | CEFOTAXIME | 1 | 1,2 | 2 | 1 | 6 | 6 | 1 | 3 | 18 | 16 |
|  |  |  |  |  |  |  | AMIKACIN | 1 | 2 | 1 | 1 | 5 |  | 1 | 1 | 5 | 5 |
| 010/2019 | 14 | 2 | SHUNT INFECTION, MENINGITIS | 2 | 2 | 4 | COLISTIN | 1 | 2 | 3 | 1 | 4 | 4 | 1 | 3 | 12 | 12 |
|  |  |  |  |  |  |  | MEROPENEM | 1 | 2 | 2 | 1 | 3 |  | 1 | 3 | 9 | 9 |
| 011/2019 | 12 | 2 | ALL | 2 | 2 | 5 | PIPERACILLIN+TAZOBACTAM | 1 | 1,2 | 2 | 1 | 5 | 5 | 1 | 3 | 15 | 14 |
|  |  |  | 3ed |  |  |  | COTRIMOXAZOLE | 2 | 1,2 | 1 | 2 | 4 |  | 1 | 1 | 4 | 4 |
|  |  |  |  |  |  |  | VANCOMYCIN | 1 | 1,2 | 2 | 1 | 5 |  | 1 | 3 | 15 | 14 |
| 012/2019 | 12 | 2 | ALL | 2 | 2 | 3 | PIPERACILLIN+TAZOBACTAM | 1 | 1,2 | 2 | 1 | 1 | 3 | 1 | 3 | 3 | 2 |
|  |  |  |  |  |  |  | VANCOMYCIN | 1 | 1,2 | 2 | 1 | 3 |  | 1 | 3 | 9 | 7 |
|  |  |  |  |  |  |  | MEROPENEM | 1 | 2 | 2 | 1 | 3 |  | 1 | 3 | 9 | 7 |
|  |  |  |  |  |  |  | COTRIMOXAZOLE | 1 | 1,2 | 1 | 2 | 3 |  | 1 | 1 | 3 | 2 |
|  |  |  |  |  |  |  | AZITHROMYCIN | 1 | 1,2 | 2 | 2 | 2 |  | 1 | 1 | 2 | 2 |
| 013/2019 | 12 | 1 | SEPTIC ARTHRITIS | 2 | 2 | 6 | CEFTRIAXONE | 1 | 1,2 | 2 | 1 | 6 | 6 | 1 | 2 | 12 | 12 |
|  |  |  |  |  |  |  | VANCOMYCIN | 1 | 1,2 | 2 | 1 | 5 |  | 1 | 3 | 15 | 14 |
|  |  |  |  |  |  |  | CLINDAMYCIN | 1 | 1,2 | 1 | 1 | 4 |  | 1 | 3 | 12 | 11 |
|  |  |  |  |  |  |  | RIFAMPICIN | 1 | 1,2 | 2 | 2 | 3 |  | 1 | 2 | 6 | 5 |
| 014/2019 | 13 | 1 | SCD, AKI | 2 | 2 | 6 | MEROPENEM | 1 | 2 | 2 | 1 | 6 | 6 | 1 | 2 | 12 | 11 |
|  |  |  |  |  |  |  | VANCOMYCIN | 1 | 1,2 | 2 | 1 | 4 |  | 1 | 2 | 8 | 6 |
|  |  |  |  |  |  |  | TEICOPLANIN | 1 | 2 | 2 | 1 | 3 |  | 1 | 2 | 6 | 5 |
| 015/2019 | 2.8 | 2 | PNEUMONIA | 2 | 2 | 5 | AMPICILLIN | 1 | 1,2 | 1 | 1 | 5 | 5 | 1 | 4 | 22 | 16 |
|  |  |  |  |  |  |  | GENTAMICIN | 1 | 1,2 | 1 | 1 | 5 |  | 1 | 1 | 5 | 5 |
| 016/2019 | 6 | 1 | PYOGENIC MENINGITIS | 2 | 2 | 6 | CEFTRIAXONE | 1 | 1,2 | 2 | 1 | 6 | 6 | 1 | 2 | 12 | 11 |
| 017/2019 | 7 | 1 | SCD | 2 | 2 | 6 | CEFTRIAXONE | 1 | 1,2 | 2 | 1 | 6 | 6 | 1 | 2 | 12 | 11 |
| 018/2019 | 7 | 1 | SCD | 2 | 12 | 12 | MEROPENEM | 1 | 2 | 2 | 1 | 12 | 12 | 1 | 3 | 36 | 32 |
|  |  |  |  |  |  |  | TEICOPLANIN | 1 | 2 | 2 | 1 | 12 |  | 1 | 2 | 24 | 24 |
| 019/2019 | 4 | 2 | POST OP CRANIOPHARYNGIOMA | 2,3 | 2 | 9 | CEFTRIAXONE | 1 | 1,2 | 2 | 1 | 9 | 9 | 1 | 2 | 18 | 16 |
| 020/2019 | 2.8 | 1 | MENINGITIS, SEIZURE | 2 | 2 | 5 | CEFOTAXIME | 1 | 1,2 | 2 | 1 | 1 | 5 | 1 | 4 | 4 | 1 |
|  |  |  |  |  |  |  | MEROPENEM | 1 | 2 | 2 | 1 | 5 |  | 1 | 3 | 15 | 13 |
|  |  |  |  |  |  |  | VANCOMYCIN | 1 | 1,2 | 2 | 1 | 5 |  | 1 | 3 | 15 | 13 |
| 021/2019 | 1.16 | 2 | BRONCHOPNEUMONIA, FTT, CARDIOMEGALY | 2 | 2 | 3 | MEROPENEM | 1 | 2 | 2 | 1 | 3 | 3 | 1 | 3 | 9 | 8 |
|  |  |  |  |  |  |  | VANCOMYCIN | 1 | 1,2 | 2 | 1 | 3 |  | 1 | 3 | 9 | 8 |
| 022/2019 | 8 | 1 | POST OP CRANIOTOMY | 2 | 2 | 4 | CEFTRIAXONE | 1 | 1,2 | 2 | 1 | 4 | 4 | 1 | 2 | 8 | 6 |
|  |  |  |  |  |  |  | AMIKACIN | 1 | 2 | 1 | 1 | 4 |  | 1 | 1 | 4 | 3 |
| 023/2019 | 13 | 2 | PNEUMONIA, R. HEMIPARESIS | 2 | 2 | 8 | CEFTRIAXONE | 1 | 1,2 | 2 | 1 | 8 | 8 | 1 | 1 | 16 | 14 |
| 024/2019 | 11 | 2 | ACUTE ENCEPHALITIS, MENINGITIS | 2 | 2 | 3 | CEFTRIAXONE | 1 | 1,2 | 2 | 1 | 3 | 3 | 1 | 2 | 6 | 6 |
|  |  |  |  |  |  |  | VANCOMYCIN | 1 | 1,2 | 2 | 1 | 3 |  | 1 | 3 | 9 | 7 |
|  |  |  |  |  |  |  | DOXYCYCLINE | 1 | 1,2 | 1 | 2 | 3 |  | 1 | 2 | 6 | 5 |
| 025/2019 | 4 | 2 | PLEURAL EFFUSION, BURKITTS LYMPHOMA | 2 | 2 | 3 | CEFTRIAXONE | 1 | 1,2 | 2 | 1 | 3 | 3 | 1 | 2 | 9 | 8 |
|  |  |  |  |  |  |  | VANCOMYCIN | 1 | 1,2 | 2 | 1 | 3 |  | 1 | 3 | 6 | 4 |
| 026/2019 | 11 | 2 | CYSTIC FIBROSIS, PNEUMONIA | 2 | 2 | 23 | COLISTIN | 1 | 2 | 3 | 1 | 11 | 17 | 1 | 3 | 3 | 33 |
|  |  |  |  |  |  |  | VANCOMYCIN | 1 | 1,2 | 2 | 1 | 3 |  | 1 | 3 | 3 | 9 |
|  |  |  |  |  |  |  | AZITHROMYCIN | 1 | 1,2 | 2 | 2 | 22 |  | 1 | 1 | 1 | 22 |
|  |  |  |  |  |  |  | TEICOPLANIN | 1 | 2 | 2 | 1 | 4 |  | 1 | 1 | 1 | 5 |
|  |  |  |  |  |  |  | LEVOFLOXACIN | 1 | 1,2 | 2 | 2 | 3 |  | 1 | 1 | 1 | 3 |
|  |  |  |  |  |  |  | LINEZOLID | 1 | 1,2 | 3 | 1 | 17 |  | 1 | 2 | 2 | 34 |
|  |  |  |  |  |  |  | TIGECYCLINE | 1 | 2 | 3 | 1 | 13 |  | 1 | 2 | 2 | 26 |
|  |  |  |  |  |  |  | COLISTIN | 1 | 2 | 3 | 1 | 7 |  | 2 | 3 | 3 | 21 |
|  |  |  |  |  |  |  | AZTREONAM | 1 | 2 | 3 | 1 | 5 |  | 1 | 3 | 3 | 15 |
| 027/2019 | 11 | 2 | CYSTIC FIBROSIS, PNEUMONIA | 2 | 2 | 16 | LINEZOLID | 1 | 1,2 | 3 | 1 | 4 | 17 | 1 | 3 | 12 | 12 |
|  |  |  |  |  |  |  | AZTREONAM | 1 | 2 | 3 | 1 | 4 |  | 1 | 3 | 12 | 12 |
|  |  |  |  |  |  |  | AZITHROMYCIN | 1 | 1,2 | 2 | 2 | 16 |  | 1 | 1 | 16 | 16 |
|  |  |  |  |  |  |  | LINEZOLID | 1 | 1,2 | 3 | 2 | 4 |  | 2 | 1 | 4 | 4 |
|  |  |  |  |  |  |  | COLISTIN | 1 | 2 | 3 | 1 | 7 |  | 1 | 2 | 14 | 13 |
| 028/2019 | 4 | 1 | PNEUMONIA | 2 | 2 | 7 | CEFTRIAXONE | 1 | 1,2 | 2 | 1 | 1 | 4 | 1 | 2 | 2 | 2 |
|  |  |  |  |  |  |  | AMOXYCILLIN+CLAVULANATE | 1 | 1,2 | 1 | 2 | 2 |  | 1 | 3 | 6 | 6 |
| 029/2019 | 2.17 | 1 | VSD, BRONCHIOLITIS, SEVERE PAH | 2 | 2 | 5 | AMPICILLIN+CLOXACILLIN | 2 | 1,2 | 1 | 1 | 3 | 3 | 1 | 3 | 9 | 7 |
|  |  |  |  |  |  |  | AMIKACIN | 1 | 2 | 1 | 1 | 3 |  | 1 | 1 | 3 | 3 |
| 030/2019 | 1 | 1 | PNEUMONIA, SEVERE MALNUTRITION | 2 | 2 | 5 | CEFTRIAXONE | 1 | 1,2 | 2 | 1 | 5 | 5 | 1 | 2 | 12 | 9 |
|  |  |  |  |  |  |  | AMIKACIN | 1 | 2 | 1 | 1 | 5 |  | 1 | 1 | 12 | 12 |
|  |  |  |  |  |  |  |  |  |  |  |  |  |  |  |  |  |  |
| 031/2019 | 2.3 | 2 | ACHD, PNEUMONIA | 2 | 2 | 7 | CEFTRIAXONE | 1 | 1,2 | 2 | 1 | 5 | 5 | 1 | 2 | 12 | 12 |
|  |  |  |  |  |  |  | CLINDAMYCIN | 1 | 1,2 | 1 | 1 | 4 |  | 1 | 3 | 12 | 12 |
|  |  |  |  |  |  |  | CEFPODOXIME | 1 | 2 | 2 | 2 | 2 |  | 1 | 2 | 4 | 4 |
| 032/2019 | 2.25 | 2 | LRTI, FEBRILE SEIZURES, MENINGITIS | 2 | 2 | 6 | CEFTRIAXONE | 1 | 1,2 | 2 | 1 | 6 | 6 | 1 | 2 | 12 | 12 |
| 033/2019 | 12 | 1 | NEPHROTIC SYNDROME | 2 | 2 | 3 | AMOXYCILLIN | 1 | 1,2 | 1 | 2 | 3 | 3 | 1 | 3 | 9 | 7 |
| 034/2019 | 8 | 1 | ADEM, DEHYDRATION | 2 | 2 | 5 | CEFTRIAXONE | 1 | 1,2 | 2 | 1 | 2 | 5 | 1 | 2 | 4 | 4 |
|  |  |  |  |  |  |  | METRONIDAZOLE | 1 | 1,2 | 1 | 1 | 2 |  | 1 | 3 | 6 | 6 |
|  |  |  |  |  |  |  | AMPICILLIN+CLOXACILLIN | 1 | 2 | 1 | 1 | 3 |  | 1 | 4 | 12 | 12 |
|  |  |  |  |  |  |  | AMOXYCILLIN+CLAVULANATE | 1 | 1,2 | 1 | 2 | 2 |  | 1 | 3 | 6 | 7 |
| 035/2019 | 7 | 2 | POST OP POST FOSSA LESION | 2 | 2 | 3 | CEFTRIAXONE | 1 | 1,2 | 2 | 1 | 3 | 3 | 1 | 2 | 6 | 5 |
|  |  |  |  |  |  |  | AMIKACIN | 1 | 2 | 1 | 1 | 3 |  | 1 | 1 | 3 | 3 |
| 036/2019 | 2.16 | 2 | FTT, DEHYDRATION | 2 | 2 | 16 | AMPICILLIN | 1 | 1,2 | 1 | 1 | 1 | 16 | 1 | 4 | 4 | 1 |
|  |  |  |  |  |  |  | GENTAMICIN | 1 | 1,2 | 1 | 1 | 1 |  | 1 | 3 | 3 | 1 |
|  |  |  |  |  |  |  | CEFTRIAXONE | 1 | 1,2 | 2 | 1 | 4 |  | 1 | 2 | 8 | 8 |
|  |  |  |  |  |  |  | PIPERACILLIN+TAZOBACTAM | 2 | 1,2 | 2 | 1 | 1 |  | 1 | 3 | 3 | 1 |
|  |  |  |  |  |  |  | MEROPENEM | 1 | 2 | 2 | 1 | 12 |  | 1 | 3 | 36 | 36 |
|  |  |  |  |  |  |  | VANCOMYCIN | 1 | 1,2 | 2 | 1 | 12 |  | 1 | 3 | 36 | 36 |
| 037/2019 | 6 | 1 | R. EMPYEMA | 2 | 2 | 3 | CEFTRIAXONE | 1 | 1,2 | 2 | 1 | 3 | 3 | 1 | 2 | 6 | 6 |
|  |  |  |  |  |  |  | AMPICILLIN+CLOXACILLIN | 1 | 2 | 1 | 1 | 3 |  | 1 | 3 | 9 | 9 |
|  |  |  |  |  |  |  | AMIKACIN | 1 | 2 | 1 | 1 | 3 |  | 1 | 1 | 3 | 3 |
| 038/2019 | 4 | 1 | POST OP E.L. | 2 | 2 | 5 | METRONIDAZOLE | 1 | 1,2 | 1 | 1 | 5 | 5 | 1 | 3 | 15 | 15 |
|  |  |  |  |  |  |  | AMIKACIN | 1 | 2 | 1 | 1 | 5 |  | 1 | 1 | 1 | 1 |
|  |  |  |  |  |  |  | MEROPENEM | 1 | 2 | 2 | 1 | 5 |  | 1 | 3 | 15 | 15 |
| 039/2019 | 12 | 1 | FEVER, PNEUMONIA | 2 | 2 | 7 | CEFTRIAXONE | 1 | 1,2 | 2 | 1 | 1 | 7 | 1 | 2 | 2 | 2 |
|  |  |  |  |  |  |  | VANCOMYCIN | 1 | 1,2 | 2 | 1 | 1 |  | 1 | 3 | 3 | 3 |
|  |  |  |  |  |  |  | AZITHROMYCIN | 1 | 1,2 | 2 | 2 | 6 |  | 1 | 1 | 6 | 6 |
|  |  |  |  |  |  |  | TEICOPLANIN | 1 | 2 | 2 | 1 | 6 |  | 1 | 3 | 18 | 16 |
| 042/2019 | 2.33 | 1 | ACUTE BRONCHIOLITIS | 2 | 2 | 9 | CEFTRIAXONE | 1 | 1,2 | 2 | 1 | 5 | 5 | 1 | 2 | 12 | 8 |
|  |  |  |  |  |  |  | AMIKACIN | 1 | 2 | 1 | 1 | 5 |  | 1 | 1 | 5 | 5 |
| 041/2019 | 14 | 1 | B.L. LOBAR PNEUMONIA | 2 | 2 | 5 | CEFTRIAXONE | 1 | 1,2 | 2 | 1 | 4 | 4 | 1 | 2 | 8 | 8 |
|  |  |  |  |  |  |  | AMIKACIN | 1 | 2 | 1 | 1 | 4 |  | 1 | 1 | 4 | 4 |
|  |  |  |  |  |  |  | AZITHROMYCIN | 1 | 1,2 | 2 | 2 | 4 |  | 1 | 1 | 4 | 4 |
| 042/2019 | 2.66 | 1 | LARYNGOMALACIA, URTI | 1,3 | 3 | 8 | AMPICILLIN | 1 | 1,2 | 1 | 1 | 1 | 4 | 1 | 3 | 3 | 1 |
|  |  |  |  |  |  |  | GENTAMICIN | 1 | 1,2 | 1 | 1 | 1 |  | 1 | 2 | 2 | 1 |
|  |  |  |  |  |  |  | CEFOTAXIME | 1 | 1,2 | 2 | 1 | 2 |  | 1 | 2 | 4 | 5 |
|  |  |  |  |  |  |  | AMIKACIN | 1 | 2 | 1 | 1 | 2 |  | 1 | 1 | 2 | 2 |
| 043/2020 | 2.16 | 2 | BRONCHOPNEUMONIA, MYOCARDITIS | 1 | 4 | 11 | PIPERACILLIN+TAZOBACTAM | 2 | 1,2 | 2 | 1 | 4 | 11 | 1 | 3 | 12 | 12 |
|  |  |  |  |  |  |  | AMIKACIN | 1 | 2 | 1 | 1 | 3 |  | 1 | 1 | 3 | 3 |
|  |  |  |  |  |  |  | AZITHROMYCIN | 1 | 1,2 | 2 | 1 | 4 |  | 1 | 1 | 4 | 4 |
|  |  |  |  |  |  |  | MEROPENEM | 1 | 2 | 2 | 1 | 5 |  | 1 | 3 | 15 | 15 |
|  |  |  |  |  |  |  | VANCOMYCIN | 1 | 1,2 | 2 | 1 | 1 |  | 1 | 3 | 3 | 3 |
|  |  |  |  |  |  |  | COLISTIN | 1 | 2 | 3 | 1 | 5 |  | 1 | 3 | 15 | 15 |
|  |  |  |  |  |  |  | AZITHROMYCIN | 1 | 1,2 | 2 | 1 | 1 | 7 | 1 | 2 | 2 | 2 |
| 044/2020 | 2.83 | 2 | BRONCHOPNEUMONIA | 2 | 2 | 3 | AZITHROMYCIN | 1 | 1,2 | 2 | 1 | 3 | 3 | 1 | 1 | 3 | 2 |
|  |  |  |  |  |  |  | CEFTRIAXONE | 1 | 1,2 | 2 | 1 | 3 |  | 1 | 2 | 6 | 5 |
| 045/2020 | 3 | 2 | PNEUMONIA, RT. EMPYEMA, PNEUMOTHORAX | 1,2 | 7 | 8 | PIPERACILLIN+TAZOBACTAM | 2 | 1,2 | 2 | 1 | 3 | 7 | 1 | 3 | 9 | 5 |
|  |  |  |  |  |  |  | VANCOMYCIN | 1 | 1,2 | 2 | 1 | 7 |  | 1 | 3 | 21 | 19 |
|  |  |  |  |  |  |  | AZITHROMYCIN | 1 | 1,2 | 2 | 1 | 5 |  | 1 | 1 | 5 | 5 |
|  |  |  |  |  |  |  | COLISTIN | 1 | 2 | 3 | 1 | 5 |  | 1 | 3 | 15 | 14 |
| 046/2020 | 7 | 1 | CP, SEIZURE, GDD, ASPIRATIONAL PNEUMONIA | 2 | 2 | 15 | CEFTRIAXONE | 1 | 2 | 2 | 1 | 7 | 7 | 1 | 2 | 14 | 13 |
| 047/2020 | 2.25 | 2 | VSD, ASD, BRONCHIOLITIS, CCF | 2 | 2 | 16 | CEFTRIAXONE | 1 | 1,2 | 2 | 1 | 2 | 8 | 1 | 2 | 4 | 3 |
|  |  |  |  |  |  |  | AMIKACIN | 1 | 2 | 1 | 1 | 1 |  | 1 | 1 | 1 | 1 |
|  |  |  |  |  |  |  | AZITHROMYCIN | 1 | 1,2 | 2 | 2 | 6 |  | 1 | 1 | 6 | 6 |
|  |  |  |  |  |  |  | CEFPODOXIME | 1 | 2 | 2 | 2 | 7 |  | 1 | 2 | 14 | 14 |
| 048/2020 | 12 | 1 | SCD, VASOOCCLUSIVE CRISIS | 2 | 2 | 4 | CEFOTAXIME | 1 | 1,2 |  | 1 | 4 | 4 | 1 | 3 | 12 | 12 |
| 049/2020 | 2 | 1 | B CELL ALL, PNEUMONIA | 2 | 2 | 12 | PIPERACILLIN+TAZOBACTAM | 2 | 1,2 | 2 | 1 | 2 | 12 | 1 | 3 | 6 | 5 |
|  |  |  |  |  |  |  | VANCOMYCIN | 1 | 1,2 | 2 | 1 | 9 |  | 1 | 3 | 27 | 24 |
|  |  |  |  |  |  |  | AMIKACIN | 1 | 2 | 1 | 1 | 2 |  | 1 | 1 | 2 | 2 |
|  |  |  |  |  |  |  | MEROPENEM | 1 | 2 | 2 | 1 | 7 |  | 1 | 3 | 21 | 19 |
|  |  |  |  |  |  |  | COTRIMOXAZOLE | 2 | 1,2 | 1 | 2 | 3 |  | 1 | 1 | 3 | 3 |
|  |  |  |  |  |  |  | COLISTIN | 1 | 2 | 3 | 1 | 2 |  | 1 | 3 | 6 | 5 |
| 052/2020 | 2.25 | 1 | MENINGITIS, SEVERE SEPSIS | 2 | 2 | 11 | MEROPENEM | 1 | 2 | 2 | 1 | 4 | 11 | 1 | 3 | 12 | 12 |
|  |  |  |  |  |  |  | VANCOMYCIN | 1 | 1,2 | 2 | 1 | 8 |  | 1 | 3 | 24 | 23 |
|  |  |  |  |  |  |  | AZITHROMYCIN | 1 | 1,2 | 2 | 1 | 3 |  | 1 | 1 | 3 | 3 |
|  |  |  |  |  |  |  | COLISTIN | 1 | 2 | 3 | 1 | 8 |  | 1 | 3 | 24 | 21 |
| 051/2020 | 2.16 | 2 | CONGENITAL HEMOPHAGOCYTIC LYMPHOHISTIOCYTOSIS, SHOCK | 1,2,3 | 5 | 5 | VANCOMYCIN | 1 | 1,2 | 2 | 1 | 9 | 5 | 1 | 4 | 36 | 34 |
|  |  |  |  |  |  |  | MEROPENEM | 1 | 2 | 2 | 1 | 3 |  | 1 | 3 | 9 | 5 |
|  |  |  |  |  |  |  | AMIKACIN | 1 | 2 | 1 | 1 | 2 |  | 1 | 1 | 2 | 2 |
|  |  |  |  |  |  |  | COLISTIN | 1 | 2 | 3 | 1 | 3 |  | 1 | 3 | 9 | 4 |
|  |  |  |  |  |  |  | PIPERACILLIN+TAZOBACTAM | 2 | 1,2 | 2 | 1 | 7 |  | 1 | 3 | 21 | 22 |
| 052/2020 | 2 | 1 | ACCIDENTAL STRANGULATION, HII | 1,2,3 | 9 | 13 | VANCOMYCIN | 1 | 1,2 | 2 | 1 | 13 | 13 | 1 | 3 | 39 | 37 |
|  |  |  |  |  |  |  | MEROPENEM | 1 | 2 | 2 | 1 | 4 |  | 1 | 3 | 12 | 12 |
|  |  |  |  |  |  |  | COLISTIN | 1 | 2 | 3 | 1 | 5 |  | 1 | 3 | 15 | 15 |
| 053/2020 | 2 | 1 | PNEUMONIA | 1,2,3 | 11 | 14 | CEFTRIAXONE | 1 | 1,2 | 2 | 1 | 4 | 14 | 1 | 2 | 8 | 9 |
|  |  |  |  |  |  |  | VANCOMYCIN | 1 | 1,2 | 2 | 1 | 8 |  | 1 | 3 | 24 | 23 |
|  |  |  |  |  |  |  | MEROPENEM | 1 | 2 | 2 | 1 | 3 |  | 1 | 3 | 9 | 9 |
|  |  |  |  |  |  |  | COLISTIN | 1 | 2 | 3 | 1 | 4 |  | 1 | 3 | 12 | 12 |
|  |  |  |  |  |  |  | PIPERACILLIN+TAZOBACTAM | 2 | 1,2 | 2 | 1 | 6 |  | 1 | 3 | 18 | 17 |
| 054/2020 | 12 | 2 | POST OP THORACOTOMY | 2 | 2 | 4 | PIPERACILLIN+TAZOBACTAM | 2 | 1,2 | 2 | 1 | 4 | 4 | 1 | 3 | 12 | 12 |
|  |  |  |  |  |  |  | CLINDAMYCIN | 1 | 1,2 | 1 | 1 | 4 |  | 1 | 3 | 12 | 12 |
| 055/2020 | 2.5 | 2 | GDD, DISMORPHIC SEIZURE DISORDER | 2 | 2 | 8 | VANCOMYCIN | 1 | 1,2 | 2 | 1 | 2 | 8 | 1 | 3 | 6 | 2 |
|  |  |  |  |  |  |  | MEROPENEM | 1 | 2 | 2 | 1 | 6 |  | 1 | 3 | 18 | 17 |
| 056/2020 | 3 | 2 | POST OP L. FRONTAL ABSCESS | 2 | 2 | 4 | CEFTRIAXONE | 1 | 1,2 | 2 | 1 | 3 | 4 | 1 | 2 | 6 | 6 |
|  |  |  |  |  |  |  | AMIKACIN | 1 | 2 | 1 | 1 | 2 |  | 1 | 2 | 4 | 4 |
|  |  |  |  |  |  |  | METRONIDAZOLE | 1 | 1,2 | 1 | 1 | 3 |  | 1 | 3 | 9 | 7 |
| 057/2020 | 11 | 1 | ACUTE ENCEPHALITIS SYNDROME | 1,2,3 | 6 | 13 | VANCOMYCIN | 1 | 1,2 | 2 | 1 | 7 | 14 | 1 | 3 | 21 | 21 |
|  |  |  |  |  |  |  | MEROPENEM | 1 | 2 | 2 | 1 | 3 |  | 1 | 3 | 9 | 7 |
|  |  |  |  |  |  |  | AMIKACIN | 1 | 2 | 1 | 1 | 1 |  | 1 | 1 | 1 | 1 |
|  |  |  |  |  |  |  | COLISTIN | 1 | 2 | 3 | 1 | 7 |  | 1 | 3 | 21 | 21 |
|  |  |  |  |  |  |  | PIPERACILLIN+TAZOBACTAM | 2 | 1,2 | 2 | 1 | 1 |  | 1 | 3 | 3 | 2 |
| 058/2020 | 3.5 | 1 | ORIF FEMUR #, SPASTIC QUADRIPLEGIA | 2 | 2 | 3 | CEFTRIAXONE | 1 | 1,2 | 2 | 1 | 2 | 3 | 1 | 2 | 4 | 4 |
|  |  |  |  |  |  |  | VANCOMYCIN | 1 | 1,2 | 2 | 1 | 2 |  | 1 | 3 | 6 | 6 |
|  |  |  |  |  |  |  | AMIKACIN | 1 | 2 | 1 | 1 | 1 |  | 1 | 1 | 1 | 1 |
|  |  |  |  |  |  |  | PIPERACILLIN+TAZOBACTAM | 2 | 1,2 | 2 | 1 | 1 |  | 1 | 3 | 3 | 2 |
| 059/2020 | 2.16 | 2 | ACUTE BRONCHIOLITIS | 2 | 2 | 6 | AZITHROMYCIN | 1 | 1,2 | 2 | 2 | 6 | 6 | 1 | 1 | 6 | 6 |
|  |  |  |  |  |  |  | GENTAMICIN | 1 | 1,2 | 2 | 1 | 1 |  | 1 | 2 | 2 | 2 |
|  |  |  |  |  |  |  | AMPICILLIN | 1 | 1,2 | 1 | 1 | 1 |  | 1 | 4 | 4 | 1 |
| 060/2020 | 6 | 2 | R. UL PNEUMONIA | 2 | 2 | 5 | CEFTRIAXONE | 1 | 1,2 | 2 | 1 | 3 | 5 | 1 | 2 | 6 | 4 |
|  |  |  |  |  |  |  | GENTAMICIN | 1 | 1,2 | 1 | 1 | 5 |  | 1 | 2 | 12 | 12 |
|  |  |  |  |  |  |  | AMPICILLIN | 1 | 1,2 | 1 | 1 | 2 |  | 1 | 3 | 6 | 5 |
|  |  |  |  |  |  |  | AMOXYCILLIN+CLAVULANATE | 1 | 1,2 | 1 | 1 | 2 |  | 1 | 2 | 4 | 2 |
|  |  |  |  |  |  |  | CLOXACILLIN | 1 | 1,2 | 1 | 1 | 3 |  | 1 | 4 | 12 | 6 |
|  |  |  |  |  |  |  | CLINDAMYCIN | 1 | 1,2 | 1 | 1 | 3 |  | 1 | 3 | 9 | 5 |
| 061/2020 | 6 | 1 | AFP, GBS | 1,2,3 | 5 | 19 | AMPICILLIN | 1 | 1,2 | 1 | 1 | 6 | 6 | 1 | 3 | 18 | 17 |
| 062/2020 | 2.8 | 2 | EXTRAHEPATIC BILIARY ATRESIA, LIVER FAILURE | 2 | 2 | 4 | CEFOTAXIME | 1 | 1,2 | 2 | 1 | 4 | 4 | 1 | 3 | 12 | 8 |
|  |  |  |  |  |  |  | CLOXACILLIN | 1 | 1,2 | 1 | 1 | 4 |  | 1 | 4 | 16 | 12 |
| 063/2020 | 2.28 | 1 | CHPS, SEPSIS | 2 | 2 | 4 | MEROPENEM | 1 | 2 | 2 | 1 | 2 | 4 | 1 | 3 | 6 | 4 |
|  |  |  |  |  |  |  | AMIKACIN | 1 | 2 | 1 | 1 | 3 |  | 1 | 1 | 3 | 3 |
|  |  |  |  |  |  |  | PIPERACILLIN+TAZOBACTAM | 2 | 1,2 | 2 | 1 | 3 |  | 1 | 3 | 9 | 11 |
| 064/2020 | 7 | 2 | SRNS, FSGS, AKI | 2 | 2 | 3 | AMPICILLIN | 1 | 1,2 | 1 | 1 | 3 | 3 | 1 | 3 | 9 | 7 |
| 065/2020 | 12 | 2 | SEPTIC ARTHRITIS HIP RT | 2 | 2 | 3 | CEFTRIAXONE | 1 | 1,2 | 2 | 1 | 3 | 3 | 1 | 2 | 6 | 4 |
|  |  |  |  |  |  |  | AMIKACIN | 1 | 2 | 1 | 1 | 1 |  | 1 | 1 | 1 | 1 |
|  |  |  |  |  |  |  | AMPICILLIN+CLOXACILLIN | 1 | 2 | 1 | 1 | 3 |  | 1 | 4 | 12 | 6 |
| 066/2020 | 8 | 1 | R UL CONSOLIDATION, PNEUMONIA | 1,2,3 | 12 | 16 | CEFTRIAXONE | 1 | 1,2 | 2 | 1 | 15 | 16 | 1 | 2 | 32 | 29 |
|  |  |  |  |  |  |  | VANCOMYCIN | 1 | 1,2 | 2 | 1 | 11 |  | 1 | 3 | 33 | 32 |
|  |  |  |  |  |  |  | AMIKACIN | 1 | 2 | 1 | 1 | 1 |  | 1 | 1 | 1 | 1 |
|  |  |  |  |  |  |  | AMIKACIN | 1 | 2 | 1 | 1 | 4 |  | 2 | 1 | 4 | 4 |
|  |  |  |  |  |  |  | AMPICILLIN+CLOXACILLIN | 1 | 2 | 1 | 1 | 5 |  | 1 | 4 | 22 | 19 |
| 067/2020 | 5 | 1 | FEBRILE NEUTROPENIA, DISSEMINATED NEUROBLASTOMA | 2 | 2 | 3 | COTRIMOXAZOLE | 2 | 1,2 | 1 | 2 | 1 | 3 | 1 | 1 | 1 | 1 |
|  |  |  |  |  |  |  | TEICOPLANIN | 1 | 2 | 2 | 1 | 2 |  | 1 | 1 | 2 | 2 |
|  |  |  |  |  |  |  | MEROPENEM | 1 | 2 | 2 | 1 | 2 |  | 1 | 2 | 4 | 3 |
| 068/2020 | 2 | 1 | SEVERE DEHYDRATION | 2 | 2 | 3 | CEFTRIAXONE | 1 | 1,2 | 2 | 1 | 3 | 3 | 1 | 2 | 6 | 6 |
| 069/2020 | 9 | 1 | R EMPYEMA | 1,2,3 | 5 | 9 | CEFTRIAXONE | 1 | 1,2 | 2 | 1 | 9 | 9 | 1 | 1 | 9 | 7 |
|  |  |  |  |  |  |  | VANCOMYCIN | 1 | 1,2 | 2 | 1 | 8 |  | 1 | 3 | 24 | 21 |
|  |  |  |  |  |  |  | AMIKACIN | 1 | 2 | 1 | 1 | 5 |  | 1 | 1 | 5 | 5 |
|  |  |  |  |  |  |  | AMPICILLIN+CLOXACILLIN | 1 | 2 | 1 | 1 | 2 |  | 1 | 4 | 8 | 5 |
| 070/2020 | 8 | 2 | AUTOIMMUNE HEPATITIS, HEPATIC ENCEPHALOPATHY | 3 | 2 | 8 | CEFOTAXIME | 1 | 1,2 | 2 | 1 | 9 | 9 | 1 | 3 | 27 | 23 |
|  |  |  |  |  |  |  | CLOXACILLIN | 1 | 1,2 | 1 | 1 | 8 |  | 1 | 4 | 32 | 27 |
|  |  |  |  |  |  |  | RIFAXIMIN | 1 | 2 | 2 | 2 | 8 |  | 1 | 3 | 24 | 22 |
| 071/2020 | 2.28 | 2 | BRONCHIOLITIS | 3 | 2 | 6 | CEFTRIAXONE | 1 | 1,2 |  | 1 | 2 | 6 | 1 | 1 | 2 | 1 |
|  |  |  |  |  |  |  | VANCOMYCIN | 1 | 1,2 |  | 1 | 5 |  | 1 | 3 | 15 | 12 |
|  |  |  |  |  |  |  | AZITHROMYCIN | 1 | 1,2 |  | 2 | 5 |  | 1 | 1 | 5 | 5 |
| 072/2020 | 2.16 | 2 | CHOANAL ATRESIA, PNEUMONIA, CCF | 1,2 | 14 | 15 | VANCOMYCIN | 1 | 1,2 |  | 1 | 2 | 8 | 1 | 3 | 6 | 3 |
|  |  |  |  |  |  |  | MEROPENEM | 1 | 2 |  | 1 | 8 |  | 1 | 3 | 24 | 24 |
| 073/2020 | 12 | 1 | PRE-B CELL ALL, VE2OCCLUSVE DISEASE | 2 | 2 | 7 | COTRIMOXAZOLE | 2 | 1,2 |  | 2 | 6 | 6 | 1 | 1 | 6 | 6 |
|  |  |  |  |  |  |  | VANCOMYCIN | 1 | 1,2 |  | 1 | 6 |  | 1 | 3 | 18 | 18 |
|  |  |  |  |  |  |  | MEROPENEM | 1 | 2 |  | 1 | 6 |  | 1 | 3 | 18 | 18 |
| 074/2020 | 4 | 1 | R. EMPYEMA, CONSOLIDATION | 2 | 2 | 4 | CEFTRIAXONE | 1 | 1,2 |  | 1 | 3 | 3 | 1 | 2 | 6 | 6 |
|  |  |  |  |  |  |  | AMIKACIN | 1 | 2 |  | 1 | 3 |  | 1 | 1 | 3 | 3 |
|  |  |  |  |  |  |  | AMPICILLIN+CLOXACILLIN | 1 | 2 |  | 1 | 3 |  | 1 | 4 | 12 | 11 |
| 075/2020 | 6 | 2 | POST OP LAMINECTOMY D12-S1 | 2 | 2 | 4 | CEFTRIAXONE | 1 | 1,2 |  | 1 | 3 | 4 | 1 | 2 | 6 | 5 |
|  |  |  |  |  |  |  | AMIKACIN | 1 | 2 |  | 1 | 2 |  | 1 | 1 | 2 | 2 |
| 076/2020 | 2.28 | 1 | PYOGENIC MENINGITIS | 2 | 2 | 12 | VANCOMYCIN | 1 | 1,2 |  | 1 | 5 | 12 | 1 | 3 | 15 | 15 |
|  |  |  |  |  |  |  | MEROPENEM | 1 | 2 |  | 1 | 12 |  | 1 | 3 | 32 | 28 |
|  |  |  |  |  |  |  | AMIKACIN | 1 | 2 |  | 1 | 4 |  | 1 | 1 | 4 | 4 |
| 077/2020 | 2.4 | 1 | FTT, SAM, GDD | 2 | 2 | 12 | VANCOMYCIN | 1 | 1,2 |  | 1 | 5 | 12 | 1 | 3 | 15 | 12 |
|  |  |  |  |  |  |  | AMIKACIN | 1 | 2 |  | 1 | 2 |  | 1 | 1 | 2 | 2 |
|  |  |  |  |  |  |  | AMOXYCILLIN+CLAVULANATE | 2 | 1,2 |  | 1 | 12 |  | 1 | 3 | 32 | 32 |
|  |  |  |  |  |  |  | PIPERACILLIN+TAZOBACTAM | 2 | 1,2 |  | 1 | 3 |  | 1 | 3 | 9 | 6 |
| 078/2020 | 6 | 1 | SICKLE CELL, OSTEOMYLITIS, PNEUMONIA | 2 | 2 | 4 | CEFTRIAXONE | 1 | 1,2 |  | 1 | 3 | 3 | 1 | 2 | 6 | 4 |
|  |  |  |  |  |  |  | AMPICILLIN+CLOXACILLIN | 1 | 2 |  | 1 | 3 |  | 1 | 4 | 12 | 12 |
|  |  |  |  |  |  |  | CLINDAMYCIN | 1 | 1,2 |  | 1 | 2 |  | 1 | 3 | 6 | 2 |
| 079/2020 | 2.28 | 2 | MENINGITIS, LOS | 2 | 2 | 5 | CEFOTAXIME | 1 | 1,2 |  | 1 | 3 | 3 | 1 | 3 | 9 | 7 |
|  |  |  |  |  |  |  | AMIKACIN | 1 | 2 |  | 1 | 1 |  | 1 | 1 | 1 | 1 |
| 082/2020 | 6 | 1 | CSD, PSVT | 2 | 2 | 4 | CEFTRIAXONE | 1 | 1,2 |  | 1 | 3 | 3 | 1 | 2 | 6 | 5 |
| 081/2020 | 1.16 | 1 | SEPTIC ARTHRITIS, PATHOLOGICAL # L. HIP, SEPTIC SHOCK | 2 | 2 | 8 | CEFOTAXIME | 1 | 1,2 |  | 1 | 1 | 8 | 1 | 3 | 3 | 3 |
|  |  |  |  |  |  |  | VANCOMYCIN | 1 | 1,2 |  | 1 | 8 |  | 1 | 3 | 24 | 22 |
|  |  |  |  |  |  |  | AZITHROMYCIN | 1 | 1,2 |  | 2 | 4 |  | 1 | 1 | 4 | 4 |
|  |  |  |  |  |  |  | MEROPENEM | 1 | 2 |  | 1 | 4 |  | 1 | 3 | 12 | 12 |
|  |  |  |  |  |  |  | PIPERACILLIN+TAZOBACTAM | 2 | 1,2 |  | 1 | 3 |  | 1 | 3 | 9 | 8 |
|  |  |  |  |  |  |  | RIFAMPICIN | 1 | 1,2 |  | 2 | 5 |  | 1 | 2 | 12 | 12 |
| 082/2020 | 13 | 1 | SCD, TRAUMATIC HEAD INJURY, RAISED ICP | 2 | 2 | 7 | CEFTRIAXONE | 1 | 1,2 |  | 1 | 7 | 7 | 1 | 2 | 14 | 13 |
| 083/2020 | 6 | 1 | SCD, OSTEOMYLITIS, PNEUMONIA | 2 | 2 | 5 | VANCOMYCIN | 1 | 1,2 |  | 1 | 5 | 5 | 1 | 3 | 15 | 14 |
|  |  |  |  |  |  |  | MEROPENEM | 1 | 2 |  | 1 | 5 |  | 1 | 3 | 15 | 14 |
|  |  |  |  |  |  |  | PIPERACILLIN+TAZOBACTAM | 2 | 1,2 |  | 1 | 1 |  | 1 | 3 | 3 | 2 |
| 084/2020 | 2.28 | 1 | ASPIRATIONAL PNEUMONIA, CHARGE SYNDROME | 2 | 2 | 12 | VANCOMYCIN | 1 | 1,2 |  | 1 | 12 | 12 | 1 | 3 | 36 | 32 |
|  |  |  |  |  |  |  | MEROPENEM | 1 | 2 |  | 1 | 12 |  | 1 | 3 | 36 | 32 |
| 085/2020 | 2.66 | 1 | FUC OF LATE PRETERM, RESP FAILURE, POST OP ESOPHAGEAL DUPLICATION CYST, TT TUBE IN SITU, PNEUMONIA | 1,2 | 5 | 6 | CEFOTAXIME | 1 | 1,2 |  | 1 | 6 | 6 | 1 | 3 | 27 | 24 |
|  |  |  |  |  |  |  | AMOXYCILLIN+CLAVULANATE | 2 | 1,2 |  | 1 | 3 |  | 1 | 2 | 6 | 6 |
|  |  |  |  |  |  |  | AMPICILLIN+CLOXACILLIN | 1 | 1,2 |  | 1 | 6 |  | 1 | 4 | 36 | 33 |
| 086/2020 | 8 | 2 | CHD | 2 | 2 | 32 | AMOXYCILLIN+CLAVULANATE | 2 | 1,2 |  | 1 | 6 | 6 | 1 | 2 | 12 | 12 |
| 087/2020 | 7 | 2 | SLE, HEPATITIS A | 2 | 2 | 3 | AMIKACIN | 1 | 2 |  | 1 | 3 | 3 | 1 | 1 | 3 | 1 |
|  |  |  |  |  |  |  | PIPERACILLIN+TAZOBACTAM | 2 | 1,2 |  | 1 | 3 |  | 1 | 3 | 9 | 8 |
| 088/2020 | 2.5 | 1 | ACUTE BRONCHIOLITS, SEVERE RESP DISTRESS | 2 | 2 | 3 | AZITHROMYCIN | 1 | 1,2 |  | 2 | 3 | 3 | 1 | 1 | 3 | 2 |
| 089/2020 | 2.33 | 1 | DOWNS SYNDROME, VSD, PULM HTN, CONGENITAL HYPOTHYROIDISM, FTT, HYPOVITAMINOSIS D, PNEUMONIA, SAM | 2 | 2 | 4 | GENTAMICIN | 1 | 1,2 |  | 1 | 4 | 4 | 1 | 2 | 8 | 8 |
|  |  |  |  |  |  |  | AMPICILLIN | 1 | 1,2 |  | 1 | 4 |  | 1 | 3 | 12 | 12 |
|  |  |  |  |  |  |  | AMOXYCILLIN+CLAVULANATE | 2 | 1,2 |  | 1 | 4 |  | 1 | 2 | 8 | 8 |
| 090/2020 | 13 | 1 | WILSON DISEASE, HEPATIC ENCEPHALOPATHY STAG-3, PORTAL HTN, VARICEAL BLEED | 1,2,3 | 19 | 19 | CEFOTAXIME | 1 | 1,2 |  | 1 | 13 | 13 | 1 | 3 | 39 | 39 |
|  |  |  |  |  |  |  | AMPICILLIN+CLOXACILLIN | 1 | 2 |  | 1 | 11 |  | 1 | 13 | 1 | 13 |
|  |  |  |  |  |  |  | RIFAXIMIN | 1 | 2 |  | 2 | 13 |  | 1 | 2 | 26 | 26 |
|  |  |  |  |  |  |  |  |  |  |  |  |  |  |  |  |  |  |
| 091/2020 | 3 | 1 | HEMOLYTIC ANEMIA, POSTOP SPLENECTOMY, DRAIN IN SITU | 2 | 2 | 3 | CEFTRIAXONE | 1 | 1,2 |  | 1 | 3 | 3 | 1 | 2 | 6 | 5 |
|  |  |  |  |  |  |  | AMIKACIN | 1 | 2 |  | 1 | 3 |  | 1 | 1 | 3 | 3 |
| 092/2020 | 8 | 2 | ENCEPHALITIS | 2 | 2 | 3 | CEFTRIAXONE | 1 | 1,2 |  | 1 | 3 | 3 | 1 | 2 | 6 | 6 |
| 093/2020 | 2.28 | 2 | SEVERE PNEUMONIA, SEPTIC SHOCK, VIRAL ENCEPHALITIS, AKI, B/L PNEUMOTHORAX | 1,2,3 | 7 | 7 | VANCOMYCIN | 1 | 1,2 |  | 1 | 4 | 7 | 1 | 3 | 12 | 12 |
|  |  |  |  |  |  |  | MEROPENEM | 1 | 2 |  | 1 | 5 |  | 1 | 3 | 15 | 12 |
|  |  |  |  |  |  |  | COLISTIN | 1 | 2 |  | 1 | 2 |  | 1 | 3 | 6 | 6 |
| 094/2020 | 13 | 2 | DCMP, SLE, LUPUS NEPHRITIS, HYPOTHYROIDISM, IVH, RAISED ICT | 2 | 2 | 12 | AMIKACIN | 1 | 2 |  | 1 | 5 | 12 | 1 | 1 | 5 | 5 |
|  |  |  |  |  |  |  | AMOXYCILLIN+CLAVULANATE | 2 | 1,2 |  | 1 | 5 |  | 1 | 2 | 12 | 8 |
|  |  |  |  |  |  |  | PIPERACILLIN+TAZOBACTAM | 2 | 1,2 |  | 1 | 5 |  | 1 | 3 | 15 | 15 |
| 095/2020 | 4 | 1 | T CELL ALL, LT SIDED EMPYEMA, RT SIDE CAVITARY LESION, SEVERE ANEMIA | 2 | 2 | 4 | CEFTRIAXONE | 1 | 1,2 |  | 1 | 3 | 4 | 1 | 2 | 6 | 5 |
|  |  |  |  |  |  |  | AMIKACIN | 1 | 2 |  | 1 | 3 |  | 1 | 1 | 3 | 3 |
| 096/2020 | 5 | 2 | RECURRENT NLH, SEIZURE, FEBRILE NEUTROPENIA, DISSEMINATED FUNGAL SEPSIS | 2 | 2 | 5 | VANCOMYCIN | 1 | 1,2 |  | 1 | 2 | 5 | 1 | 3 | 6 | 6 |
|  |  |  |  |  |  |  | COLISTIN | 1 | 2 |  | 1 | 2 |  | 1 | 3 | 6 | 6 |
|  |  |  |  |  |  |  | MEROPENEM | 1 | 1,2 |  | 1 | 5 |  | 1 | 3 | 15 | 15 |
| 097/2020 | 9 | 2 | ACUTE ONSET THROMBOCYTOPENIA | 2 | 2 | 4 | CEFTRIAXONE | 1 | 1,2 |  | 1 | 3 | 4 | 1 | 2 | 6 | 6 |
|  |  |  |  |  |  |  |  |  |  |  |  |  |  |  |  |  |  |
| 098/2020 | 12 | 1 | CHRONIC LUNG DS, PAC, CHRONIC HYPERSENSITIVITY PNEUMONITIS, PULM TB ON ATT | 2 | 2 | 4 | CEFTRIAXONE | 1 | 1,2 |  | 1 | 3 | 4 | 1 | 2 | 6 | 6 |
| 099/2020 | 11 | 1 | PRE-B CELL ALL ON CHEMOTHERAPY | 2 | 2 | 3 | COTRIMOXAZOLE | 2 | 1,2 |  | 1 | 3 | 3 | 1 | 4 | 12 | 12 |
|  |  |  |  |  |  |  | AMIKACIN | 1 | 2 |  | 1 | 3 |  | 1 | 1 | 3 | 3 |
| 100/2020 | 1 | 2 | EPILEPSY, GDD | 2 | 2 | 3 | CEFOTAXIME | 1 | 1,2 |  | 1 | 3 | 3 | 1 | 2 | 6 | 4 |
| 101/2020 | 2.75 | 2 | NEONATAL CHOLESTASIS, CMV HEPATITIS, ACUTE LIVER FAILURE, ENCEPHALITIS | 2 | 2 | 5 | CEFOTAXIME | 1 | 1,2 |  | 1 | 4 | 4 | 1 | 3 | 12 | 11 |
|  |  |  |  |  |  |  | AMPICILLIN | 1 | 1,2 |  | 1 | 4 |  | 1 | 16 | 14 | 19 |
| 102/2020 | 5 | 1 | RT. THIGH CELLULITIS, SEPTIC SHOCK | 2 | 2 | 8 | CEFTRIAXONE | 1 | 1,2 |  | 1 | 7 | 8 | 1 | 2 | 14 | 14 |
|  |  |  |  |  |  |  | VANCOMYCIN | 1 | 1,2 |  | 1 | 7 |  | 1 | 4 | 28 | 28 |
|  |  |  |  |  |  |  | AMIKACIN | 1 | 2 |  | 1 | 6 |  | 1 | 1 | 6 | 6 |
| 103/2020 | 6 | 2 | PULM TB, SEPTIC SHOCK, MODS, PULM HAEMORRHAGE | 1,2,3 | 5 | 5 | CEFTRIAXONE | 1 | 1,2 |  | 1 | 5 | 5 | 1 | 2 | 12 | 12 |
|  |  |  |  |  |  |  | VANCOMYCIN | 1 | 1,2 |  | 1 | 5 |  | 1 | 2 | 12 | 12 |
|  |  |  |  |  |  |  | LINEZOLID | 1 | 1,2 |  | 1 | 3 |  | 1 | 3 | 9 | 9 |
|  |  |  |  |  |  |  | LEVOFLOXACIN | 1 | 1,2 |  | 1 | 3 |  | 1 | 1 | 3 | 3 |
| 104/2020 | 8 | 1 | HYPERTENSIVE EMERGENCY, NEPHROTIC SYNDROME, HYPOCALCEMIC SEIZURE, ENCEPHALOPATHY | 2 | 2 | 5 | CEFTRIAXONE | 1 | 1,2 |  | 1 | 3 | 3 | 1 | 2 | 6 | 6 |
| 105/2020 | 2.4 | 1 | DE GEORGE SYNDROME | 1,2,3 | 4 | 7 | CEFOTAXIME | 1 | 1,2 |  | 1 | 3 | 7 | 1 | 4 | 12 | 13 |
|  |  |  |  |  |  |  | MEROPENEM | 1 | 2 |  | 1 | 3 |  | 1 | 3 | 9 | 8 |
|  |  |  |  |  |  |  | AMIKACIN | 1 | 2 |  | 1 | 3 |  | 1 | 1 | 3 | 3 |
|  |  |  |  |  |  |  | PIPERACILLIN+TAZOBACTAM | 2 | 1,2 |  | 1 | 4 |  | 1 | 3 | 12 | 13 |
| 106/2020 | 2.28 | 2 | LYMPHANGIOMA NECK | 1,2,3 | 7 | 12 | AMIKACIN | 1 | 2 |  | 1 | 9 | 12 | 1 | 1 | 9 | 9 |
|  |  |  |  |  |  |  | PIPERACILLIN+TAZOBACTAM | 2 | 1,2 |  | 1 | 9 |  | 1 | 3 | 27 | 23 |
| 107/2020 | 12 | 1 | ACUTE VIRAL ENCEPHALITIS | 2 | 2 | 4 | CEFTRIAXONE | 1 | 1,2 |  | 1 | 3 | 4 | 1 | 2 | 6 | 3 |
| 108/2020 | 2.5 | 1 | ASD, VSD, SEVERE PS, POSTOP NEPHRECTOMY FOR WILMS TUMOR, FTT | 2 | 2 | 4 | CEFTRIAXONE | 1 | 1,2 |  | 1 | 4 | 4 | 1 | 2 | 8 | 3 |
|  |  |  |  |  |  |  | CEFOTAXIME | 1 | 1,2 |  | 1 | 1 |  | 1 | 3 | 3 | 2 |
|  |  |  |  |  |  |  | AMIKACIN | 1 | 2 |  | 1 | 4 |  | 1 | 1 | 4 | 4 |
| 109/2020 | 2.75 | 1 | TB MENINGITIS, PULMONARY TB, LT HEMIPARESIS, HYDROCEPHALUS, POSTOP VP SHUNT, SAM | 1,2,3 | 11 | 11 | CEFTRIAXONE | 1 | 1,2 |  | 1 | 4 | 11 | 1 | 2 | 8 | 6 |
| 110/2020 | 1 | 1 | AFP, GBS | 2 | 2 | 9 | AMOXYCILLIN+CLAVULANATE | 2 | 1,2 |  | 1 | 1 | 9 | 1 | 2 | 2 | 2 |
|  |  |  |  |  |  |  | AMOXYCILLIN+CLAVULANATE | 2 | 1,2 |  | 2 | 5 |  | 2 | 2 | 12 | 12 |
| 111/2020 | 14 | 1 | B CELL ALL ON DELAYED INTENSIFICATION PHASE | 2 | 2 | 7 | CEFTAZIDIME | 1 | 1,2 |  | 1 | 3 | 7 | 1 | 3 | 9 | 12 |
|  |  |  |  |  |  |  | COTRIMOXAZOLE | 2 | 1,2 |  | 2 | 6 |  | 1 | 4 | 24 | 24 |
|  |  |  |  |  |  |  | AMIKACIN | 1 | 2 |  | 1 | 2 |  | 1 | 1 | 2 | 2 |
|  |  |  |  |  |  |  | AMOXYCILLIN+CLAVULANATE | 2 | 1,2 |  | 2 | 1 |  | 1 | 2 | 2 | 1 |
| 112/2020 | 1 | 2 | SEIZURE DISORDER, GDD | 2 | 2 | 4 | CEFTRIAXONE | 1 | 1,2 |  | 1 | 1 | 4 | 1 | 2 | 2 | 1 |
| 113/2020 | 2.75 | 1 | ACUTE ENCEPHALOPATHY, RAISED ICP | 1,2,3 | 17 | 28 | CEFTRIAXONE | 1 | 1,2 |  | 1 | 1 | 6 | 1 | 2 | 2 | 2 |
|  |  |  |  |  |  |  | VANCOMYCIN | 1 | 1,2 |  | 1 | 5 |  | 1 | 2 | 12 | 12 |
|  |  |  |  |  |  |  | MEROPENEM | 1 | 2 |  | 1 | 5 |  | 1 | 3 | 15 | 15 |
|  |  |  |  |  |  |  | AMIKACIN | 1 | 2 |  | 1 | 1 |  | 1 | 1 | 1 | 1 |
|  |  |  |  |  |  |  | PIPERACILLIN+TAZOBACTAM | 2 | 1,2 |  | 1 | 1 |  | 1 | 3 | 3 | 1 |
| 114/2020 | 2.75 | 1 | PV VALVE FULGRATION, MET. ACIDOSIS, HYPONATREMIA, HYPERKALEMIA, ANEMIA, UTI, CKD | 2 | 2 | 7 | CEFTRIAXONE | 1 | 1,2 |  | 1 | 3 | 4 | 1 | 2 | 6 | 7 |
| 115/2020 | 2.33 | 2 | BRONCHOPNEUMONIA, BRONCHIOLITIS | 1,2,3 | 8 | 8 | CEFOTAXIME | 1 | 1,2 |  | 1 | 2 | 8 | 1 | 3 | 6 | 4 |
|  |  |  |  |  |  |  | VANCOMYCIN | 1 | 1,2 |  | 1 | 7 |  | 1 | 3 | 21 | 22 |
|  |  |  |  |  |  |  | AZITHROMYCIN | 1 | 1,2 |  | 2 | 3 |  | 1 | 1 | 3 | 3 |
|  |  |  |  |  |  |  | MEROPENEM | 1 | 2 |  | 1 | 5 |  | 1 | 3 | 15 | 15 |
|  |  |  |  |  |  |  | AMIKACIN | 1 | 2 |  | 1 | 1 |  | 1 | 1 | 1 | 1 |
|  |  |  |  |  |  |  | GENTAMICIN | 1 | 1,2 |  | 1 | 2 |  | 1 | 1 | 2 | 2 |
|  |  |  |  |  |  |  | PIPERACILLIN+TAZOBACTAM | 2 | 1,2 |  | 1 | 2 |  | 1 | 3 | 6 | 5 |
|  |  |  |  |  |  |  | AMPICILLIN+CLOXACILLIN | 1 | 2 |  | 1 | 4 |  | 1 | 4 | 1 | 6 |
| 116/2020 | 2.28 | 1 | HYDROCEPHALUS WITH VP SHUNT | 2 | 2 | 5 | CEFTRIAXONE | 1 | 1,2 |  | 1 | 3 | 3 | 1 | 2 | 6 | 6 |
| 117/2020 | 13 | 1 | T CELL ALL, HEP-B, R LL PNEUMONIA, FEBRILE NEUTROPENIA | 1,2,3 | 11 | 15 | COTRIMOXAZOLE | 2 | 1,2 |  | 1 | 15 | 15 | 1 | 2 | 34 | 32 |
|  |  |  |  |  |  |  | VANCOMYCIN |  | 1,2 |  | 1 | 15 |  | 1 | 4 | 68 | 65 |
|  |  |  |  |  |  |  | MEROPENEM | 1 | 2 |  | 1 | 15 |  | 1 | 3 | 51 | 49 |
|  |  |  |  |  |  |  | AMIKACIN | 1 | 2 |  | 1 | 1 |  | 1 | 1 | 1 | 1 |
|  |  |  |  |  |  |  | COLISTIN | 1 | 2 |  | 1 | 15 |  | 1 | 3 | 51 | 49 |
| 118/2020 | 13 | 2 | BLEEDIN, ANEMIA | 2 | 2 | 4 | CEFTRIAXONE | 1 | 1,2 |  | 1 | 4 | 4 | 1 | 2 | 8 | 7 |
| 119/2020 | 3 | 1 | RAT POISONING | 1,2,3 | 15 | 19 | CEFOTAXIME | 1 | 1,2 |  | 1 | 8 | 8 | 1 | 3 | 12 | 12 |
| 120/2020 | 2 | 1 | LEUKEMIA | 2 | 2 | 3 | CEFTAZIDIME | 1 | 1,2 |  | 1 | 3 | 3 | 1 | 3 | 9 | 9 |
|  |  |  |  |  |  |  | AMIKACIN | 1 | 2 |  | 1 | 3 |  | 1 | 1 | 3 | 3 |
| 121/2020 | 2.42 | 2 | SEVERE PNEUMONIA | 2 | 2 | 3 | CEFOTAXIME | 1 | 1,2 |  | 1 | 3 | 3 | 1 | 3 | 9 | 7 |
| 122/2020 | 5 | 1 | ENCEPHALOPATHY, STATUS EPILEPTICUS, TRACHEOSTOMY IN SITU | 2 | 2 | 13 | CEFTRIAXONE | 1 | 1,2 |  | 1 | 8 | 13 | 1 | 2 | 16 | 15 |
|  |  |  |  |  |  |  | AMIKACIN | 1 | 2 |  | 1 | 12 |  | 1 | 1 | 12 | 12 |
| 123/2020 | 12 | 2 | T CELL ALL, METASTASIS, FEBRILE NEUTROPENIA, SEPTIC SHOCK | 2 | 2 | 4 | CEFTAZIDIME | 1 | 1,2 |  | 1 | 1 | 4 | 1 | 3 | 3 | 1 |
|  |  |  |  |  |  |  | COTRIMOXAZOLE | 2 | 1,2 |  | 2 | 3 |  | 1 | 1 | 3 | 3 |
|  |  |  |  |  |  |  | VANCOMYCIN | 1 | 1,2 |  | 1 | 2 |  | 1 | 4 | 8 | 8 |
|  |  |  |  |  |  |  | AMIKACIN | 1 | 2 |  | 1 | 2 |  | 1 | 1 | 2 | 2 |
|  |  |  |  |  |  |  | COLISTIN | 1 | 2 |  | 1 | 1 |  | 1 | 3 | 3 | 3 |
|  |  |  |  |  |  |  | PIPERACILLIN+TAZOBACTAM | 2 | 1,2 |  | 1 | 3 |  | 1 | 3 | 9 | 9 |
| 124/2020 | 2.75 | 1 | HEMOPHILIA, POSTOP ILEAL PERFORATION, TRANSIENT INTUSUSCEPTION, SEPTIC SHOCK | 2,3 | 2 | 5 | VANCOMYCIN | 1 | 1,2 |  | 1 | 4 | 4 | 1 | 4 | 16 | 16 |
|  |  |  |  |  |  |  | COLISTIN | 1 | 2 |  | 1 | 4 |  | 1 | 3 | 12 | 12 |
|  |  |  |  |  |  |  | METRONIDAZOLE | 1 | 1,2 |  | 1 | 4 |  | 1 | 3 | 12 | 12 |
|  |  |  |  |  |  |  | CIPROFLOXACIN | 1 | 1,2 |  | 1 | 1 |  | 1 | 2 | 2 | 2 |
| 125/2020 | 2.33 | 1 | HIE, SPASTIC QUADRIPARESIS, FTT, ASPIRATIONAL PNEUMONIA | 2 | 2 | 4 | CEFTRIAXONE | 1 | 1,2 |  | 1 | 4 | 4 | 1 | 2 | 8 | 8 |
|  |  |  |  |  |  |  | METRONIDAZOLE | 1 | 1,2 |  | 1 | 4 |  | 1 | 3 | 12 | 12 |
| 126/2020 | 7 | 1 | PERICARDIAL EFFUSION, PLEURAL EFFUSION, HBSAG | 2 | 2 | 8 | CEFTRIAXONE | 1 | 1,2 |  | 1 | 8 | 8 | 1 | 2 | 16 | 16 |
|  |  |  |  |  |  |  | VANCOMYCIN | 1 | 1,2 |  | 1 | 8 |  | 1 | 4 | 32 | 32 |
|  |  |  |  |  |  |  | CLINDAMYCIN | 1 | 1,2 |  | 1 | 6 |  | 1 | 3 | 18 | 17 |
| 127/2020 | 12 | 1 | SNAKE BITE | 1,2,3 | 4 | 5 | CEFTRIAXONE | 1 | 1,2 |  | 1 | 5 | 5 | 1 | 2 | 12 | 9 |
|  |  |  |  |  |  |  | AMOXYCILLIN+CLAVULANATE | 2 | 1,2 |  | 1 | 1 |  | 1 | 2 | 2 | 1 |
| 128/2020 | 8 | 1 | B/L KNEE JOINT SEPTIC ARTHRITIS, SEPTIC SHOCK, MODS | 2 | 2 | 5 | VANCOMYCIN | 1 | 1,2 |  | 1 | 5 | 5 | 1 | 3 | 15 | 15 |
|  |  |  |  |  |  |  | MEROPENEM | 1 | 2 |  | 1 | 5 |  | 1 | 3 | 15 | 15 |
|  |  |  |  |  |  |  | CLINDAMYCIN | 1 | 1,2 | 1 | 1 | 5 |  | 1 | 3 | 15 | 15 |
| 129/2020 | 2.41 | 2 | BIRTH ASPHYXIA, HIE, EPILEPSY | 2 | 2 | 4 | CEFTRIAXONE | 1 | 1,2 | 2 | 1 | 2 | 4 | 1 | 2 | 4 | 4 |
|  |  |  |  |  |  |  | GENTAMICIN | 1 | 1,2 | 1 | 1 | 1 |  | 1 | 1 | 1 | 1 |
| 130/2020 | 9 | 1 | CEREBRAL MALARIA, SCRUB TYPHUS, LEPTOSPIROSIS, SEPTIC SHOCK, MYOCARDITIS, AEDS | 2 | 2 | 7 | CEFTRIAXONE | 1 | 1,2 | 2 | 1 | 7 | 7 | 1 | 2 | 14 | 13 |
|  |  |  |  |  |  |  | VANCOMYCIN | 1 | 1,2 | 2 | 1 | 4 |  |  | 4 | 16 | 15 |
|  |  |  |  |  |  |  | DOXYCYCLINE | 1 | 1,2 | 1 | 2 | 7 |  |  | 2 | 14 | 14 |
|  |  |  |  |  |  |  | AMPICILLIN | 1 | 1,2 | 1 | 1 | 1 |  |  | 4 | 4 | 2 |
| 131/2020 | 2 | 2 | SAM, MODERATE DEHYDRATION, TSS | 2 | 2 | 5 | AMOXYCILLIN+CLAVULANATE | 2 | 1,2 | 1 | 2 | 5 | 5 | 1 | 2 | 12 | 8 |
|  |  |  |  |  |  |  | CLINDAMYCIN | 1 | 1,2 | 1 | 1 | 5 |  | 1 | 3 | 15 | 12 |
| 132/2020 | 12 | 1 | NEPHROTIC SYNDROME, SBP, GM -VE SEPSIS, HTN | 2 | 2 | 7 | COLISTIN | 1 | 2 | 3 | 1 | 7 | 7 | 1 | 3 | 21 | 22 |
| 133/2020 | 2.5 | 1 | MIDLINE CLEFT LIP, PALATE, SEPSIS, PNEUMONIA | 1,2,3 | 12 | 17 | VANCOMYCIN | 1 | 1,2 | 2 | 1 | 13 | 17 | 1 | 3 | 39 | 37 |
|  |  |  |  |  |  |  | MEROPENEM | 1 | 2 | 2 | 1 | 4 |  | 1 | 3 | 12 | 11 |
|  |  |  |  |  |  |  | PIPERACILLIN+TAZOBACTAM | 2 | 1,2 | 2 | 1 | 17 |  | 1 | 3 | 51 | 49 |
| 134/2020 | 2.16 | 2 | PNEUMONIA, RESP FAILURE, MICROCEPHALY, TB ON ATT | 1,2,3 | 3 | 4 | CEFTRIAXONE | 1 | 1,2 | 2 | 1 | 3 | 4 | 1 | 2 | 6 | 5 |
|  |  |  |  |  |  |  | VANCOMYCIN | 1 | 1,2 | 2 | 1 | 3 |  | 1 | 4 | 12 | 12 |
| 135/2020 | 2.16 | 2 | LOS, PNEUMONIA, RESP FAILURE, FT, PULM TB ON ATT | 2 | 2 | 4 | CEFTRIAXONE | 1 | 1,2 | 2 | 1 | 5 | 4 | 1 | 2 | 12 | 9 |
|  |  |  |  |  |  |  | VANCOMYCIN | 1 | 1,2 | 2 | 1 | 4 |  | 1 | 4 | 16 | 14 |
| 136/2020 | 4 | 2 | CRUSH INJURY LT U.P. GANGRENE, POST OP AMPUTATION B.E. | 2 | 2 | 3 | CEFTRIAXONE | 1 | 1,2 | 2 | 1 | 3 | 3 | 1 | 2 | 6 | 5 |
| 137/2020 | 2.28 | 2 | FT, MAS, HIE-2, SEIZURE, ICH, SEPSIS | 1,2,3 | 12 | 25 | VANCOMYCIN | 1 | 1,2 | 2 | 1 | 21 | 25 | 1 | 3 | 63 | 61 |
|  |  |  |  |  |  |  | MEROPENEM | 1 | 2 | 2 | 1 | 14 |  | 1 | 3 | 42 | 39 |
|  |  |  |  |  |  |  | AMIKACIN | 1 | 2 | 1 | 1 | 1 |  | 1 | 1 | 1 | 1 |
|  |  |  |  |  |  |  | COLISTIN | 1 | 2 | 3 | 1 | 12 |  | 1 | 3 | 32 | 29 |
|  |  |  |  |  |  |  | PIPERACILLIN+TAZOBACTAM | 2 | 1,2 | 2 | 1 | 3 |  | 1 | 3 | 9 | 7 |
| 138/2020 | 5 | 1 | NEONATAL HEMORRHAGE, ENCEPHALITIS, MICROCEPHALY, GDD, COMPLICATED SAM | 2 | 2 | 9 | CEFTRIAXONE | 1 | 1,2 | 2 | 1 | 6 | 9 | 1 | 2 | 12 | 12 |
|  |  |  |  |  |  |  | AMIKACIN | 1 | 2 | 1 | 1 | 3 |  | 1 | 1 | 3 | 3 |
|  |  |  |  |  |  |  | AMPICILLIN+CLOXACILLIN | 1 | 2 | 1 | 1 | 6 |  | 1 | 4 | 24 | 24 |
| 139/2020 | 9 | 1 | NEPHROTIC SYNDROME | 1,2,3 | 8 | 12 | CEFTRIAXONE | 1 | 1,2 | 2 | 1 | 1 | 12 | 1 | 2 | 2 | 1 |
|  |  |  |  |  |  |  | CEFIXIME | 1 | 1,2 | 2 | 2 | 2 |  | 1 | 2 | 4 | 2 |
|  |  |  |  |  |  |  | AMIKACIN | 1 | 2 | 1 | 1 | 12 |  | 1 | 1 | 12 | 12 |
|  |  |  |  |  |  |  | PIPERACILLIN+TAZOBACTAM | 2 | 1,2 | 2 | 1 | 12 |  | 1 | 3 | 36 | 34 |
| 140/2020 | 2.28 | 2 | CHD, ASD, ASPIRATIONAL PNEUMONIA, SEVERE POST CARDIAC ARREST | 1,2,3 | 12 | 11 | CEFOTAXIME | 1 | 1,2 | 2 | 1 | 2 | 11 | 1 | 3 | 6 | 3 |
|  |  |  |  |  |  |  | VANCOMYCIN | 1 | 1,2 | 2 | 1 | 9 |  | 1 | 3 | 27 | 27 |
|  |  |  |  |  |  |  | AMIKACIN | 1 | 2 | 1 | 1 | 2 |  | 1 | 1 | 2 | 2 |
|  |  |  |  |  |  |  | PIPERACILLIN+TAZOBACTAM | 2 | 1,2 | 2 | 1 | 9 |  | 1 | 3 | 27 | 26 |
| 141/2020 | 2.16 | 1 | AGA, MENINGITIS, HYDROCEPHALUS, AQUEDUCTAL STENOSIS, CMV IGN +VS, SHOCK | 1,2 | 11 | 11 | VANCOMYCIN | 1 | 1,2 | 2 | 1 | 11 | 11 | 1 | 2 | 22 | 22 |
| 142/2020 | 8 | 1 | AES, STATUS EPILEPTICUS, PALLOR, SCRUB TYPHUS, RAISED ICP | 2 | 2 |  | MEROPENEM | 1 | 2 | 2 | 1 | 11 |  | 1 | 3 | 33 | 33 |
| 143/2020 | 12 | 1 | CKD | 2 | 2 | 3 | CEFTRIAXONE | 1 | 1,2 | 2 | 1 | 3 | 3 | 1 | 2 | 6 | 6 |
|  |  |  |  |  |  |  | AZITHROMYCIN | 1 | 1,2 | 2 | 1 | 3 |  | 1 | 1 | 3 | 3 |
| 144/2020 | 12 | 1 | NEUROBLASTOMA | 2 | 2 | 14 | CEFTRIAXONE | 1 | 1,2 | 2 | 1 | 14 | 14 | 1 | 2 | 28 | 26 |
| 145/2020 | 9 | 2 | MENINGOENCEPHALITIS, RAISED ICP | 2 | 2 | 11 | COTRIMOXAZOLE | 2 | 1,2 | 1 | 2 | 12 | 12 | 1 | 1 | 12 | 12 |
| 146/2020 | 3 | 2 | NEPHROTOC SYNDROME, AKI | 2 | 2 | 4 | CEFTRIAXONE | 1 | 1,2 | 2 | 1 | 4 | 4 | 1 | 2 | 8 | 7 |
| 147/2020 | 8 | 2 | STATUS ASTHMATICUS | 1,2,3 | 4 | 9 | PIPERACILLIN+TAZOBACTAM | 1 | 1,2 | 2 | 1 | 9 | 9 | 1 | 3 | 27 | 27 |
| 148/2020 | 5 | 1 | STEROID RESISTANT NEPHROTIC SYNDROME | 2 | 2 | 4 | CEFTRIAXONE | 1 | 1,2 | 2 | 1 | 3 | 4 | 1 | 2 | 6 | 6 |
|  |  |  |  |  |  |  | VANCOMYCIN | 1 | 1,2 | 2 | 1 | 3 |  | 1 | 3 | 15 | 15 |
|  |  |  |  |  |  |  | AZITHROMYCIN | 1 | 1,2 | 2 | 2 | 3 |  | 1 | 1 | 3 | 3 |
|  |  |  |  |  |  |  | MEROPENEM | 1 | 2 | 2 | 1 | 3 |  | 1 | 3 | 9 | 9 |
|  |  |  |  |  |  |  | PIPERACILLIN+TAZOBACTAM | 2 | 1,2 | 2 | 1 | 3 |  | 1 | 3 | 9 | 9 |
| 149/2020 | 2 | 1 | ABDOMINAL MASS UE, SEVERE ANEMIA, R PLEURAL EFFUSION | 2 | 2 | 8 | CEFTRIAXONE | 1 | 1,2 | 2 | 1 | 8 | 8 | 1 | 2 | 16 | 14 |
|  |  |  |  |  |  |  | AMOXYCILLIN+CLAVULANATE | 1 | 1,2 | 1 | 1 | 1 |  | 1 | 2 | 2 | 1 |
| 150/2020 | 2 | 1 | TSS, SEPTIC SHOCK, R. LUNG PNEUMONIA | 2 | 2 | 16 | CEFTRIAXONE | 1 | 1,2 | 2 | 1 | 12 | 16 | 1 | 2 | 24 | 23 |
|  |  |  |  |  |  |  | COTRIMOXAZOLE | 2 | 1,2 | 1 | 2 | 6 |  | 1 | 5 | 3 | 3 |
| 151/2020 | 13 | 1 | NHL, GENERALISED LYMPHADENOPATHY | 2 | 2 | 6 | VANCOMYCIN | 1 | 1,2 | 2 | 1 | 5 | 6 | 1 | 3 | 15 | 15 |
|  |  |  |  |  |  |  | MEROPENEM | 1 | 2 | 2 | 1 | 5 |  | 1 | 3 | 15 | 14 |
|  |  |  |  |  |  |  | COLISTIN | 1 | 2 | 3 | 1 | 5 |  | 1 | 3 | 15 | 14 |
| 152/2020 | 9 | 2 | RTA | 2 | 2 | 4 | CEFTRIAXONE | 1 | 1,2 | 2 | 1 | 2 | 4 | 1 | 2 | 4 | 4 |
|  |  |  |  |  |  |  | TEICOPLANIN | 1 | 2 | 2 | 1 | 2 |  | 1 | 2 | 4 | 2 |
|  |  |  |  |  |  |  | PIPERACILLIN+TAZOBACTAM | 2 | 1,2 | 2 | 1 | 2 |  | 1 | 3 | 6 | 5 |
| 153/2020 | 1 | 2 | NEPHROTIC SYNDROME | 1,2,3 | 14 | 4 | PIPERACILLIN+TAZOBACTAM | 2 | 1,2 | 2 | 1 | 3 | 4 | 1 | 3 | 9 | 9 |
|  |  |  |  |  |  |  | METRONIDAZOLE | 1 | 1,2 | 1 | 1 | 3 |  | 1 | 3 | 9 | 9 |
| 154/2020 | 2 | 2 | HTN, PHEOCHROMOCYTOMA, SMALL MUSCULAR VSD | 2 | 2 | 18 | VANCOMYCIN | 1 | 1,2 | 2 | 1 | 5 | 18 | 1 | 3 | 15 | 14 |
|  |  |  |  |  |  |  | MEROPENEM | 1 | 2 | 2 | 1 | 7 |  | 1 | 3 | 21 | 22 |
|  |  |  |  |  |  |  | TIGECYCLINE | 1 | 2 | 3 | 1 | 14 |  | 1 | 2 | 28 | 27 |
|  |  |  |  |  |  |  | PIPERACILLIN+TAZOBACTAM | 2 | 1,2 | 2 | 1 | 6 |  | 1 | 3 | 18 | 18 |
| 155/2020 | 1 | 1 | WILMS TUMOR | 1,2,3 | 4 | 3 | CEFTRIAXONE | 1 | 1,2 | 2 | 1 | 3 | 3 | 1 | 2 | 6 | 5 |
| 156/2020 | 2.16 | 2 | MENINGITIS, SEVERE DEHYDRATION, SHOCK | 2 | 2 | 4 | CEFOTAXIME | 1 | 1,2 | 2 | 1 | 2 | 4 | 1 | 3 | 6 | 2 |
|  |  |  |  |  |  |  | CEFTAZIDIME | 1 | 1,2 | 2 | 1 | 4 |  | 1 | 3 | 12 | 11 |
| 156/2020 | 2.16 | 2 | MENINGITIS, SEVERE DEHYDRATION, SHOCK | 2 | 2 | 7 | VANCOMYCIN | 1 | 1,2 | 2 | 1 | 6 | 7 | 1 | 4 | 24 | 22 |
|  |  |  |  |  |  |  | PIPERACILLIN+TAZOBACTAM | 2 | 1,2 | 2 | 1 | 6 |  | 1 | 3 | 18 | 16 |
| 157/2020 | 1 | 2 | NEPHROTIC SYNDROME, SEPTIC SHOCK | 2,3 | 2 | 25 | VANCOMYCIN | 1 | 1,2 | 2 | 1 | 22 | 25 | 1 | 4 | 82 | 76 |
|  |  |  |  |  |  |  | MEROPENEM | 1 | 2 | 2 | 1 | 9 |  | 1 | 2 | 18 | 16 |
|  |  |  |  |  |  |  | TIGECYCLINE | 1 | 2 | 3 | 1 | 7 |  | 1 | 2 | 14 | 14 |
|  |  |  |  |  |  |  | PIPERACILLIN+TAZOBACTAM | 2 | 1,2 | 2 | 1 | 7 |  | 1 | 3 | 21 | 18 |
|  |  |  |  |  |  |  | NITROFURANTOIN | 1 | 1,2 | 1 | 2 | 12 |  | 1 | 4 | 42 | 38 |
| 158/2020 | 2 | 1 | SCRUB TYPHUS, MYOCARDITIS, SEPTIC SHOCK, ARDS | 2 | 2 | 9 | VANCOMYCIN | 1 | 1,2 | 2 | 1 | 7 | 9 | 1 | 2 | 14 | 13 |
|  |  |  |  |  |  |  | AZITHROMYCIN | 1 | 1,2 | 2 | 1 | 9 |  | 1 | 1 | 11 | 11 |
|  |  |  |  |  |  |  | AMIKACIN | 1 | 2 | 1 | 1 | 2 |  | 1 | 1 | 2 | 2 |
|  |  |  |  |  |  |  | PIPERACILLIN+TAZOBACTAM | 2 | 1,2 | 2 | 1 | 8 |  | 1 | 3 | 24 | 23 |
| 159/2020 | 2.28 | 1 | GLUTARIC ACIDURIA TYPE-2 | 2 | 2 | 12 | AMIKACIN | 1 | 2 | 1 | 1 | 8 | 12 | 1 | 1 | 8 | 8 |
|  |  |  |  |  |  |  | PIPERACILLIN+TAZOBACTAM | 2 | 1,2 | 2 | 1 | 12 |  | 1 | 3 | 36 | 35 |
| 160/2020 | 4 | 1 | FTT, CHF, SAM, SEPSIS | 1,2,3 | 6 | 7 | CEFOTAXIME | 1 | 1,2 | 2 | 1 | 1 | 7 | 1 | 3 | 3 | 1 |
|  |  |  |  |  |  |  | AMIKACIN | 1 | 2 | 1 | 1 | 1 |  | 1 | 1 | 1 | 1 |
|  |  |  |  |  |  |  | PIPERACILLIN+TAZOBACTAM | 2 | 1,2 | 2 | 1 | 7 |  | 1 | 3 | 21 | 21 |
| 161/2020 | 2.25 | 2 | P/O VP SHUNT RT. CONGENITAL OBSTRUCTED HYDROCEPHALUS | 2 | 2 | 4 | CEFTRIAXONE | 1 | 1,2 | 2 | 1 | 4 | 4 | 1 | 2 | 8 | 7 |
| 162/2020 | 3 | 1 | NEPHROTIC SYNDROME, AKI, HTN, HYPOVOLEMIC SHOCK, RESP FAILURE | 2 | 2 | 52 | CEFTRIAXONE | 1 | 1,2 | 2 | 1 | 9 | 52 | 1 | 2 | 18 | 16 |
|  |  |  |  |  |  |  | VANCOMYCIN | 1 | 1,2 | 2 | 1 | 1 |  | 1 | 2 | 2 | 1 |
|  |  |  |  |  |  |  | TEICOPLANIN | 1 | 2 | 2 | 1 | 36 |  | 1 | 2 | 72 | 72 |
|  |  |  |  |  |  |  | MEROPENEM | 1 | 2 | 2 | 1 | 28 |  | 1 | 2 | 56 | 56 |
|  |  |  |  |  |  |  | GENTAMICIN | 1 | 1,2 | 1 | 1 | 9 |  | 1 | 1 | 9 | 9 |
|  |  |  |  |  |  |  | COLISTIN | 1 | 2 | 3 | 1 | 32 |  | 1 | 4 | 128 | 124 |
| 163/2020 | 2.66 | 2 | P/O LAPAROTOMY, DUODENAL WEB, PATRTIAL OBSTRUCTION, DOWNS SYNDROME | 2 | 2 | 3 | CEFTRIAXONE | 1 | 1,2 | 2 | 1 | 4 |  | 1 | 2 | 8 | 7 |
|  |  |  |  |  |  |  | AMIKACIN | 1 | 2 | 1 | 1 | 4 |  | 1 | 1 | 4 | 4 |
|  |  |  |  |  |  |  | METRONIDAZOLE | 1 | 1,2 | 1 | 1 | 4 |  | 1 | 3 | 12 | 12 |
| 164/2020 | 12 | 2 | SHOCK, SCRUB TYPHUS | 2 | 2 | 6 | CEFOTAXIME | 1 | 1,2 | 2 | 1 | 6 | 6 | 1 | 3 | 18 | 18 |
|  |  |  |  |  |  |  | VANCOMYCIN | 1 | 1,2 | 2 | 1 | 1 |  | 1 | 4 | 4 | 2 |
|  |  |  |  |  |  |  | AZITHROMYCIN | 1 | 1,2 | 2 | 1 | 6 |  | 1 | 1 | 6 | 6 |
| 165/2020 | 12 | 1 | EWING SARCOMA, BM METASTASIS, FEBRILE NEUTROPENIA | 2 | 2 | 12 | CEFTRIAXONE | 1 | 1,2 | 2 | 1 | 12 | 12 | 1 | 2 | 24 | 24 |
|  |  |  |  |  |  |  | AMPICILLIN+CLOXACILLIN | 1 | 1,2 | 1 | 1 | 12 |  | 1 | 4 | 48 | 48 |
| 166/2020 | 5 | 1 | NEPHROTIC SYNDROME, HTN, FSGS, NPHS2 MUTATION +VE | 2 | 2 | 6 | PIPERACILLIN+TAZOBACTAM | 2 | 1,2 | 2 | 1 | 6 | 6 | 1 | 3 | 18 | 17 |
| 167/2020 | 12 | 2 | GBS, DVT, RT KNEE SEPTIC ARTHRITIS, BL PNEUMOTHORAX, MAM | 2 | 2 | 7 | CEFTRIAXONE | 1 | 1,2 | 2 | 1 | 5 | 7 | 1 | 2 | 12 | 12 |
|  |  |  |  |  |  |  | VANCOMYCIN | 1 | 1,2 | 2 | 1 | 4 |  | 1 | 4 | 16 | 11 |
|  |  |  |  |  |  |  | PIPERACILLIN+TAZOBACTAM | 2 | 1,2 | 2 | 1 | 4 |  | 1 | 3 | 12 | 9 |
| 168/2020 | 1 | 2 | STEROID RESISTANT NEPHROTIC SYNDROME, AKI, HTN | 2 | 2 | 8 | AMOXYCILLIN+CLAVULANATE | 2 | 1,2 | 1 | 2 | 3 | 3 | 1 | 3 | 9 | 7 |
| 169/2020 | 1 | 1 | SAM, PNEUMONIA, MENINGITIS | 2 | 2 | 3 | CEFOTAXIME | 1 | 1,2 | 2 | 1 | 1 | 3 | 1 | 3 | 3 | 2 |
|  |  |  |  |  |  |  | AMIKACIN | 1 | 2 | 1 | 1 | 1 |  | 1 | 1 | 1 | 1 |
|  |  |  |  |  |  |  | AMOXYCILLIN+CLAVULANATE | 2 | 1,2 | 1 | 1 | 3 |  | 1 | 3 | 9 | 6 |
| 170/2020 | 2.33 | 2 | CONGENITAL HYDROCEPHALUS, MALFUNCTIONING VP SHUNT | 2 | 2 | 3 | CEFTRIAXONE | 1 | 1,2 | 2 | 1 | 2 | 2 | 1 | 2 | 4 | 3 |
| 171/2020 | 12 | 2 | SLE, ACUTE IMMUNOENCEPHALITIS | 2 | 2 | 3 | AZITHROMYCIN | 1 | 1,2 | 2 | 1 | 2 | 3 | 1 | 1 | 2 | 2 |
| 172/2020 | 1 | 2 | REMOTE SYMPTOMATIC EPILEPSY, MICROCEPHALY | 2 | 2 | 23 | CEFTRIAXONE | 1 | 1,2 | 2 | 1 | 16 | 23 | 1 | 2 | 32 | 31 |
|  |  |  |  |  |  |  | AMIKACIN | 1 | 2 | 1 | 1 | 8 |  | 1 | 1 | 8 | 8 |
|  |  |  |  |  |  |  | PIPERACILLIN+TAZOBACTAM | 2 | 1,2 | 2 | 1 | 8 |  | 1 | 4 | 32 | 32 |
| 173/2020 | 2.16 | 2 | SEIZURED/O, FTT, PNEUMONIA | 1,2,3 | 17 | 17 | VANCOMYCIN | 1 | 1,2 | 2 | 1 | 9 | 17 | 1 | 4 | 36 | 36 |
|  |  |  |  |  |  |  | MEROPENEM | 1 | 2 | 2 | 1 | 9 |  | 1 | 3 | 27 | 26 |
|  |  |  |  |  |  |  | PIPERACILLIN+TAZOBACTAM | 2 | 1,2 | 2 | 1 | 8 |  | 1 | 3 | 24 | 23 |
| 174/2020 | 8 | 2 | JUVENILE SLE, LUPUS NEPHRITIS | 1,2,3 | 8 | 8 | TEICOPLANIN | 1 | 2 | 2 | 1 | 4 | 8 | 1 | 2 | 8 | 7 |
|  |  |  |  |  |  |  | LINEZOLID | 1 | 1,2 | 3 | 1 | 4 |  | 1 | 3 | 12 | 11 |
|  |  |  |  |  |  |  | PIPERACILLIN+TAZOBACTAM | 2 | 1,2 | 2 | 1 | 4 |  | 1 | 3 | 12 | 11 |
| 175/2020 | 2.28 | 1 | CHD, LATE ONSET SEPSIS, PNEUMONIA | 2 | 2 | 8 | VANCOMYCIN | 1 | 1,2 | 2 | 1 | 7 | 8 | 1 | 4 | 28 | 25 |
|  |  |  |  |  |  |  | MEROPENEM | 1 | 1,2 | 2 | 1 | 2 |  | 1 | 2 | 4 | 3 |
|  |  |  |  |  |  |  | PIPERACILLIN+TAZOBACTAM | 2 | 1,2 | 2 | 1 | 6 |  | 1 | 3 | 18 | 15 |
| 176/2020 | 9 | 2 | ACUTE SPASTIC PARALYSIS, BULBAR PALSY | 2 | 2 | 8 | CEFTRIAXONE | 1 | 1,2 | 2 | 1 | 8 | 8 | 1 | 2 | 16 | 15 |
| 177/2020 | 12 | 1 | EWING SARCOMA, BM METASTASIS, FEBRILE NEUTROPENIA | 2,3 | 2 | 17 | COTRIMOXAZOLE | 2 | 1,2 | 1 | 2 | 17 | 17 | 1 | 5 | 8 | 8 |
|  |  |  |  |  |  |  | VANCOMYCIN | 1 | 1,2 | 2 | 1 | 9 |  | 1 | 4 | 36 | 34 |
|  |  |  |  |  |  |  | TIGECYCLINE | 1 | 2 | 3 | 1 | 11 |  | 1 | 1 | 11 | 11 |
|  |  |  |  |  |  |  | COLISTIN | 1 | 2 | 3 | 1 | 12 |  | 1 | 3 | 36 | 34 |
|  |  |  |  |  |  |  | AMPICILLIN | 1 | 1,2 | 1 | 1 | 9 |  | 1 | 4 | 36 | 34 |
|  |  |  |  |  |  |  | FOSFOMYCIN | 1 | 2 | 3 | 1 | 9 |  | 1 | 4 | 36 | 34 |
| 178/2020 | 2.28 | 1 | CHD, LATE ONSET SEPSIS, PNEUMONIA | 1,2,3 | 3 | 3 | VANCOMYCIN | 1 | 1,2 | 2 | 1 | 3 | 3 | 1 | 1 | 3 | 3 |
|  |  |  |  |  |  |  | MEROPENEM | 1 | 2 | 2 | 1 | 3 |  | 1 | 2 | 6 | 5 |
| 179/2221 | 2.33 | 2 | FTT, CHF | 1,2,3 | 4 | 4 | VANCOMYCIN | 1 | 1,2 | 2 | 1 | 4 | 4 | 1 | 4 | 16 | 13 |
|  |  |  |  |  |  |  | MEROPENEM | 1 | 2 | 2 | 1 | 4 |  | 1 | 3 | 12 | 12 |
| 180/2221 | 2.33 | 2 | CHD, PNEUMONIA, CCF | 1,2,3 | 5 | 32 | CEFTRIAXONE | 1 | 1,2 | 2 | 1 | 4 | 32 | 1 | 2 | 8 | 8 |
|  |  |  |  |  |  |  | COTRIMOXAZOLE | 2 | 1,2 | 1 | 2 | 8 |  | 1 | 5 | 4 | 4 |
|  |  |  |  |  |  |  | VANCOMYCIN | 1 | 1,2 | 2 | 1 | 8 |  | 1 | 4 | 32 | 28 |
|  |  |  |  |  |  |  | AZITHROMYCIN | 1 | 1,2 | 2 | 2 | 6 |  | 1 | 1 | 6 | 6 |
|  |  |  |  |  |  |  | MEROPENEM | 1 | 2 | 2 | 1 | 8 |  | 1 | 3 | 24 | 21 |
|  |  |  |  |  |  |  | TIGECYCLINE | 1 | 2 | 3 | 1 | 4 |  | 1 | 1 | 4 | 4 |
|  |  |  |  |  |  |  | AMIKACIN | 1 | 2 | 1 | 1 | 6 |  | 1 | 1 | 6 | 6 |
|  |  |  |  |  |  |  | GENTAMICIN | 1 | 1,2 | 1 | 1 | 4 |  | 1 | 3 | 12 | 8 |
|  |  |  |  |  |  |  | COLISTIN | 1 | 2 | 3 | 1 | 21 |  | 1 | 2 | 42 | 41 |
|  |  |  |  |  |  |  | LINEZOLID | 1 | 1,2 | 3 | 1 | 17 |  | 1 | 3 | 51 | 46 |
|  |  |  |  |  |  |  | PIPERACILLIN+TAZOBACTAM | 2 | 1,2 | 2 | 1 | 3 |  | 1 | 4 | 12 | 6 |
|  |  |  |  |  |  |  | METRONIDAZOLE | 1 | 1,2 | 1 | 1 | 4 |  | 1 | 3 | 12 | 12 |
| 181/2221 | 2.16 | 2 | PULMONARY ATRESIA, PDA, MAPCA, PNEUMONIA | 2 | 2 | 12 | CEFTRIAXONE | 1 | 1,2 | 1 | 11 | 11 | 1 | 2 | 11 | 12 | 11 |
| 182/2221 | 12 | 1 | ACHD, CCF, INFECTIVE ENDOCARDITIS, PAH | 2 | 2 | 8 | CEFTRIAXONE | 1 | 1,2 | 1 | 8 | 8 | 1 | 2 | 16 | 16 | 8 |
|  |  |  |  |  |  |  | VANCOMYCIN | 1 | 1,2 | 1 | 8 |  | 1 | 3 | 24 | 23 | 8 |
|  |  |  |  |  |  |  | GENTAMICIN | 1 | 1,2 | 1 | 8 |  | 1 | 1 | 8 | 8 | 8 |
| 183/2221 | 2.33 | 1 | TEF, ASPIRATIONAL PNEUMONITIS, T1 RESP FAILURE, FTT | 2 | 2 | 5 | AMIKACIN | 1 | 2 | 1 | 4 | 5 | 1 | 1 | 4 | 5 | 4 |
|  |  |  |  |  |  |  | AMPICILLIN | 1 | 1,2 | 1 | 1 |  | 1 | 4 | 4 | 2 | 1 |
|  |  |  |  |  |  |  | PIPERACILLIN+TAZOBACTAM | 2 | 1,2 | 1 | 4 |  | 1 | 3 | 12 | 13 | 4 |
| 184/2221 | 11 | 2 | MENINGOENCEPHALITIS | 2 | 2 | 19 | VANCOMYCIN | 1 | 1,2 | 1 | 9 | 19 | 1 | 4 | 36 | 36 | 9 |
|  |  |  |  |  |  |  | MEROPENEM | 1 | 2 | 1 | 17 |  | 1 | 3 | 51 | 49 | 17 |
|  |  |  |  |  |  |  | COLISTIN | 1 | 2 | 1 | 6 |  | 1 | 3 | 18 | 18 | 6 |
|  |  |  |  |  |  |  | LINEZOLID | 1 | 1,2 | 1 | 9 |  | 1 | 2 | 18 | 18 | 9 |
| 185/2221 | 2 | 1 | HEPATOBLASTOMA SEG 2 &3, POST CHEMOTHERAPY | 2 | 2 | 5 | CEFOTAXIME | 1 | 1,2 | 1 | 5 | 5 | 1 | 3 | 15 | 11 | 5 |
|  |  |  |  |  |  |  | AMIKACIN | 1 | 2 | 1 | 5 |  | 1 | 1 | 5 | 5 | 5 |
| 186/2221 | 4 | 1 | MENINGITIS, STATUS EPILEPTICUS,LT. LEG DVT | 2 | 2 | 6 | CEFOTAXIME | 1 | 1,2 | 1 | 5 | 6 | 1 | 3 | 15 | 13 | 5 |
|  |  |  |  |  |  |  | AMIKACIN | 1 | 2 | 1 | 5 |  | 1 | 1 | 5 | 5 | 5 |
| 187/2221 | 2.8 | 1 | ANAPLASTIC EPENDYMOMA, AGE,SEVERE DEHYDRATION,NASAL BLEED, PANCYTOPENIA | 1,2,3 | 4 | 4 | CEFTAZIDIME | 1 | 1,2 | 1 | 1 | 4 | 1 | 3 | 2 | 1 | 1 |
|  |  |  |  |  |  |  | VANCOMYCIN | 1 | 1,2 | 1 | 2 |  | 1 | 4 | 8 | 6 | 2 |
|  |  |  |  |  |  |  | MEROPENEM | 1 | 2 | 1 | 2 |  | 1 | 3 | 6 | 4 | 2 |
|  |  |  |  |  |  |  | AMIKACIN | 1 | 2 | 1 | 1 |  | 1 | 1 | 1 | 1 | 1 |
|  |  |  |  |  |  |  | PIPERACILLIN+TAZOBACTAM | 2 | 1,2 | 1 | 3 |  | 1 | 2 | 6 | 7 | 3 |
| 188/2221 | 12 | 2 | ACUTE ENCEPHALITIS, AKI, PULMONARY EDEMA | 2 | 2 | 8 | MEROPENEM | 1 | 2 | 1 | 7 | 7 | 1 | 1 | 7 | 7 | 7 |
|  |  |  |  |  |  |  | LINEZOLID | 1 | 1,2 | 1 | 7 |  | 1 | 3 | 21 | 21 | 7 |
| 189/2221 | 4 | 2 | SLE, LUPUS MEPHRITIS, HTN URGENCY | 2 | 2 | 2 | PIPERACILLIN+TAZOBACTAM | 2 | 1,2 | 1 | 4 | 4 | 1 | 3 | 12 | 12 | 4 |
| 190/2221 | 7 | 1 | T CELL ALL, ICH, RT HEMIPARESIS, POSTOP CRANIOTOMY, EVACUATION OF FRONTAL HEMORHHAGE | 2 | 2 | 2 | CEFTRIAXONE | 1 | 1,2 | 1 | 7 | 7 | 1 | 2 | 14 | 13 | 7 |
| 191/2221 | 8 | 1 | SEVERE ANEMIA, HEPATOMEGALY, CCF, RT U.L. CELLULITIS | 2 | 2 | 2 | VANCOMYCIN | 1 | 1,2 | 1 | 3 | 3 | 1 | 3 | 9 | 9 | 3 |
|  |  |  |  |  |  |  | MEROPENEM | 1 | 2 | 1 | 3 |  | 1 | 2 | 6 | 6 | 3 |
| 192/2221 | 1 | 1 | MIXED GERM CELL TUMOR OF LT TESTIS, POST ORCHIDECTOMY | 2 | 2 | 3 | CEFTRIAXONE | 1 | 1,2 | 1 | 3 | 3 | 1 | 2 | 6 | 6 | 3 |
|  |  |  |  |  |  |  | METRONIDAZOLE | 1 | 1,2 | 1 | 3 |  | 1 | 3 | 9 | 9 | 3 |
| 193/2221 | 12 | 1 | HSM, CERVICAL LYMPHADENOPATHY, AML | 2 | 2 | 3 | VANCOMYCIN | 1 | 1,2 | 1 | 3 | 3 | 1 | 2 | 6 | 4 | 3 |
|  |  |  |  |  |  |  | MEROPENEM | 1 | 2 | 1 | 3 |  | 1 | 2 | 6 | 4 | 3 |
| 194/2221 | 2.42 | 2 | ACUTE ENCEPHALOPATHY, HEPATOMEGALY, IEM | 2 | 2 | 12 | MEROPENEM | 1 | 2 | 1 | 3 | 12 | 1 | 1 | 3 | 3 | 3 |
|  |  |  |  |  |  |  | PIPERACILLIN+TAZOBACTAM | 2 | 1,2 | 1 | 9 |  | 1 | 3 | 36 | 33 | 9 |
|  |  |  |  |  |  |  | RIFAXIMIN | 1 | 2 | 3 | 3 |  | 1 | 3 | 9 | 8 | 3 |
| 195/2221 | 12 | 2 | SLE, ACUTE IMMUNOENCEPHALITIS | 2 | 2 | 4 | COLISTIN | 1 | 2 | 1 | 4 | 4 | 1 | 3 | 12 | 12 | 4 |
|  |  |  |  |  |  |  | LINEZOLID | 1 | 1,2 | 1 | 4 |  | 1 | 2 | 8 | 7 | 4 |
| 196/2221 | 2.75 | 1 | STATUS EPILEPTICUS, ENCEPHALOPATHY | 2 | 2 | 5 | MEROPENEM | 1 | 1,2 | 1 | 5 | 5 | 1 | 3 | 15 | 13 | 5 |
|  |  |  |  |  |  |  | AMIKACIN | 1 | 2 | 1 | 4 |  | 1 | 1 | 4 | 4 | 4 |
| 197/2221 | 1 | 2 | CHRONIC LIVER DISEASE, HEMOSIDEROSIS, GAUCHERS DISEASE | 1,2,3 | 3 | 6 | CEFOTAXIME | 1 | 1,2 | 1 | 3 | 6 | 1 | 4 | 12 | 12 | 3 |
|  |  |  |  |  |  |  | AMPICILLIN+CLOXACILLIN | 2 | 2 | 1 | 3 |  | 1 | 4 | 12 | 12 | 3 |
| 198/2221 | 13 | 2 | CKD, CCF, LT SIDED PNEUMONIA, SNHL | 2 | 2 | 4 | CEFTRIAXONE | 1 | 1,2 | 1 | 2 | 4 | 1 | 2 | 4 | 3 | 2 |
|  |  |  |  |  |  |  | AZITHROMYCIN | 1 | 1,2 | 2 | 2 |  | 1 | 1 | 2 | 2 | 2 |
| 199/2221 | 1 | 2 | CHRONIC LIVER DISEASE | 2 | 2 | 3 | AMOXYCILLIN+CLAVULANATE | 2 | 1,2 | 2 | 5 | 3 | 1 | 3 | 15 | 14 | 5 |
| 200/2221 | 13 | 2 | STATUS EPILEPTICUS | 1,2,3 | 6 | 6 | CEFTRIAXONE | 1 | 1,2 | 1 | 5 | 6 | 1 | 2 | 12 | 12 | 5 |
|  |  |  |  |  |  |  | AZITHROMYCIN | 1 | 1,2 | 2 | 2 |  | 1 | 1 | 2 | 2 | 2 |
| 201/2221 | 2.92 | 1 | EPILEPTIC ENCEPHALOPATHY | 2 | 2 | 4 | CEFTRIAXONE | 1 | 1,2 | 1 | 3 | 3 | 1 | 2 | 6 | 4 | 3 |
| 202/2221 | 8 | 1 | CHF, RT PLEURAL EFFUSION, ATN | 2 | 2 | 11 | PIPERACILLIN+TAZOBACTAM | 2 | 1,2 | 1 | 11 | 11 | 1 | 3 | 33 | 33 | 11 |
| 203/2221 | 13 | 2 | GLOMERULONEPHRITIS, HTN | 2 | 2 | 6 | CEFTRIAXONE | 1 | 1,2 | 1 | 2 | 6 | 1 | 2 | 4 | 3 | 2 |
|  |  |  |  |  |  |  | PIPERACILLIN+TAZOBACTAM | 2 | 1,2 | 1 | 6 |  | 1 | 3 | 18 | 17 | 6 |
| 204/2221 | 2.58 | 1 | CHD, CCF, SEVERE PAH, PNEUMONIA | 1,2,3 | 9 | 14 | MEROPENEM | 1 | 2 | 1 | 14 | 14 | 1 | 3 | 42 | 41 | 14 |
|  |  |  |  |  |  |  | VANCOMYCIN | 1 | 1,2 | 1 | 4 |  | 1 | 4 | 16 | 15 | 4 |
|  |  |  |  |  |  |  | COLISTIN | 1 | 2 | 1 | 9 |  | 1 | 3 | 27 | 26 | 9 |
| 205/2221 | 8 | 2 | SLE | 2 | 2 | 4 | CEFTRIAXONE | 1 | 1,2 | 1 | 4 | 4 | 1 | 2 | 8 | 7 | 4 |
|  |  |  |  |  |  |  | CLINDAMYCIN | 1 | 1,2 | 1 | 4 |  | 1 | 3 | 12 | 12 | 4 |
| 206/2221 | 2.75 | 1 | PUV POST OP CYSTOSCOPY, PUV FULGRATION | 2 | 2 | 4 | CEFTRIAXONE | 1 | 1,2 | 1 | 3 | 3 | 1 | 2 | 6 | 5 | 3 |
| 207/2221 | 2.25 | 2 | CYANOTIC HEART DISEASE, PDA | 2 | 2 | 8 | AMIKACIN | 1 | 2 | 1 | 7 | 7 | 1 | 1 | 7 | 7 | 7 |
|  |  |  |  |  |  |  | PIPERACILLIN+TAZOBACTAM | 2 | 1,2 | 1 | 7 |  | 1 | 3 | 21 | 21 | 7 |
| 208/2221 | 8 | 1 | ARDS, ACUTE ENCEPHALITIS SYNDROME, CCF | 2 | 2 | 5 | AMIKACIN | 1 | 2 | 1 | 5 | 5 | 1 | 1 | 5 | 5 | 5 |
|  |  |  |  |  |  |  | PIPERACILLIN+TAZOBACTAM | 2 | 1,2 | 1 | 5 |  | 1 | 3 | 15 | 15 | 5 |
| 209/2221 | 1 | 1 | MENINGITIS | 2 | 2 | 8 | CEFTRIAXONE | 1 | 1,2 | 1 | 8 | 8 | 1 | 2 | 16 | 15 | 8 |
| 210/2221 | 11 | 1 | AKI | 2 | 2 | 9 | MEROPENEM | 1 | 2 | 1 | 9 | 9 | 1 | 2 | 18 | 16 | 9 |
|  |  |  |  |  |  |  | AZITHROMYCIN | 1 | 1,2 | 1 | 1 |  | 1 | 1 | 1 | 1 | 1 |
|  |  |  |  |  |  |  | PIPERACILLIN+TAZOBACTAM | 2 | 1,2 | 1 | 1 |  | 1 | 3 | 3 | 1 | 1 |
| 211/2221 | 3 | 2 | NEUROBLASTOMA | 2 | 2 | 4 | CEFTRIAXONE | 1 | 1,2 | 1 | 4 | 4 | 1 | 2 | 8 | 6 | 4 |
| 212/2221 | 2 | 1 | ALL | 1,2,3 | 4 | 7 | CEFTAZIDIME | 1 | 1,2 | 1 | 1 | 6 | 1 | 3 | 3 | 2 | 1 |
|  |  |  |  |  |  |  | AMIKACIN | 1 | 2 | 1 | 1 |  | 1 | 1 | 1 | 1 | 1 |
|  |  |  |  |  |  |  | MEROPENEM | 1 | 2 | 1 | 6 |  | 1 | 3 | 18 | 16 | 6 |
|  |  |  |  |  |  |  | VANCOMYCIN | 1 | 1,2 | 1 | 6 |  | 1 | 4 | 24 | 22 | 6 |
| 213/2221 | 14 | 2 | SUPERIOR MEDIASTINAL SYNDROME | 2 | 2 | 4 | COTRIMOXAZOLE | 2 | 1,2 | 2 | 3 | 3 | 1 | 1 | 3 | 3 | 3 |
| 214/2221 | 12 | 1 | MENINGITIS | 2 | 2 | 8 | MEROPENEM | 1 | 2 | 1 | 8 | 8 | 1 | 3 | 24 | 23 | 8 |
|  |  |  |  |  |  |  | VANCOMYCIN | 1 | 1,2 | 1 | 8 |  | 1 | 4 | 32 | 32 | 8 |
|  |  |  |  |  |  |  | METRONIDAZOLE | 1 | 1,2 | 1 | 2 |  | 1 | 3 | 6 | 3 | 2 |
| 215/2221 | 3 | 1 | TBM, T.T. IN SITU | 2 | 2 | 5 | MEROPENEM | 1 | 2 | 1 | 5 | 5 | 1 | 3 | 15 | 14 | 5 |
|  |  |  |  |  |  |  | LINEZOLID | 1 | 1,2 | 1 | 5 |  | 1 | 2 | 12 | 9 | 5 |
| 216/2221 | 11 | 2 | SLE | 2 | 2 | 4 | CEFTRIAXONE | 1 | 1,2 | 1 | 4 | 4 | 1 | 2 | 8 | 6 | 4 |

**Datasheet- II**

| **Column** | **Parameter** | **Code** |
| --- | --- | --- |
| A | Patient code | - |
| B | Sample sent for Culture sensitivity test | Yes=1, No=0 |
| C | Culture sensitivity report | Positive=1, Negative=0, Culture not done (Sample not sent for culture sensitivity) = N.A. |
| D | Type of Sample sent for culture | Blood=1, Urine=2, Pus=3, CSF=4, ET Aspirate=5, Sputum=6, Bronchoalveolar Lavage =7, Aspirated Fluid=8, Pleural Fluid=9, Throat Swab=10, Stool=11, Ascitic Fluid=12, Vaginal Swab=13, Synovial Fluid=14, Peritoneal Fluid=15, Not applicable= N.A. |
| E | Organism isolated | *E. Coli=1, Acinetobacter Baumanii=2, Pseudomonas=3, Klebsiella Pneumoniae=4, Acinetobacter Iwolfii=5, MRSA=6, MR CONS=7, Staphylococcus Hominis=8, MSSA=9, Staphylococcus Epidermidis=10, Staphylococcal Mitis/Oralis=11, Enterococcus Faecalis=12, Candida Tropicalis=14, Candida Krusei=15,* Not applicable= N.A. |
| F | Sensitive Drugs | Amikacin=1, Gentamicin=2, Doxycycline=3, Vancomycin=4, Colistin=5, Cotrimoxazole=6, Erythromycin=7, Clindamycin-8, Oxacillin=9, Tobramycin=10, Piperacillin Tazobactam=11, Penicillin=12, Ampicillin=13, Cefazolin=14, Cefepime=15, Meropenem=16, Imipenem=17, Levofloxacin=18, Ciprofloxacin=19, Ceftazidime=20, Cefotaxime=21, Aztreonam=22, Ampicillin-Sulbactam=23, Ceftriaxone=24, Cefuroxime=25, Amoxicillin Clavulanate=26, Nitrofurantoin=27, Norfloxacin=28, Fosfomycin=29, Amphotericin B=30, Fluconazole=31, Voriconazole=32, Caspofungin=33, Not applicable= N.A. |
| G | Intermediate DRUGS |  |
| H | Resistant DRUGS |  |
| I | Antimicrobial therapy | Escalation=1, De-escalation=2, Continuation=3 |
| J | Type of therapy | Empirical therapy=1, Targeted therapy=2 |
| K | Afebrile within 48 hours | Yes=1, No=0 |
| L | Readmission within 7 days | Yes=1, No=0 |
| M | Mortality | Yes=1, No=0 |

| **A** | **B** | **C** | **D** | **E** | **F** | **G** | **H** | **I** | **J** | **K** | **L** | **M** |
| --- | --- | --- | --- | --- | --- | --- | --- | --- | --- | --- | --- | --- |
| 001/2019 | 1 | 0 | 1 | 0 | 0 | 0 | 0 | 1 | 1 | 0 | 0 | 1 |
|  |  |  | 2 | 0 | 0 | 0 | 0 |  |  |  |  |  |
| 002/2019 | 1 | 0 | 2 | 0 | 0 | 0 | 0 | 2 | 1 | 0 | 0 | 0 |
|  |  |  | 2 | 0 | 0 | 0 | 0 |  |  |  |  |  |
|  |  |  | 1 | 0 | 0 | 0 | 0 |  |  |  |  |  |
|  |  |  | 1 | 1 | 1, 2 | 26 | 14,15,10,6 |  |  |  |  |  |
| 003/2019 | 0 | N.A. | N.A. | N.A. | N.A. | N.A. | N.A. | 1 | 1 | 0 | 0 | 1 |
| 004/2019 | 1 | 0 | 1 | 0 | 0 | 0 | 0 | 1 | 1 | 0 | 0 | 0 |
| 005/2019 | 1 | 0 | 2 | 0 | 0 | 0 | 0 | 1 | 1 | 0 | 0 | 1 |
| 006/2019 | 1 | 0 | 0 | 0 | 0 | 0 | 0 | 3 | 1 | 0 | 0 | 0 |
| 007/2019 | 1 | 0 | 13 | 0 | 0 | 0 | 0 | 3 | 1 | 0 | 0 | 1 |
| 008/2019 | 1 | 0 | 1 | 0 | 0 | 0 | 0 | 3 | 1 |  |  |  |
| 009/2019 | 1 | 1 | 1 | 7 | 3 | 0 | 12, 7, 6, 9 | 3 | 1 | 1 | 0 | 0 |
|  |  |  | 3 | 0 | 0 | 0 | 0 |  |  |  |  |  |
|  |  |  | 4 | 0 | 0 | 0 | 0 |  |  |  |  |  |
| 010/2019 | 1 | 0 | 1 | 0 | 0 | 0 | 0 | 3 | 1 | 1 | 0 | 0 |
| 011/2019 | 1 | 0 | 1 | 0 | 0 | 0 | 0 | 3 | 1 | 1 | 1 | 0 |
| 012/2019 | 0 | N.A. | N.A. | N.A. | N.A. | N.A. | N.A. | 1 | 1 | 1 | 0 | 0 |
| 013/2019 | 1 | 0 | 1 | 0 | 0 | 0 | 0 | 1 | 1 | 0 | 0 | 1 |
| 014/2019 | 1 | 0 | 1 | 0 | 0 | 0 | 0 | 3 | 1 | 1 | 0 | 0 |
| 015/2019 | 1 | 0 | 1 | 0 | 0 | 0 | 0 | 3 | 1 | 1 | 0 | 0 |
| 016/2019 | 1 | 0 | 1 | 0 | 0 | 0 | 0 | 3 | 1 | 1 | 0 | 0 |
| 017/2019 | 0 | N.A. | N.A. | N.A. | N.A. | N.A. | N.A. | 3 | 1 | 0 | 1 | 0 |
| 018/2019 | 1 | 0 | 5 | 0 | 0 | 0 | 0 | 3 | 1 | 1 | 0 | 0 |
|  |  |  | 2 | 0 | 0 | 0 | 0 |  |  |  |  |  |
|  |  |  | 1 | 0 | 0 | 0 | 0 |  |  |  |  |  |
| 019/2019 | 1 | 0 | 1 | 0 | 0 | 0 | 0 | 3 | 1 | 0 | 0 | 0 |
| 020/2019 | 1 | 0 | 1 | 0 | 0 | 0 | 0 | 1 |  | 0 | 0 | 0 |
| 021/2019 | 1 | 0 | 1 | 0 | 0 | 0 | 0 | 3 | 1 | 0 | 0 | 0 |
| 022/2019 | 0 | N.A. | N.A. | N.A. | N.A. | N.A. | N.A. | 3 | 1 | 0 | 0 | 0 |
| 023/2019 | 1 | 0 | 1 | 0 | 0 | 0 | 0 | 3 | 1 | 0 | 0 | 0 |
| 024/2019 | 1 | 0 | 1 | 0 | 0 | 0 | 0 | 3 | 1 | 0 | 0 | 0 |
| 025/2019 | 1 | 0 | 1 | 0 | 0 | 0 | 0 | 3 | 1 | 0 | 0 | 0 |
| 026/2019 | 1 | 1 | 6 | 6 | 4 | 3 | 6,7,8,9 | 1 | 2 | 0 | 0 | 0 |
|  |  |  | 6 | 6 | 5 | 22 | 1, 15, 20,19,16, 11 |  |  |  |  |  |
| 027/2019 | 1 | 0 | 7 | 0 | 0 | 0 | 0 | 1 | 1 | 0 | 1 | 0 |
| 028/2019 | 1 | 0 | 1 | 0 | 0 | 0 | 0 | 2 | 1 | 1 | 0 | 0 |
| 029/2019 | 1 | 0 | 1 | 0 | 0 | 0 | 0 | 2 | 1 | 0 | 0 | 0 |
| 030/2019 | 1 | 0 | 1 | 0 | 0 | 0 | 0 | 3 | 1 | 0 | 0 | 0 |
| 031/2019 | 1 | 0 | 1 | 0 | 0 | 0 | 0 | 2 | 1 | 0 | 0 | 0 |
| 032/2019 | 1 | 0 | 1 | 0 | 0 | 0 | 0 | 3 | 1 | 0 | 0 | 0 |
| 033/2019 | 1 | 0 | 1 | 0 | 0 | 0 | 0 | 3 | 1 | 0 | 0 | 0 |
| 034/2019 | 1 | 0 | 1 | 0 | 0 | 0 | 0 | 2 | 1 | 0 | 0 | 0 |
| 035/2019 | 0 | N.A. | N.A. | N.A. | N.A. | N.A. | N.A. | 3 | 1 | 0 | 0 | 0 |
| 036/2019 | 1 | 0 | 1 | 0 | 0 | 0 | 0 | 1 | 1 | 0 | 0 | 0 |
| 037/2019 | 0 | N.A. | N.A. | N.A. | N.A. | N.A. | N.A. | 3 | 1 | 0 | 0 | 0 |
| 038/2019 | 1 | 0 | 1 | 0 | 0 | 0 | 0 | 3 | 1 | 0 | 0 | 0 |
| 039/2019 | 1 | 0 | 1 | 0 | 0 | 0 | 0 | 1 | 1 | 0 | 0 | 0 |
| 040/2019 | 1 | 0 | 1 | 0 | 0 | 0 | 0 | 3 | 1 | 1 | 0 | 0 |
| 041/2019 | 1 | 0 | 1 | 0 | 0 | 0 | 0 | 3 | 1 | 0 | 0 | 0 |
| 042/2019 | 1 | 0 | 1 | 0 | 0 | 0 | 0 | 1 | 1 | 0 | 0 | 0 |
| 043/2020 | 1 | 1 | 8 | 2 | 5 | 0 | 19, 2, 16, 15, 11, 20 | 1 | 2 | 1 | 0 | 0 |
| 044/2020 | 1 | 0 | 1 | 0 | 0 | 0 | 0 | 3 | 1 | 0 | 0 | 0 |
| 045/2020 | 1 | 0 | 1 | 0 | 0 | 0 | 0 | 1 | 1 | 0 | 0 | 0 |
|  |  |  | 9 | 0 | 0 | 0 | 0 |  |  |  |  |  |
|  |  |  | 5 | 0 | 0 | 0 | 0 |  |  |  |  |  |
| 046/2020 | 1 | 0 | 1 | 0 | 0 | 0 | 0 | 2 | 1 | 0 | 0 | 0 |
| 047/2020 | 1 | 0 | 1 | 0 | 0 | 0 | 0 | 2 | 1 | 0 | 0 | 0 |
| 048/2020 | 1 | 0 | 1 | 0 | 0 | 0 | 0 | 3 | 1 | 0 | 0 | 0 |
| 049/2020 | 1 | 0 | 2 | 0 | 0 | 0 | 0 | 1 | 1 | 0 | 0 | 0 |
| 050/2020 | 1 | 0 | 8 | 0 | 0 | 0 | 0 | 1 | 1 | 1 | 0 | 0 |
|  |  |  | 1 | 0 | 0 | 0 | 0 |  | 1 |  |  |  |
| 051/2020 | 1 | 0 | 1 | 0 | 0 | 0 | 0 | 1 | 1 | 0 | 0 | 1 |
| 052/2020 | 0 | N.A. | N.A. | N.A. | N.A. | N.A. | N.A. | 1 | 1 | 1 | 0 | 0 |
| 053/2020 | 1 | 0 | 2 | 0 | 0 | 0 | 0 | 1 | 1 | 0 | 0 | 1 |
|  |  |  | 10 | 0 | 0 | 0 | 0 |  |  |  |  |  |
|  |  |  | 1 | 0 | 0 | 0 | 0 |  |  |  |  |  |
|  |  |  | 2 | 0 | 0 | 0 | 0 |  |  |  |  |  |
|  |  |  | 5 | 0 | 0 | 0 | 0 |  |  |  |  |  |
| 054/2020 | 1 | 0 | 1 | 0 | 0 | 0 | 0 | 2 |  | 1 | 0 | 0 |
| 055/2020 | 0 | N.A. | N.A. | N.A. | N.A. | N.A. | N.A. | 3 | 1 | 1 | 0 | 0 |
| 056/2020 | 0 | N.A. | N.A. | N.A. | N.A. | N.A. | N.A. | 3 | 1 | 1 | 0 | 0 |
| 057/2020 | 1 | 1 | 2 | 3 | 5 | 0 | 1,22,15,20,19,2,17,18,16,11,10 | 1 | 2 | 0 | 0 | 0 |
| 058/2020 | 1 | 0 | 1 | 0 | 0 | 0 | 0 | 1 | 1 | 0 | 0 | 0 |
| 059/2020 | 0 | N.A. | N.A. | N.A. | N.A. | N.A. | N.A. | 3 | 1 | 0 | 0 | 0 |
| 060/2020 | 1 | 0 | 1 | 0 | 0 | 0 | 0 | 1 | 1 | 0 | 0 | 0 |
| 061/2020 | 1 | 0 | 4 | 0 | 0 | 0 | 0 | 3 | 1 | 0 | 0 | 0 |
|  |  |  | 1 | 0 | 0 | 0 | 0 |  |  |  |  |  |
|  |  |  | 2 | 0 | 0 | 0 | 0 |  |  |  |  |  |
| 062/2020 | 0 | N.A. | N.A. | N.A. | N.A. | N.A. | N.A. | 3 | 1 | 0 | 0 | 0 |
| 063/2020 | 0 | N.A. | N.A. | N.A. | N.A. | N.A. | N.A. | 1 | 1 | 0 | 0 | 0 |
| 064/2020 | 0 | 1 | 2 | 12 | 13, 12 | 0 | 2 | 3 | 2 | 0 | 0 | 0 |
|  |  |  | 11 | 0 | 0 | 0 | 0 |  |  |  |  |  |
|  |  |  | 2 | 0 | 0 | 0 | 0 |  |  |  |  |  |
| 065/2020 | 0 | N.A. | N.A. | N.A. | N.A. | N.A. | N.A. | 3 | 1 | 0 | 0 | 0 |
| 066/2020 | 1 | 0 | 2 | 0 | 0 | 0 | 0 | 1 | 1 | 0 | 0 | 0 |
|  |  |  | 9 | 0 | 0 | 0 | 0 |  |  |  |  |  |
| 067/2020 | 1 | 0 | 2 | 0 | 0 | 0 | 0 | 3 | 1 | 0 | 0 | 0 |
| 068/2020 | 0 | N.A. | N.A. | N.A. | N.A. | N.A. | N.A. | 3 | 1 | 1 | 0 | 0 |
| 069/2020 | 1 | 1 | 5 | 2 | 0 | 0 | 1,23,15,19,2,17,16,11,18,20,21,6,24,10 | 1 | 1 | 1 | 0 | 0 |
|  |  |  | 1 | 0 | 0 | 0 | 0 |  |  |  |  |  |
|  |  |  | 1 | 0 | 0 | 0 | 0 |  |  |  |  |  |
| 070/2020 | 0 | N.A. | N.A. | N.A. | N.A. | N.A. | N.A. | 3 | 1 | 0 | 0 | 1 |
| 071/2020 | 1 | 0 | 10 | 0 | 0 | 0 | 0 | 3 | 1 | 0 | 0 | 0 |
|  |  |  | 8 | 4 | 0 | 0 | 0 |  |  |  |  |  |
| 072/2020 | 1 | 1 | 1 | 2 | 6 | 0 | 1,21,19,2,17,16,18,10,25,11,24 | 2 | 1 | 1 | 0 | 0 |
|  |  |  | 4 | 0 | 0 | 0 | 0 |  |  |  |  |  |
| 073/2020 | 1 | 0 | 1 | 0 | 0 | 0 | 0 | 3 | 1 | 1 | 0 | 0 |
|  |  |  | 9 | 0 | 0 | 0 | 0 |  |  |  |  |  |
| 074/2020 | 1 | 0 | 0 | 0 | 0 | 0 | 0 | 3 | 1 | 1 | 0 | 0 |
| 075/2020 | 1 | 1 | 3 | 1 | 1,2,11,10,6 | 0 | 0 | 1 | 1 | 1 | 0 | 0 |
| 076/2020 | 1 | 1 | 2 | 0 | 0 | 0 | 0 | 1 | 2 | 1 | 0 | 0 |
|  |  |  | 1 | 0 | 0 | 0 | 0 |  |  |  |  |  |
|  |  |  | 1 | 0 | 0 | 0 | 0 |  |  |  |  |  |
| 077/2020 | 1 | 1 | 2 | 0 | 0 | 0 | 0 | 2 | 2 | 1 | 0 | 0 |
|  |  |  | 11 | 1 | 15, 24,11,10,26 | 0 | 0 |  |  |  |  |  |
|  |  |  | 0 | 0 | 0 | 0 | 0 |  |  |  |  |  |
| 078/2020 | 0 | N.A. | N.A. | N.A. | N.A. | N.A. | N.A. | 1 | 1 | 1 | 0 | 0 |
| 079/2020 | 1 | 0 | 1 | 0 | 0 | 0 | 0 | 2 | 1 | 1 | 0 | 0 |
| 080/2020 | 1 | 0 | 9 | 0 | 0 | 0 | 0 | 3 | 1 | 1 | 0 | 0 |
|  |  |  | 8 | 0 | 0 | 0 | 0 |  |  |  |  |  |
|  |  |  | 1 | 6 | 8,6,7,9 | 0 | 12 |  |  |  |  |  |
| 081/2020 | 1 | 1 | 8 | 0 | 0 | 0 | 0 | 1 | 2 | 0 | 0 | 0 |
|  |  |  | 1 | 0 | 0 | 0 | 0 |  |  |  |  |  |
|  |  |  | 3 | 0 | 0 | 0 | 0 |  |  |  |  |  |
|  |  |  | 2 | 0 | 0 | 0 | 0 |  |  |  |  |  |
| 082/2020 | 1 | 0 | 1 | 0 | 0 | 0 | 0 | 3 | 1 | 1 | 0 | 0 |
|  |  |  | 1 | 8 | 4 | 0 | 0 |  |  |  |  |  |
| 083/2020 | 1 | 1 | 1 | 0 | 0 | 0 | 0 | 2 | 2 | 0 | 0 | 0 |
| 084/2020 | 1 | 1 | 1 | 0 | 0 | 0 | 0 | 3 | 1 | 0 | 0 | 0 |
|  |  |  | 1 | 1 | 0 | 0 | 1,13,14,15,21,24,25,2,17,18,16,11,10,6,26,19 |  |  |  |  |  |
|  |  |  | 1 | 3 | 15,20,2,11,10 | 0 | 0 |  |  |  |  |  |
|  |  |  | 1 | 8 | 4,3 | 0 | 12,7,8,6,9 |  |  |  |  |  |
| 085/2020 | 1 | 1 | 5 | 4 | 5 | 0 | 1,15,21,19F,2,17,16,18,10,6,11,24 | 2 | 1 | 0 | 0 | 0 |
|  |  |  | 5 | 3 | 5,22 | 0 | 1,15,21,19,2,17,16,18,10,11,24 |  |  |  |  |  |
|  |  |  | 1 | 0 | 0 | 0 | 0 |  |  |  |  |  |
| 086/2020 | 1 | 0 | 2 | 0 | 0 | 0 | 0 | 3 | 1 | 0 | 0 | 0 |
|  |  |  | 1 | 0 | 0 | 0 | 0 |  |  |  |  |  |
| 087/2020 | 1 | 0 | 2 | 0 | 0 | 0 | 0 | 2 | 1 | 0 | 0 | 0 |
|  |  |  | 1 | 0 | 0 | 0 | 0 |  |  |  |  |  |
| 088/2020 | 1 | 0 | 1 | 0 | 0 | 0 | 0 | 2 | 1 | 0 | 0 | 0 |
| 089/2020 | 1 | 0 | 0 | 0 | 0 | 0 | 0 | 3 | 1 | 0 | 0 | 0 |
| 090/2020 | 0 | N.A. | N.A. | N.A. | N.A. | N.A. | N.A. | 3 | 1 | 0 | 0 | 1 |
| 091/2020 | 0 | N.A. | N.A. | N.A. | N.A. | N.A. | N.A. | 2 | 1 | 1 | 0 | 0 |
| 092/2020 | 1 | 0 | 4 | 0 | 0 | 0 | 0 | 3 | 1 | 1 | 0 | 0 |
| 093/2020 | 1 | 1 | 1 | 4 | 2, 6 | 1,10 | 1,13,23, 14, 15, 21, 20, 24, 17, 16, 18, 11, 10, 6 | 1 | 1 | 0 | 0 | 1 |
|  |  |  | 1 | 0 | 0 | 0 | 0 |  |  |  |  |  |
| 094/2020 | 1 | 0 | 2 | 0 | 0 | 0 | 0 | 1 | 1 | 0 | 0 | 0 |
|  |  |  | 1 | 0 | 0 | 0 | 0 |  |  |  |  |  |
| 095/2020 | 1 | 0 | 3 | 0 | 0 | 0 | 0 | 3 | 1 | 1 | 0 | 0 |
|  |  |  | 11 | 0 | 0 | 0 | 0 |  |  |  |  |  |
| 096/2020 | 1 | 1 | 1 | 1 | 5 | 0 | 1,13,23, 14, 15, 21, 20, 24, 17, 16, 18, 11, 10, 6 | 1 | 1 | 0 | 0 | 0 |
| 097/2020 | 0 | N.A. | N.A. | N.A. | N.A. | N.A. | N.A. | 2 | 1 | 0 | 0 | 0 |
| 098/2020 | 1 | 0 | 4 | 0 | 0 | 0 | 0 | 2 | 1 | 0 | 0 | 0 |
| 099/2020 | 1 | 0 | 1 | 0 | 0 | 0 | 0 | 3 | 1 | 0 | 0 | 0 |
|  |  |  | 2 | 0 | 0 | 0 | 0 |  |  |  |  |  |
| 100/2020 | 0 | N.A. | N.A. | N.A. | N.A. | N.A. | N.A. | 3 | 1 | 0 | 0 | 0 |
| 101/2020 | 1 | 0 | 12 | 0 | 0 | 0 | 0 | 3 | 1 | 0 | 0 | 0 |
|  |  |  | 2 | 0 | 0 | 0 | 0 |  |  |  |  |  |
|  |  |  | 1 | 0 | 0 | 0 | 0 |  |  |  |  |  |
| 102/2020 | 1 | 0 | 1 | 0 | 0 | 0 | 0 | 2 | 1 | 0 | 0 | 0 |
| 103/2020 | 1 | 0 | 1 | 0 | 0 | 0 | 0 | 1 | 1 | 0 | 0 | 1 |
|  |  |  | 2 | 0 | 0 | 0 | 0 |  |  |  |  |  |
| 104/2020 | 1 | 0 | 1 | 0 | 0 | 0 | 0 | 2 | 1 | 0 | 0 | 0 |
|  |  |  | 12 | 0 | 0 | 0 | 0 |  |  |  |  |  |
|  |  |  | 1 | 0 | 0 | 0 | 0 |  |  |  |  |  |
| 105/2020 | 1 | 0 | 1 | 0 | 0 | 0 | 0 | 1 | 1 | 1 | 0 | 0 |
| 106/2020 | 1 | 0 | 4 | 0 | 0 | 0 | 0 | 2 | 1 | 1 | 0 | 0 |
| 107/2020 | 1 | 0 | 0 | 0 | 0 | 0 | 0 | 2 | 1 | 1 | 0 | 0 |
| 108/2020 | 0 | N.A. | N.A. | N.A. | N.A. | N.A. | N.A. | 3 | 1 | 1 | 0 | 0 |
| 109/2020 | 1 | 0 | 1 | 0 | 0 | 0 | 0 | 2 | 1 | 1 | 0 | 1 |
| 110/2020 | 0 | N.A. | N.A. | N.A. | N.A. | N.A. | N.A. | 2 | 1 | 1 | 0 | 0 |
| 111/2020 | 1 | 0 | 1 | 0 | 0 | 0 | 0 | 2 | 1 | 1 | 0 | 0 |
| 112/2020 | 1 | 0 | 1 | 0 | 0 | 0 | 0 | 1 | 1 | 1 | 0 | 0 |
| 113/2020 | 1 | 0 | 8 | 0 | 0 | 0 | 0 | 2 | 1 | 1 | 0 | 0 |
| 114/2020 | 1 | 0 | 1 | 0 | 0 | 0 | 0 | 1 | 1 | 1 | 0 | 0 |
| 115/2020 | 1 | 0 | 0 | 0 | 0 | 0 | 0 | 1 | 1 | 1 | 0 | 1 |
| 116/2020 | 0 | N.A. | N.A. | N.A. | N.A. | N.A. | N.A. | 3 | 1 | 1 | 0 | 0 |
| 117/2020 | 1 | 1 | 2 | 0 | 0 | 0 | 0 | 1 | 2 | 1 | 0 | 0 |
|  |  |  | 8 | 2 | 5 | 0 | 15, 19, 2, 17, 18, 16, 11, 20, 6, 24, 10 |  |  |  |  |  |
|  |  |  | 1 | 0 | 0 | 0 | 0 |  |  |  |  |  |
| 118/2020 | 1 | 0 | 0 | 0 | 0 | 0 | 0 | 3 | 1 | 1 | 0 | 0 |
| 119/2020 | 0 | N.A. | N.A. | N.A. | N.A. | N.A. | N.A. | 2 | 1 | 1 | 0 | 0 |
| 120/2020 | 0 | N.A. | N.A. | N.A. | N.A. | N.A. | N.A. | 3 | 1 | 1 | 0 | 0 |
| 121/2020 | 1 | 0 | 1 | 0 | 0 | 0 | 0 | 3 | 1 | 1 | 0 | 0 |
| 122/2020 | 1 | 0 | 1 | 0 | 0 | 0 | 0 | 2 | 1 | 1 | 0 | 0 |
| 123/2020 | 1 | 0 | 1 | 2 | 5 | 0 | ALL | 1 | 1 | 1 | 0 | 0 |
| 124/2020 | 1 | 1 | 1 | 0 | 0 | 0 | 0 | 1 | 2 | 1 | 0 | 0 |
| 125/2020 | 1 | 0 | 8 | 9 | 8,6,7,9 | 0 | 12 | 3 | 1 | 1 | 0 | 0 |
| 126/2020 | 1 | 1 | 1 | 0 | 0 | 0 | 0 | 1 | 2 | 1 | 0 | 0 |
|  |  |  | 3 | 0 | 0 | 0 | 0 |  |  |  |  |  |
|  |  |  | 2 | 0 | 0 | 0 | 0 |  |  |  |  |  |
|  |  |  | 1 | 8 | 7,8,9 | 0 | 12, 6 |  |  |  |  |  |
| 127/2020 | 1 | 1 | 1 | 0 | 0 | 0 | 0 | 2 | 2 | 1 | 0 | 0 |
| 128/2020 | 1 | 0 | 2 | 0 | 0 | 0 | 0 | 3 | 1 | 1 | 0 | 0 |
|  |  |  | 14 | 0 | 0 | 0 | 0 |  |  |  |  |  |
|  |  |  | 11 | 0 | 0 | 0 | 0 |  |  |  |  |  |
|  |  |  | 1 | 0 | 0 | 0 | 0 |  |  |  |  |  |
| 129/2020 | 1 | 0 | 1 | 0 | 0 | 0 | 0 | 2 | 1 | 1 | 0 | 0 |
| 130/2020 | 1 | 0 | 1 | 0 | 0 | 0 | 0 | 2 | 1 | 1 | 0 | 0 |
| 131/2020 | 1 | 1 | 2 | 0 | 0 | 0 | 0 | 3 | 2 | 1 | 0 | 0 |
|  |  |  | 3 | 6 | 8 | 0 | 0 |  |  |  |  |  |
|  |  |  | 3 | 1 | 8 | 0 | 0 |  |  |  |  |  |
|  |  |  | 1 | 0 | 0 | 0 | 0 |  |  |  |  |  |
| 132/2020 | 1 | 0 | 1 | 9 | 0 | 0 | 0 | 3 | 1 | 1 | 0 | 0 |
| 133/2020 | 1 | 1 | 4 | 0 | 0 | 0 | 0 | 2 | 2 | 1 | 0 | 0 |
|  |  |  | 8 | 3 | 2, 10, 11 | 20 | 0 |  |  |  |  |  |
|  |  |  | 1 | 0 | 0 | 0 | 0 |  |  |  |  |  |
| 134/2020 | 1 | 0 | 1 | 0 | 0 | 0 | 0 | 3 | 1 | 1 | 0 | 0 |
| 135/2020 | 1 | 0 | 1 | 0 | 0 | 0 | 0 | 3 | 1 | 1 | 0 | 0 |
| 136/2020 | 1 | 0 | 4 | 0 | 0 | 0 | 0 | 3 | 1 | 1 | 0 | 0 |
| 137/2020 | 1 | 1 | 2 | 0 | 0 | 0 | 0 | 1 | 2 | 0 | 0 | 0 |
|  |  |  | 1 | 7 | 6, 3, 4 | 0 | 12, 7, 8, 9 |  |  |  |  |  |
|  |  |  | 1 | 0 | 0 | 0 | 0 |  |  |  |  |  |
|  |  |  | 1 | 0 | 0 | 0 | 0 |  |  |  |  |  |
|  |  |  | 1 | 0 | 0 | 0 | 0 |  |  |  |  |  |
|  |  |  | 2 | 0 | 0 | 0 | 0 |  |  |  |  |  |
|  |  |  | 1 | 0 | 0 | 0 | 0 |  |  |  |  |  |
|  |  |  | 2 | 0 | 0 | 0 | 0 |  |  |  |  |  |
| 138/2020 | 1 | 0 | 2 | 0 | 0 | 0 | 0 | 2 | 1 | 0 | 0 | 0 |
|  |  |  | 1 | 0 | 0 | 0 | 0 |  |  |  |  |  |
| 139/2020 | 1 | 0 | 1 | 0 | 0 | 0 | 0 | 1 | 1 | 0 | 0 | 1 |
| 140/2020 | 1 | 0 | 4 | 0 | 0 | 0 | 0 | 1 | 1 | 0 | 0 | 0 |
|  |  |  | 1 | 0 | 0 | 0 | 0 |  |  |  |  |  |
| 141/2020 | 1 | 0 | 2 | 0 | 0 | 0 | 0 | 2 | 1 | 0 | 0 | 1 |
|  |  |  | 1 | 0 | 0 | 0 | 0 |  |  |  |  |  |
| 142/2020 | 1 | 0 | 1 | 0 | 0 | 0 | 0 | 3 | 1 | 0 | 0 | 0 |
|  |  |  | 2 | 1 | 27 | 0 | 0 |  |  |  |  |  |
| 143/2020 | 1 | 1 | 0 | 0 | 0 | 0 | 0 | 3 | 1 | 0 | 0 | 0 |
| 144/2020 | 1 | 0 | 15 | 0 | 0 | 0 | 0 | 3 | 1 | 0 | 0 | 0 |
| 145/2020 | 0 | N. A | N.A. | N.A. | N.A. | N.A. | N.A. | 3 | 1 | 1 | 0 | 0 |
| 146/2020 | 1 | 0 | 0 | 0 | 0 | 0 | 0 | 3 | 1 | 0 | 0 | 0 |
| 147/2020 | 1 | 0 | 1 | 0 | 0 | 0 | 0 | 3 | 1 | 0 | 0 | 1 |
| 148/2020 | 1 | 0 | 1 | 0 | 0 | 0 | 0 | 2 | 1 | 0 | 0 | 0 |
| 149/2020 | 1 | 0 | 4 | 0 | 0 | 0 | 0 | 1 | 1 | 0 | 0 | 0 |
| 150/2020 | 1 | 0 | 1 | 0 | 0 | 0 | 0 | 3 | 1 | 0 | 0 | 0 |
| 151/2020 | 1 | 0 | 0 | 0 | 0 | 0 | 0 | 1 | 1 | 0 | 0 | 0 |
|  |  |  | 12 | 0 | 0 | 0 | 0 |  |  |  |  |  |
| 152/2020 | 1 | 0 | 1 | 0 | 0 | 0 | 0 | 3 | 1 | 1 | 0 | 0 |
|  |  |  | 8 | 0 | 0 | 0 | 0 |  |  |  |  |  |
| 153/2020 | 1 | 0 | 12 | 0 | 0 | 0 | 0 | 2 | 1 | 0 | 0 | 1 |
| 154/2020 | 1 | 0 | 4 | 0 | 0 | 0 | 0 | 3 | 1 | 0 | 0 | 0 |
| 155/2020 | 1 | 0 | 1 | 0 | 0 | 0 | 0 | 1 | 1 | 0 | 0 | 1 |
|  |  |  | 1 | 0 | 0 | 0 | 0 |  |  |  |  |  |
| 156/2020 | 1 | 0 | 2 | 0 | 0 | 0 | 0 | 1 | 1 | 0 | 0 | 0 |
|  |  |  | 2 | 14 | 31, 33, 30 | 0 | 0 |  |  |  |  |  |
| 0157/2020 | 1 | 0 | 8 | 0 | 0 | 0 | 0 | 2 | 1 | 0 | 1 | 0 |
|  |  |  | 1 | 0 | 0 | 0 | 0 |  |  |  |  |  |
| 0158/2020 | 1 | 0 | 4 | 0 | 0 | 0 | 0 | 2 | 1 | 0 | 1 | 0 |
|  |  |  | 1 | 0 | 0 | 0 | 0 |  |  |  |  |  |
|  |  |  | 8 | 2 | 5 | 0 | 1, 23, 15,19,2,17,16,18,20,6,10 |  |  |  |  |  |
| 159/2020 | 1 | 1 | 0 | 0 | 0 | 0 | 0 | 2 | 1 | 0 | 0 | 0 |
| 160/2020 | 1 | 1 | 12 | 0 | 0 | 0 | 0 | 2 | 1 | 0 | 0 | 1 |
|  |  |  | 1 | 5 | 2, 10 | 0 | 23,20,19,11 |  |  |  |  |  |
| 161/2020 | 1 | 1 | 2 | 1 | 2, 27, 6 | 0 | 13, 14, 10 | 3 | 1 | 1 | 0 | 0 |
| 162/2020 | 1 | 1 | 8 | 0 | 0 | 0 | 0 | 2 | 2 | 0 | 0 | 0 |
|  |  |  | 2 | 14 | 31, 32, 30 | 0 | 0 |  |  |  |  |  |
|  |  |  | 12 | 0 | 0 | 0 | 0 |  |  |  |  |  |
|  |  |  | 9 | 0 | 0 | 0 | 0 |  |  |  |  |  |
| 163/2020 | 1 | 0 | 1 | 0 | 0 | 0 | 0 | 3 | 1 | 1 | 0 | 0 |
| 164/2020 | 1 | 0 | 1 | 0 | 0 | 0 | 0 | 1 | 1 | 1 | 0 | 0 |
|  |  |  | 1 | 0 | 0 | 0 | 0 |  |  |  |  |  |
|  |  |  | 2 | 0 | 0 | 0 | 0 |  |  |  |  |  |
| 165/2020 | 1 | 0 | 12 | 0 | 0 | 0 | 0 | 3 | 1 | 0 | 0 | 1 |
|  |  |  | 1 | 10 | 6 | 0 | 7 |  |  |  |  |  |
| 166/2020 | 1 | 1 | 1 | 11 | 8 | 0 | 12, 9 | 3 | 1 | 1 | 0 | 0 |
|  |  |  | 1 | 9 | 7, 9 | 0 | 6, 12 |  |  |  |  |  |
| 167/2020 | 1 | 1 | 0 | 0 | 0 | 0 | 0 | 1 | 1 | 1 | 0 | 0 |
| 168/2020 | 0 | N.A. | 0 | 0 | 0 | 0 | 0 | 3 | 1 | 1 | 1 | 0 |
| 169/2020 | 1 | 0 | 0 | 0 | 0 | 0 | 0 | 2 | 1 | 1 | 0 | 0 |
|  |  |  | 1 | 0 | 0 | 0 | 0 |  |  |  |  |  |
| 170/2020 | 1 | 0 | 1 | 0 | 0 | 0 | 0 | 2 | 1 | 1 | 0 | 0 |
| 171/2020 | 1 | 0 | 0 | 0 | 0 | 0 | 0 | 3 | 1 | 1 | 0 | 0 |
| 172/2020 | 1 | 0 | 2 | 0 | 0 | 0 | 0 |  | 1 | 1 | 0 | 0 |
|  |  |  | 1 | 15 | 30, 33 | 0 | 31, 32 |  |  |  |  |  |
| 173/2020 | 1 | 0 | 0 | 0 | 0 | 0 | 0 | 2 | 1 | 0 | 0 | 1 |
| 174/2020 | 1 | 0 | 1 | 0 | 0 | 0 | 0 | 2 | 1 | 1 | 0 | 1 |
|  |  |  | 8 | 0 | 0 | 0 | 0 |  |  |  |  |  |
| 175/2020 | 1 | 0 | 1 | 0 | 0 | 0 | 0 | 1 | 1 | 1 | 0 | 0 |
|  |  |  | 1 | 0 | 0 | 0 | 0 |  |  |  |  |  |
| 176/2020 | 1 | 0 | 11 | 0 | 0 | 0 | 0 | 3 | 1 | 1 | 0 | 0 |
| 177/2020 | 1 | 1 | 2 | 4 | NO | 0 | 1,23,15,21,19,2,17,16,18,27,10,6,11,5,24,25 | 2 | 2 | 1 | 0 | 0 |
|  |  |  | 1 | 12 | 13,12,27,29 | 0 | 28,19 |  |  |  |  |  |
|  |  |  | 4 | 0 | 0 | 0 | 0 |  |  |  |  |  |
| 178/2020 | 1 | 0 | 1 | 0 | 0 | 0 | 0 | 3 | 1 | 1 | 1 | 1 |
| 179/2021 | 1 | 0 | 1 | 0 | 0 | 0 | 0 | 3 | 1 | 1 | 0 | 1 |
| 180/2021 | 1 | 0 | 1 | 0 | 0 | 0 | 0 | 1 | 1 | 0 | 0 | 1 |
|  |  |  | 1 | 0 | 0 | 0 | 0 |  |  |  |  |  |
|  |  |  | 1 | 0 | 0 | 0 | 0 |  |  |  |  |  |
| 181/2021 | 1 | 0 | 1 | 0 | 0 | 0 | 0 | 3 | 1 | 1 | 0 | 0 |
| 182/2021 | 1 | 0 | 2 | 0 | 0 | 0 | 0 | 3 | 1 | 1 | 0 | 0 |
|  |  |  | 1 | 0 | 0 | 0 | 0 |  |  |  |  |  |
|  |  |  | 8 | 2 | 23, 6 | 0 | 1,15,19,2,11,20,21,24,10 |  |  |  |  |  |
| 183/2021 | 1 | 1 | 1 | 0 | 0 | 0 | 0 | 2 | 2 | 0 | 0 | 1 |
|  |  |  | 8 | 0 | 0 | 0 | 0 |  |  |  |  |  |
| 184/2021 | 1 | 0 | 0 | 0 | 0 | 0 | 0 | 1 | 1 | 0 | 0 | 0 |
| 185/2021 | 1 | 0 | 4 | 0 | 0 | 0 | 0 | 3 | 1 | 1 | 0 | 0 |
| 186/2021 | 1 | 0 | 0 | 0 | 0 | 0 | 0 | 3 | 1 | 1 | 0 | 0 |
| 187/2021 | 1 | 0 | 2 | 0 | 0 | 0 | 0 | 1 | 1 | 0 | 0 | 1 |
| 188/2021 | 1 | 0 | 1 | 2 | 23, 19, 20, 10 | 0 | N.A. | 2 | 1 | 0 | 0 | 0 |
| 189/2021 | 1 | 1 | 0 | 0 | 0 | 0 | 0 | 2 | 1 | 0 | 0 | 0 |
| 190/2021 | 1 | 0 | 0 | 0 | 0 | 0 | 0 | 2 | 1 | 1 | 0 | 0 |
| 191/2021 | 0 | N.A. | N.A. | N.A. | N.A. | N.A. | N.A. | 3 | 1 | 0 | 0 | 0 |
| 192/2021 | 0 | N.A. | N.A. | N.A. | N.A. | N.A. | N.A. | 3 | 1 | 0 | 0 | 0 |
| 193/2021 | 1 | 0 | 1 | 0 | 0 | 0 | 0 | 3 | 1 | 0 | 0 | 1 |
| 194/2021 | 1 | 0 | 0 | 0 | 0 | 0 | 0 | 1 | 1 | 0 | 0 | 1 |
| 195/2021 | 1 | 0 | 1 | 0 | 0 | 0 | 0 | 2 | 1 | 0 | 0 | 0 |
| 196/2021 | 1 | 0 | 0 | 0 | 0 | 0 | 0 | 2 | 1 | 1 | 0 | 0 |
| 197/2021 | 1 | 0 | 1 | 0 | 0 | 0 | 0 | 3 | 1 | 0 | 0 | 1 |
| 198/2021 | 1 | 0 | 0 | 0 | 0 | 0 | 0 | 3 | 1 | 1 | 0 | 0 |
| 199/2021 | 0 | N.A. | N.A. | N.A. | N.A. | N.A. | N.A. | 3 | 1 | 0 | 0 | 0 |
| 200/2021 | 0 | N.A. | N.A. | N.A. | N.A. | N.A. | N.A. | 2 | 1 | 0 | 0 | 1 |
| 201/2021 | 0 | N.A. | N.A. | N.A. | N.A. | N.A. | N.A. | 2 | 1 | 0 | 0 | 0 |
| 202/2021 | 0 | N.A. | N.A. | N.A. | N.A. | N.A. | N.A. | 3 | 1 | 0 | 0 | 0 |
| 203/2021 | 1 | 0 | 1 | 0 | 0 | 0 | 0 | 1 | 1 | 0 | 0 | 0 |
|  |  |  | 8 | 0 | 0 | 0 | 0 |  |  |  |  |  |
| 204/2021 | 1 | 0 | 2 | 0 | 0 | 0 | 0 | 2 | 1 | 0 | 0 | 0 |
| 205/2021 | 0 | N.A. | 0 | 0 | 0 | 0 | 0 | 3 | 1 | 0 | 0 | 0 |
| 206/2021 | 1 | 0 | 2 | 0 | 0 | 0 | 0 | 3 | 1 | 0 | 0 | 0 |
| 207/2021 | 1 | 0 | 2 | 0 | 0 | 0 | 0 | 3 | 1 | 0 | 0 | 0 |
| 208/2021 | 1 | 0 | 4 | 0 | 0 | 0 | 0 | 3 | 1 | 0 | 0 | 0 |
|  |  |  | 1 | 0 | 0 | 0 | 0 |  |  |  |  |  |
| 209/2021 | 1 | 0 | 0 | 0 | 0 | 0 | 0 | 3 | 1 | 0 | 0 | 0 |
| 210/2021 | 0 | N.A. | N.A. | N.A. | N.A. | N.A. | N.A. | 1 | 1 | 0 | 0 | 0 |
| 211/2021 | 0 | N.A. | N.A. | N.A. | N.A. | N.A. | N.A. | 3 | 1 | 0 | 0 | 0 |
| 212/2021 | 1 | 0 | 0 | 0 | 0 | 0 | 0 | 1 | 1 | 0 | 0 | 1 |
| 213/2020 | 0 | N.A. | 3 | 1 | 15,2,10, 6 | 0 | 14, 21, 25, 26 | 3 | 1 | 0 | 0 | 0 |
|  |  |  | 3 | 1 | 10, 6 | 0 | 13, 14, 25, 26 |  |  |  |  |  |
| 214/2021 | 1 | 1 | 0 | 0 | 0 | 0 | 0 | 1 | 1 | 0 | 0 | 0 |
| 215/2021 | 0 | N.A. | N.A. | N.A. | N.A. | N.A. | N.A. | 3 | 1 | 0 | 0 | 0 |
| 216/2021 | 0 | N.A. | N.A. | N.A. | N.A. | N.A. | N.A. | 3 | 1 | 0 | 0 | 0 |
