# Supplementary material for: “Antimicrobial utilization in a paediatric intensive care unit in India: A step towards strengthening antimicrobial stewardship practices"
Source: PLoS One. 2024 Sep 19;19(9):e0310515. doi: 10.1371/journal.pone.0310515 (PMC11412675; doi:10.1371/journal.pone.0310515)
Supplement: Attachment — Submitted filename: PONE-D-24-03393_KC comments.pdf [file pone.0310515.s009.pdf]

PLOS ONE

"Antimicrobial utilization in a paediatric intensive care unit in India: a step towards strengthening antimicrobial stewardship practices"

--Manuscript Draft--

**Manuscript Number:**	PONE-D-24-03393
**Article Type:**	Research Article
**Full Title:**	"Antimicrobial utilization in a paediatric intensive care unit in India: a step towards strengthening antimicrobial stewardship practices"
**Short Title:**	Antimicrobial Surveillance in PICU
**Corresponding Author:**	Nitin Rewaram Gaikwad, M.D.<br>All India Institute of Medical Sciences<br>Raipur, Chhattisgarh INDIA
**Keywords:**	Antimicrobial stewardship, PICU, Antimicrobial utilization, Active surveillance
**Abstract:**	Antimicrobials are frequently used in critically ill children admitted to the Paediatric Intensive Care Unit (PICU). The antimicrobial use data from Indian PICUs is scarce using standard metrics such as Days of therapy (DOT). This study aimed to determine the baseline trend of antimicrobial use in PICU of a tertiary care teaching hospital of Raipur district of Chhattisgarh, India using standard metrics for developing facility-wide antibiotic policy and strengthening the antimicrobial stewardship activities.<br>This active surveillance was conducted over a period of 18 months, from November 1, 2019, to March 21, 2021, in patients aged one month to 14 years who were admitted for ≥ 48 hours to the PICU at a tertiary care teaching hospital of Raipur District. Data on patient characteristics, antimicrobial indications, antimicrobial prescription information, and clinical outcomes were collected using pre-designed data abstraction forms. The descriptive statistic was used to represent the results. The antimicrobial consumption was expressed as DOT/1000 patient-days (PD).<br>A total of 216 patients were surveyed during the study period. The average number of antimicrobials prescribed per hospitalisation was 2.60 (range: 1 - 12), with 97.22% administered via parenteral route. Overall, DOT/1000-PD was 1318. The consumption of Watch Group antimicrobials was highest with 949 DOT/1000-PD, followed by Access (215) and Reserve Group (154), respectively. Ceftriaxone (208 DOT/1000 PD) was the most commonly prescribed antimicrobial agent, followed by Vancomycin (201), Meropenem (175), Piperacillin-Tazobactam (122) and Colistin (91). The patients who were escalated (28.24%) from empirical antimicrobial therapy had longer median PICU stay (8 days) compared those who were de-escalated (23.6%). Targeted therapy was given in 10.2% patients. The overall mortality rate was 14.35% and was higher (29.3%) in patients in whom empirical therapy was escalated compared to those who were de-escalated or continued.<br>The study established a benchmark for antimicrobials use in the PICU and highlighted priority areas for antimicrobial stewardship intervention to enhance de-escalation rates, enhance targeted therapy, and reduce the overuse of antimicrobials from the reserve group.
**Order of Authors:**	Madhusudan Prasad Singh, M.D.
	Nitin Rewaram Gaikwad, M.D.
	Yogendra Narayanrao Keche, M.D.
	Atul Jindal, M.D., D.M.
	Suryaprakash Dhaneria, M.D., D.M.
	Meenalotchini Prakash Gurunthalingam, M.D.
**Additional Information:**	
**Question**	**Response**
**Financial Disclosure**	The author(s) received no specific funding for this work.

Powered by Editorial Manager® and ProduXion Manager® from Aries Systems Corporation

Enter a financial disclosure statement that describes the sources of funding for the work included in this submission. Review the submission guidelines for detailed requirements. View published research articles from *PLOS ONE* for specific examples.<br><br>This statement is required for submission and **will appear in the published article** if the submission is accepted. Please make sure it is accurate.<br><br>**Funded studies**<br>Enter a statement with the following details:<br>• Initials of the authors who received each award<br>• Grant numbers awarded to each author<br>• The full name of each funder<br>• URL of each funder website<br>• Did the sponsors or funders play any role in the study design, data collection and analysis, decision to publish, or preparation of the manuscript?<br><br>Did you receive funding for this work?	
**Competing Interests**<br><br>Use the instructions below to enter a competing interest statement for this submission. On behalf of all authors, disclose any competing interests that could be perceived to bias this work—acknowledging all financial support and any other relevant financial or non-financial competing interests.<br><br>This statement is required for submission and **will appear in the published article** if the submission is accepted. Please make sure it is accurate and that any funding sources listed in your Funding Information later in the submission form are also declared in your Financial Disclosure statement.<br><br>View published research articles from	The authors have declared that no competing interests exist.

PLOS ONE for specific examples.<br><br>**NO authors have competing interests**<br><br>Enter: *The authors have declared that no competing interests exist.*<br><br>**Authors with competing interests**<br><br>Enter competing interest details beginning with this statement:<br><br>*I have read the journal's policy and the authors of this manuscript have the following competing interests: [insert competing interests here]*<br><br>* typeset	
**Ethics Statement**<br><br>Enter an ethics statement for this submission. This statement is required if the study involved:<br><br>• Human participants<br>• Human specimens or tissue<br>• Vertebrate animals or cephalopods<br>• Vertebrate embryos or tissues<br>• Field research<br><br>Write "N/A" if the submission does not require an ethics statement.<br><br>General guidance is provided below. Consult the submission guidelines for detailed instructions. **Make sure that all information entered here is included in the Methods section of the manuscript.**	Ethics Committee approval and the consent waiver was obtained before initiating the surveillance (AIIMSRPR/IEC/2019/331) from Institute Ethics Committee. The patient identity was not revealed at any stage during and after the study. The privacy and confidentiality have been maintained.

**Format for specific study types**<br><br>**Human Subject Research (involving human participants and/or tissue)**<br>• Give the name of the institutional review board or ethics committee that approved the study<br>• Include the approval number and/or a statement indicating approval of this research<br>• Indicate the form of consent obtained (written/oral) or the reason that consent was not obtained (e.g. the data were analyzed anonymously)<br><br>**Animal Research (involving vertebrate animals, embryos or tissues)**<br>• Provide the name of the Institutional Animal Care and Use Committee (IACUC) or other relevant ethics board that reviewed the study protocol, and indicate whether they approved this research or granted a formal waiver of ethical approval<br>• Include an approval number if one was obtained<br>• If the study involved *non-human primates*, add *additional details* about animal welfare and steps taken to ameliorate suffering<br>• If anesthesia, euthanasia, or any kind of animal sacrifice is part of the study, include briefly which substances and/or methods were applied<br><br>**Field Research**<br><br>Include the following details if this study involves the collection of plant, animal, or other materials from a natural setting:<br>• Field permit number<br>• Name of the institution or relevant body that granted permission	
**Data Availability**<br><br>Authors are required to make all data underlying the findings described fully available, without restriction, and from the time of publication. PLOS allows rare exceptions to address legal and ethical concerns. See the PLOS Data Policy and FAQ for detailed information.	Yes - all data are fully available without restriction

Powered by Editorial Manager® and ProduXion Manager® from Aries Systems Corporation

A Data Availability Statement describing where the data can be found is required at submission. Your answers to this question constitute the Data Availability Statement and **will be published in the article**, if accepted.<br><br>**Important:** Stating 'data available on request from the author' is not sufficient. If your data are only available upon request, select 'No' for the first question and explain your exceptional situation in the text box.<br><br>Do the authors confirm that all data underlying the findings described in their manuscript are fully available without restriction?	
**Describe where the data may be found in full sentences. If you are copying our sample text, replace any instances of XXX with the appropriate details.**<br><br>• If the data are **held or will be held in a public repository**, include URLs, accession numbers or DOIs. If this information will only be available after acceptance, indicate this by ticking the box below. For example: *All XXX files are available from the XXX database (accession number(s) XXX, XXX.).*<br>• If the data are all contained **within the manuscript and/or Supporting Information files**, enter the following: *All relevant data are within the manuscript and its Supporting Information files.*<br>• If neither of these applies but you are able to provide **details of access elsewhere**, with or without limitations, please do so. For example:<br><br>*Data cannot be shared publicly because of [XXX]. Data are available from the XXX Institutional Data Access / Ethics Committee (contact via XXX) for researchers who meet the criteria for access to confidential data.*<br><br>*The data underlying the results presented in the study are available from (include the name of the third party*	All relevant data are within the manuscript and its Supporting Information files.

and contact information or URL).<br>• This text is appropriate if the data are owned by a third party and authors do not have permission to share the data.<br><br>* typeset	
Additional data availability information:	Tick here if your circumstances are not covered by the questions above and you need the journal's help to make your data available.

Powered by Editorial Manager® and ProduXion Manager® from Aries Systems Corporation

Manuscript

Click here to access/download;Manuscript;Manuscript Plos One.docx

Antimicrobial utilization in a paediatric intensive care unit in India: a step towards strengthening antimicrobial stewardship practices"

Madhusudan Prasad Singh[1ψΣ], Nitin Rewaram Gaikwad[2ψΣ]*, Yogendra Narayanrao Keche[3Σ], Atul Jindal[4χ], Suryaprakash Dhaneria[5Σ], Meenalotchini Prakash Gurunthalingam[6χ]

[1] Department of Pharmacology, All India Institute of Medical Sciences, Raipur, Chhattisgarh, India

[2] Department of Pharmacology, All India Institute of Medical Sciences, Raipur, Chhattisgarh, India

[3] Department of Pharmacology, All India Institute of Medical Sciences, Raipur, Chhattisgarh, India

[4] Department of Paediatrics, All India Institute of Medical Sciences, Raipur, Chhattisgarh, India

[5] Department of Pharmacology, Ruxmaniben Deepchand Gardi Medical College, Ujjain, Madhya Pradesh, India

[6]Department of Pharmacology, All India Institute of Medical Sciences, Raipur, India

*Corresponding author

E-mail: nitingaikwad2707@aiimsraipur.edu.in (NRG)

[ψ]These authors contributed equally to the study concept, design, data analysis, and manuscript drafting.

[Σ]These authors contributed equally to acquisition of clinical data and data interpretation.

[χ]These authors contributed equally to acquisition of clinical data.

Abstract

Antimicrobials are frequently used in critically ill children admitted to the Paediatric Intensive Care Unit (PICU). The antimicrobial use data from Indian PICUs is scarce using standard metrics such as Days of therapy (DOT). This study aimed to determine the baseline trend of antimicrobial use in PICU of a tertiary care teaching hospital of Raipur district of Chhattisgarh, India using standard metrics for developing facility-wide antibiotic policy and strengthening the antimicrobial stewardship activities.

This active surveillance was conducted over a period of 18 months, from November 1, 2019, to March 21, 2021, in patients aged one month to 14 years who were admitted for ≥ 48 hours to the PICU at a tertiary care teaching hospital of Raipur District. Data on patient characteristics, antimicrobial indications, antimicrobial prescription information, and clinical outcomes were collected using pre-designed data abstraction forms. The descriptive statistic was used to represent the results. The antimicrobial consumption was expressed as DOT/1000 patient-days (PD).

A total of 216 patients were surveyed during the study period. The average number of antimicrobials prescribed per hospitalisation was 2.60 (range: 1 - 12), with 97.22% administered via parenteral route. Overall, DOT/1000-PD was 1318. The consumption of Watch Group antimicrobials was highest with 949 DOT/1000-PD, followed by Access (215) and Reserve Group (154), respectively. Ceftriaxone (208 DOT/1000 PD) was the most commonly prescribed antimicrobial agent, followed by Vancomycin (201), Meropenem (175), Piperacillin-Tazobactam (122) and Colistin (91). The patients who were escalated (28.24%) from empirical antimicrobial therapy had longer median PICU stay (8 days) compared those who were de-escalated (23.6%). Targeted therapy was given in 10.2% patients. The overall

mortality rate was 14.35% and was higher (29.3%) in patients in whom empirical therapy was escalated compared to those who were de-escalated or continued.

The study established a benchmark for antimicrobials use in the PICU and highlighted priority areas for antimicrobial stewardship intervention to enhance de-escalation rates, enhance targeted therapy, and reduce the overuse of antimicrobials from the reserve group.

Introduction

According to World Health Organization (WHO), irrational antibiotic use contributes to antimicrobial-resistance (AMR) worldwide. In South-East Asia, antibiotic misuse is common and contributes to antimicrobial resistance.[1] Broad-spectrum antimicrobials can cause infections due to multi-drug-resistant gram-negative bacilli and invasive candidiasis.[2,3] Multi-drug-resistant organism (MDRO) infections increase healthcare costs, length of stay, Intensive care unit (ICU) admission, morbidity, and mortality.[2,4-6] A WHO-funded community-based survey in India found that up to 53% of public sector primary care patients and 70% of private sector patients receive antibiotics for upper respiratory tract infection and acute diarrhoea in children and adults, indicating overuse and inappropriate use.[7]

Paediatric patients are special population, therefore inappropriate antimicrobial use that causes AMR is a major concern. Primary infections that require hospitalisation or secondary infections, including hospital-acquired infections (HAIs), are the most common issues in a Paediatric Intensive Care Unit (PICU).[1] Patients with severe sepsis, septic shock, and HAI need complete and appropriate antimicrobial therapy.[8-10]

Antimicrobials are widely used in PICUs (67%–97%) due to empirical antimicrobial therapy.[11] Empirical antimicrobial therapy often begins with local susceptibility. However,

signs, symptoms, and severity of the infection must be assessed and antimicrobial therapy narrowed for common ICU infections.[12] A lack of culture-sensitivity reports, a deteriorating patient's clinical status, or a failure to communicate between clinicians or residents on clinical rounds often lead clinicians to escalate and change antimicrobials during empirical antimicrobial therapy.[13]

Due to the massive AMR problem, focused and coordinated efforts to improve rational antimicrobial prescribing and active AMR surveillance through the Antimicrobial Stewardship Programme (AMSP) are needed. AMSP that has been successfully implemented in adults can also be implemented for paediatric populations. Studies by J R Paño-Pardo *et al*.[14], J Y Ting *et al*.[15], and Jef Willems *et al*.[16] have shown successful implementation of AMSP in paediatric settings. AMSP implementation requires baseline antimicrobial use and resistance patterns from our patient care areas. Antimicrobial use in children, especially in PICUs, is poorly documented. Upon reviewing the relevant literature, no Indian study has evaluated the use of antimicrobials in PICUs using standard metrics like Days of Therapy. Both antimicrobial use and clinical outcomes in PICU patients can provide insight. Thus, we designed this surveillance study to assess antimicrobial use and clinical outcomes in PICU-admitted patients receiving antimicrobial therapy.

Materials and methods

The active surveillance was conducted over 18 months (from November 1, 2019 to April 30, 2021) in Paediatric Intensive Care Unit (PICU) of All India Institute of Medical Sciences (AIIMS), Raipur, Chhattisgarh, India. Ethics Committee approval and the consent waiver was obtained before initiating the surveillance (AIIMSRPR/IEC/2019/331) from Institute Ethics Committee. The patient identity was not revealed at any stage during and after the study. The privacy and confidentiality have been maintained.

All the admitted patients aged one month to fourteen years in PICU, receiving at least one or more antibacterial agents with or without antifungal agents and whose PICU stay was ≥ 48 hours were included. Patients who received only antitubercular, antifungal, or antiviral agents, topical antimicrobial agents, or who were discharged, left against medical advice (LAMA), or who died within 48 hours of admission were excluded. Active surveillance was conducted in the PICU every day between 11:00 AM and 1:00 PM. The data were collected using a pre-designed structured data abstraction form from the patients' health records, nursing charts and microbiology laboratory reports. The information collected comprise of antimicrobial agents given by system route (oral or parenteral), microbiological samples sent and their culture sensitivity reports, laboratory investigations, and patient-days. The patient-days were counted every day at 11 AM during the surveillance period and it included patients admitted to the PICU on or before 11:00 AM on the day of data collection. (Fig 1)

Fig 1. Flow of the study participants

Data analysis

The data was analyzed for various parameters such as antimicrobial use expressed as DOT/1000 patient-days, WHO core prescribing indicators, number (%) of antimicrobials used empirically, number (%) of patients in who antimicrobial therapy de-escalated, escalated, or continued, number (%) of patients on targeted antimicrobials, number (%) of patients who received 'Access', Watch' or 'Reserve' antimicrobials and clinical outcomes like length of stay and mortality. To determine antimicrobial prescription compliance with the Essential Medicine List (EML), the list in use during the data collection period (National EML 2015 and WHO EML 2019) was applied.

Statistical analysis

Microsoft Excel 2013 were used to interpret the data for descriptive statistics such as median and interquartile range. Discrete data was expressed as counts or percentages. The antimicrobial consumption was expressed as DOT/1000 patient-days.

Results

The study included 216 of 511 screened patients who met inclusion and exclusion criteria. At the end of the surveillance period, the total number of patient -days was 2212. (Fig 1) The patients had a median age of four years (IQR: 0.62 – 9.00). . Table 1 depicts the demographic and clinical characteristics of the patients.

Table 1. Demographic Details and Clinical Characteristics.

Clinical Characteristics	Total (n=216)
Age in years, Median (IQR)	4.00 (0.62-9.00)
Gender, n (%)	
Male	118 (54.63%)
Female	98 (45.37%)
Management, n (%)	
Medical	178 (82.4%)
Surgical	38 (17.6%)
Mechanical Ventilation, n (%)	47 (21.8%)
Average number of ventilator days	7.68 days
Medical device in place, n (%)	56 (25.9%)
Central line	52 (24.07%)
Urinary Catheter	48 (22.22%)
Febrile on admission, n (%)	160 (74%)
Total Patient Days of PICU	2212
Reason for ICU admission (System-wise), n (%)	
Gastrointestinal	10 (4.62%)
Liver and Biliary	12 (5.55%)
Musculoskeletal	09 (4.16%)
Hereditary/Congenital disorders	18 (8.33%)
Respiratory	60 (27.8%)
Renal/Urinary	28 (12.9%)

CNS	58 (26.8%)
Cardiovascular system	26 (12.0%)
Oncology	27 (12.5%)
General	10 (4.62%)

The average number of antimicrobial agents prescribed per hospitalisation was 2.60 (range: 1-12), with 97.22% given parenterally. The average duration of prescribed antimicrobial treatment was 5.20 ±1.30 days. During study period, 86.2% of antimicrobials were prescribed by generic names. The percentage of prescribed antimicrobials compliant with National EML, India (NLEM 2015) and WHO EML 2019 (21st Edition) were 69.1% and 80.6%, respectively. The patients received 94.88% of the prescribed antimicrobial dosages. Patients who received at least one antimicrobial agent accounted for 25% of the total, while 46% patients received three or more antimicrobials. The overall DOT /1000 patient-days were 1318. (Table 2) Ceftriaxone (208 DOT/1000 patient-days) was the most commonly prescribed antimicrobial agent, followed by vancomycin (201), meropenem (175), piperacillin-tazobactam (122) and amikacin (101) (Table 2) In 91.7% patients, at least one antimicrobial agent from 'Watch' category was prescribed. (Table 2) Colistin (91 DOT/1000 patient-days), linezolid (33), and tigecycline (22) were the most frequently prescribed antimicrobials in the reserve group. (Table 2) The DOT/LOT ratio was 1.92, with a total DOT of 2911 days and LOT of 1520 days. The average antimicrobial treatment days for 'Reserve' category antimicrobials were higher (7.6 days) than for 'Watch' (4.9 days) and 'Access' (3.9 days) antimicrobials. (Table 2) Redundant double gram-negative and double anaerobic coverage antimicrobial coverage was given in 27.3% and 20.80% of patients, respectively. (Fig 2)

Fig 2. Data showing percentage of patients who received multiple drug coverage for Gram negative and anaerobic infections. Note: *Denominator: Total number of patients under surveillance (=216)

Table 2. Antimicrobial usage data (in terms of DOT/1000 patient days and according to WHO AWaRe Classification.

**1. Antimicrobial usage data- DOT / 1000 patient days**							
**Anti-mi[illegible]ial group**	**Antimicrobials**	**ATC Code**	**WHO AWaRe Classification**	**No. of patients (%)**	**Days of therapy (DOT)**	**DOT/1000 Patient days (% Consumption$^{\$}$)**	**Average antimicrobial treatment days**
	**All Antimicrobials**			216 (100%)	2911	1318	5.20
**Beta Lactams ± BLI**				104 (48.1%)	469	212 (16%)	4.5
	Ampicillin	J01CA01	Access group	13 (6%)	50	23 (1.7%)	3.8
	Ampicillin + Cloxacillin	J01CF02	Access group	14 (6.5%)	67	30 (2.3%)	4.8
	Amoxicillin	J01CA01	Access group	1 (0.46%)	3	1 (0.08%)	3
	Amoxicillin + Clavulanic Acid	J01CR02	Access group	19 (8.8%)	64	29 (2.2%)	3.4
	Cloxacillin	J01CF02	Access group	3 (1.4%)	15	7 (0.5%)	5
	Piperacillin + Tazobactam	J01CR05	Watch group	54 (25%)	270	122 (9.3%)	5
**3rd Gen. Cephalosporins**				132 (61.11%)	589	267 (20.3%)	4.5
	Ceftriaxone	J01DD01	Watch group	96 (44.4%)	459	208 (15.8%)	4.8
	Cefotaxime	J01DD01	Watch group	26 (12%)	100	45 (3.4%)	3.8
	Ceftazidime	J01DD02	Watch group	6 (2.6%)	13	6 (0.45%)	2.2
	Cefpodoxime	J01DD13	Watch group	2 (0.9%)	9	4 (0.3%)	4.5
	Cefixime	J01DD08	Watch group	1 (0.4%)	2	1 (0.08%)	2
	Cefoperazone	J01DD12	Watch group	1 (0.4%)	6	3 (0.2%)	6
Carbapenems	Meropenem	J01DH02	Watch group	59 (27.3%)	386	175 (13.3%)	6.5
Monobactam	Aztreonam	J01DF01	Reserve group	2 (0.9%)	9	4 (0.3%)	4.5
Polymyxins	Colistin	J01XB01	Reserve group	27 (12.5%)	201	91 (6.9%)	7.4
**Glycopeptides**				81 (37.5%)	515	233 (17.7%)	6.4
	Vancomycin	J01XA01	Watch group	72 (33.3%)	444	201 (15.3%)	6.2
	Teicoplanin	J01XA02	Watch group	9 (4.16%)	71	32 (2.4%)	7.9
Oxazolidinones	Linezolid	J01XX08	Reserve group	9 (4.16%)	74	33 (2.5%)	7.4
Glycylcycline	Tigecycline	J01AA12	Reserve group	5 (2.3%)	49	22 (1.7%)	9.8
**Fluoroquinolones**				3 (1.39%)	7	3 (0.2%)	2.3
	Levofloxacin	J01MA12	Watch group	2 (0.9%)	6	3 (0.2%)	3
	Ciprofloxacin	J01MA02	Watch group	1 (0.46%)	1	0.45 (0.03%)	1
Phosphonic acid derivative	Fosfomycin (IV)	J01XX01	Reserve group	1 (0.46%)	9	4 (0.3%)	9
**Aminoglycosides**				76 (35.2%)	264	119 (9%)	3.5
	Amikacin	J01GB06	Access group	65 (30%)	223	101 (7.7%)	3.4

	Gentamicin	J01GB03	Access group	11 (5.09%)	41	19 (1.4%)	3.7
**Macrolides**	Azithromycin	J01FA10	Watch group	24 (11.1%)	126	57 (4.3%)	5.2
**Tetracyclines**	Doxycycline	J01AA02	Access group	3 (1.4%)	12	5 (0.4%)	4
**Rifamycin**				5 (2.3%)	32	15 (1.1%)	6.4
	Rifampicin	J01AM02	Watch group	2 (0.9%)	8	4 (0.3%)	4
	Rifaximin	A07AA11	Watch group	3 (1.4%)	24	11 (0.8%)	8
**Lincosamide**	Clindamycin	J01FF01	Access group	9 (4.2%)	37	17 (1.3%)	4.1
**Sulfonamides**	Sulfamethoxazole and trimethoprim	J01EE01	Access group	14 (6.48%)	88	40 (3%)	6.3
**Nitroimidazole**	Metronidazole	J01XD01	Access group	10 (4.6%)	34	15 (1.1%)	3.4
**Nitro-methylene-diamino imidazolidinediones**	Nitrofurantoin	J01XE51	Access group	1 (0.46%)	10	5 (0.4%)	10
**2. Antimicrobial usage as per WHO AWaRe Classification**							
**AWaRe Class**		**Days of Therapy/1000 Patient days (% Consumption$)**		**Average antimicrobial treatment days**		**Percentage of patients receiving antimicrobials***	
Access group		215 (16.32%)		3.9		115 (53.2%)	
Watch group		949 (72.00%)		4.9		198 (91.7%)	
Reserve group		154 (11.68%)		7.6		29 (13.4%)	
Total		1318		-		-	

Note: $Numerator: DOT/1000PD, $Denominator: Total DOT/1000PD (=1318), *Numerator: Number of patients receiving at least one antimicrobial agent from Access/Watch/Reserve group, *Denominator: Total number of patients receiving antimicrobials (=216)

Fig S3 depicts empirical antimicrobial use. Ceftriaxone was empirically prescribed to 98.90% of patients, followed by vancomycin (82.20%) and amikacin (77.60%). The culture sensitivity report determined targeted antimicrobial therapy for 47.8% of culture positive isolates. (Table S1) Empirical antimicrobial therapy was de-escalated in 23.6% of patients, 17.6% of whom had microbiological culture-sensitivity reports. Based on culture-sensitivity, 11.1% of patients had empirical antimicrobial therapy de-escalated, escalated, or continued. (Table 3) The median length of PICU stay for the patients who were de-escalated, escalated, and continued antimicrobial therapy was 7 days (IQR: 5 – 12.25 days), 8 days (IQR: 5 – 12 days), and 4 days (IQR: 3 – 6.75 days), respectively. (Fig 3)

Fig 3. Box and Whisker plot for PICU stay by number of patients in whom antimicrobial therapy was escalated, de-escalated and continue. Note : Category 1= No. of De-escalated

Patients, Category 2= No. of Escalated Patients, Category 3= No. of Continuation therapy Patients.

Table 3. De-Escalation, escalation and continuation of antimicrobial therapy

S.N.		Parameter	Value
**1A**		Percentage of patients in whom antimicrobial therapy was de-escalated, n (%) *	51 (23.6%)
	**i**	Withdrawal of ≥1 antimicrobial agents	51 (23.6%)
	**ii**	Withdrawal of at least one antimicrobial agent + Addition of narrow spectrum antimicrobial agent/s	13 (6.01%)
	**iii**	Stopping empirical therapy and switching to narrow spectrum	9 (4.16%)
**1B**		Percentage of patients in whom antimicrobial therapy was de-escalated based on culture sensitivity report, n (%) [$]	9 (17.6%)
**2A**		Percentage of patients in whom antimicrobial therapy was escalated, n (%) *	61 (28.24%)
	**i**	Addition of ≥1 antimicrobial agent/s to empirical therapy	61 (28.24%)
	**ii**	Switching from narrow spectrum to broad spectrum	39 (18.05%)
	**iii**	Withdrawal of ≥1 antimicrobial agent/s from empirical therapy BUT Addition of ≥1 broad spectrum antimicrobial agent/s	31 (14.35%)
**2B**		Percentage of patients in whom antimicrobial therapy was escalated based on culture sensitivity report, n (%) [$]	6 (9.8%)
**3A**		Percentage of patients in whom therapy was continued, n (%) *	104 (48.15%)
**3B**		Percentage of patients in whom therapy was continued based on culture sensitivity report, n (%) [$]	9 (8.7%)
**4**		Total Percentage of patients in whom therapy was De-escalated/escalated/continued based on culture sensitivity report, n (%) [#]	24 (11.1%)

Note: Denominator: * Total number of patients under surveillance (=216), [$]Total number of patients in whom therapy was De-escalated/escalated or continued (De-escalated=51, escalated=61, Continued=104), [#] Total number of patients under surveillance (=216)

At least one sample was sent for microbiological culture-sensitivity testing for 74.5% of patients. However, this testing was only done before empirical antimicrobial therapy for 46.8% of patients. The culture-positivity yield was 16.7% and the targeted therapy was administered for 47.8% of culture-positive isolates. (Table 3) The incidence rate of hospital-acquired MRSA, MDRO infection and multi-drug-resistant Gram-negative bacteria (MDR-GNB) infection per 1000 patient-days was 0.9, 6.3 and 4.1, respectively.(Table 4) The most

common isolated organism was *Escherichia coli* – 10 (21.7%) followed by *Acinetobacter* – 9 (19.6%) *and Coagulase-negative Staphylococcus (CoNS)* – 7 (15.2%). (Table S1, Figs S2,S3)

Of 216 patients, 69% received antimicrobial therapy for ≤7 days, 24% for 8-14 days, and 7% for ≥15 days. The Median PICU stay of study population was six (06) days (IQR:4-9). (Table 4) The total mortality rate was 14.35% (Table 4), and it was higher (29.3%) in patients whose empirical therapy was escalated than in those whose empirical therapy was de-escalated or continued. (Fig 4) After 48 hours of antimicrobials, 55% of 76% febrile admissions became afebrile. Seven (3.24%) of 216 patients under surveillance were readmitted within 7 days of discharge/transfer. (Table 4)

Fig 4. Relationship between mortality rate and escalation, de- escalation and continuation of empirical therapy.

Table 4. Clinical and Microbiological Indicators.

**I.**	**Antimicrobial treatment and PICU Stay**	
**Length of antimicrobial therapy**	≤7 days	148 (69%)
	8–14 days	52 (24%)
	≥15 days	16 (7%)
**Median PICU Stay (IQR) in days**	-	6 (4-9)
**PICU Stay (Days)**	2–10 days	170 (79%)
	11–20 days	39 (18%)
	≥21 days	7 (3%)
**II.**	**Clinical outcome indicators**	
**S.N.**	**Parameter**	**Percentage**
**1.**	Mortality rate (n) *	31 (14.35%)
**2.**	Percentage of patients who became afebrile within 48 hours of PICU admission[+]	88 (55%)
**3.**	Readmission rate within 7 days of discharge/transfer out *	7 (3.24%)
**III.**	**Microbiological Indicators**	
**1.**	Percentage of patients in whom sample was sent for culture sensitivity test *	161 (74.5%)
**2.**	Culture positivity rate (Microbiological yield) [#]	46 (16.9%)
**3.**	Number of samples in which multi drug resistant organisms (MDRO) was isolated, n (%) [$]	30 (18.6%)
**4.**	Incidence Rate of Hospital- acquired MRSA infection per 1000 patient days[@]	0.9
**5.**	Incidence Rate of Total Hospital- acquired Multi drug resistant organism (MDRO) infection per 1000 patient days[@]	6.3

**6.**	Incidence Rate of Hospital- acquired Multi drug resistant gram-negative bacteria (MDR-GNB) infection per 1000 patient days@	4.1

Note: Percentage rounded to nearest zero, * Denominator: Total number of patients under surveillance (=216), + Denominator: Total number of patients who were febrile on admission (=160), # Denominator: total number of samples sent (=272), $Denominator: total number of patients in whom sample was sent for culture sensitivity test (=161), @Denominator: Total patient days (=2212)

Discussion

Antimicrobials are crucial to infectious disease management and prevention, especially in children. Beta-lactams with or without beta-lactamase inhibitors, carbapenems, glycopeptides, aminoglycosides, and polymyxins were consumed more in the PICU, according to this surveillance study. 'Watch' category antimicrobials like ceftriaxone, vancomycin, meropenem, piperacillin-tazobactam, and colistin accounted for 60% of use. Although 'Reserve' group antimicrobials have lower consumption, their longer average treatment days are notable. The findings emphasise the need for continuous monitoring and effective stewardship of 'Watch' and 'Reserve' group antimicrobials. In contrast to the observation by Panditrao *et al.* (2021)[17], our surveillance data indicated higher consumption of ‘Watch’ group antimicrobials (91.7% vs 80.56%), prominent being third-generation cephalosporins. Our data is limited to single centre and paediatric patients, unlike Panditrao *et al.* observations from various treatment facilities and age groups; however, the widespread use of these antimicrobials raises their resistance potential, requiring periodic surveys. Most studies report paediatric antimicrobial usage ranging from 44% to 97%, indicating high rates.[18-21] Our data suggests high empirical use of colistin (39.30%), linezolid (30%), tigecycline (20%) (Fig S3). The use of ‘reserve’ antimicrobials is anticipated in critical care setting. Nonetheless, over-prescription is alarming and may be influenced by prior antimicrobial use, low microbiological

yield, critical illness, risk of MDRO infections, and non-improvement or clinical deterioration.[22]

The prevalence of high antimicrobial use in paediatric patients in Southeast Asian countries is comparable to India and Western countries. In a study from Pakistan, Abbas *et al.*[11] (2016) found that 100% of PICU-admitted patients received at least one antimicrobial agent for prophylaxis, therapeutic, or empirical purposes. Similarly, Boone *et al.*[23] (2020) noted that antimicrobials were prescribed in 73% of infants admitted to hospitals in Bangladesh.

Double Gram-negative and anaerobic coverage (DAC) was another finding in our surveillance. Song *et al.*[24] (2015) found that 26.8% of patients received unnecessary double anaerobic coverage for over three days, compared to 20.8% in our study. In contrast, Rattanaumpawan *et al.*[25] reported a cumulative incidence of 2.8% (22/781) and 1.9% (19/1002) DAC before and after AMSP changes.

Escalation of antimicrobial therapy correlated with higher mortality and extended PICU stays. Additionally, prescribing ≥ 3 antimicrobials and prolonged therapy were associated with longer PICU stays (Figs S1 and S2). Compliance with generic names and Essential Medicine List was suboptimal. Limited availability of cloxacillin, which is more effective against MSSA, leads to irrational use of amoxicillin-clavulanic acid, vancomycin, and linezolid. Only life-threatening beta-lactam allergies warrant vancomycin use.[26] Cloxacillin consumption was low (7 DOT per 1000-patient days) and only half of MSSA patients received targeted therapy during our study.

Regular review of hospital formulary and alignment with the National Essential Medicine List can ensure the availability of narrow-spectrum antimicrobials for targeted drug therapy. The low culture-positivity yield in a critical care setting can be attributed to several factors. These include the lack of sampling before initiating antimicrobial therapy, improper

sample collection techniques, drawing cultures from lines used for administering antimicrobials, inadequate blood volume for testing in pediatric patients, and issues with transportation, incubation, handling, and culture systems.[27-31] The culture-positivity yield was 16.9% in our study. Being a referral center, the patients received from other hospital often had received high-end antimicrobials. This low rate of culture-positivity has resulted in a reduced rate of targeted therapy and de-escalation. Urgent implementation of stewardship interventions, such as promoting a culture of sending sample for culture-sensitivity – “culture of culture" and applying pharmacological concepts in prescribing, is necessary to improve these rates. Educational programs and feedback sessions for healthcare professionals should also be implemented to support these interventions. The primary strength of this study is its 18-month longitudinal study design which provided a comprehensive assessment of antimicrobial consumption and clinical outcomes in a PICU using standard surveillance methodology and WHO-recommended indicators. However, challenges were encountered during the study, such as a smaller sample size due to the conversion of the PICU to a COVID ICU and delays in microbiological reports. The study primarily relied on microbiological data from patient files during the surveillance period. The appropriateness of antimicrobials could only be determined in a few cases due to low culture-positivity yield. Other indicators were used to assess appropriateness, but patient-specific clinical characteristics were not evaluated. The study suggests that more efforts are needed to improve rational prescribing practices. The challenges faced during data analysis for this study included the lack of comparable data from Indian PICUs and a scarcity of high-quality studies using standard antimicrobial consumption metrics. Categorizing patients for de-escalation and escalation was also challenging, especially when both occurred in the same patient on different occasions, requiring an agreement to address this issue. The Indian Academy of Pediatrics and ICMR collaborated in 2014 to combat antimicrobial resistance in children.[32] The CDC's Antibiotic Use (AU) Option in National

Healthcare Safety Network (NHSN) offers an automated model for monthly DOT data collection.[33] Aligning with ICMR's initiative, a national network in India can provide baseline data for stewardship evaluation.

Conclusion

In conclusion, this surveillance study has provided valuable baseline data on antimicrobial usage in a PICU of a tertiary care hospital in Chhattisgarh State. This data can serve as a benchmark for other hospitals in the state and in India to compare their antimicrobial usage in pre-and post-AMSP intervention period. In addition, this surveillance identified check-points for stewardship interventions, such as reducing double anaerobic coverage and improving culture-positivity yield, facilitating de-escalation and targeted therapy, reducing overuse of reserve group antimicrobials, impacting clinical outcomes like mortality and length of stay.

Acknowledgement

-None

References

1. World Health Organization. Regional strategy on prevention and containment of antimicrobial resistance. WHO Regional Office for South-East Asia New Delhi:WHO;2010-2015.37.SEA-HLM:407. Available from: http://www.searo.who.int/entity/antimicrobial_resistance/BCT_hlm-407.pdf. (Accessed on 2021 Oct 4).
2. Singh N, Patel KM, Léger MM, Short B, Sprague BM, Kalu N, Campos JM. Risk of resistant infections with Enterobacteriaceae in hospitalized neonates. 2002;21:1029-33.

3. Cotten CM, McDonald S, Stoll B, Goldberg RN, Poole K, Benjamin DK. The association of third-generation cephalosporin use and invasive candidiasis in extremely low birth-weight infants. *Pediatr Infect Dis J*.2006 Aug 1; 118(2):717-22.

4. Mazzeo F, Capuano A, Avolio A, Filippelli A, Rossi F. Hospital-based intensive monitoring of antibiotic-induced adverse events in a university hospital. *Pharmacol Res*. 2005 Mar 1; 51(3):269-74.

5. McNeeley DF, Saint-Louis F, Noel GJ. Neonatal enterococcal bacteremia: an increasingly frequent event with potentially untreatable pathogens. *Pediatr Infect Dis J*. 1996 Sep 1; 15(9):800-5.

6. Stone PW, Gupta A, Loughrey M, Della-Latta P, Cimiotti J, Larson E, *et al*. Attributable costs and length of stay of an extended-spectrum beta-lactamase-producing Klebsiella pneumoniae outbreak in a neonatal intensive care unit. *Infect Control Hosp Epidemiol*.2003 Aug; 24(8):601-6.

7. Kotwani A, Holloway K. Antibiotic prescribing practice for acute, uncomplicated respiratory tract infections in primary care settings in New Delhi, India. *Trop Med Int Health*. 2014 Jul; 19(7):761–8.

8. CDC. Core Elements of Hospital Antibiotic Stewardship Programs. Atlanta, GA: US Department of Health and Human Services, CDC; 2014. Available from: https://www.cdc.gov/antibiotic-use/core-elements/hospital.html. Accessed on 2021 Oct 4.

9. Barlam TF, Cosgrove SE, Abbo LM, MacDougall C, Schuetz AN, Septimus EJ, *et al.* Executive summary: implementing an antimicrobial stewardship program: guidelines by the infectious diseases' society of America and the society for healthcare epidemiology of America. Clin Infect Dis. 2016 May 15; 62(10):1197-1202.

10. Rhodes A, Evans LE, Alhazzani W, Levy MM, Antonelli M, Ferrer R *et al.* Surviving Sepsis Campaign: International Guidelines for Management of Sepsis and Septic Shock. Intensive Care Med.2017 Mar; 45(3):486-552.

11. Abbas Q, Haq AU, Kumar R, Ali SA, Hussain K, Shakoor S. Evaluation of antibiotic use in pediatric intensive care unit of a developing country. Indian J Crit Care Med. 2016 May; 20(5):291-94.

12. Rizk NA, Kanafani ZA, Tabaja HZ, Kanj SS. Extended infusion of beta-lactam antibiotics: optimizing therapy in critically-ill patients in the era of antimicrobial resistance. Expert Rev Anti-Infect Ther. 2017 Jul 3; 15(7):645-52.

13. Brogan TV, Thurm C, Hersh AL, Gerber JS, Smith MJ, Shah SS, *et al.* Variability in Antibiotic Use Across PICUs. Pediatr Crit Care Med. 2018 Jun 1;19(6):519–27.

14. Paño-Pardo JR, Schüffelmann-Gutiérrez C, Escosa-García L, Laplaza-González M, Moreno-Ramos F, Gómez-Gil R, *et al.* Opportunities to improve antimicrobial use in paediatric intensive care units: a nationwide survey in Spain. Clin Microbiol Infect. 2016 Feb 1; 22(2):171-77.

15. Ting JY, Paquette V, Ng K, Lisonkova S, Hait V, Shivanada S *et al*. Reduction of Inappropriate Antimicrobial Prescriptions in a Tertiary Neonatal Intensive Care Unit After Antimicrobial Stewardship Care Bundle Implementation. Pediatr Infect Dis J. 2019 Jan 1; 38(1):54-9.

16. Willems J, Hermans E, Schelstraete P, Depuydt P, De Cock P *et al.* Optimizing the Use of Antibiotic Agents in the Pediatric Intensive Care Unit: A Narrative Review. Pediatr Drugs.2021Jan;23(1):39-53.

17. Panditrao AM, Shafiq N, Chatterjee S, Pathak A, Trivedi N, Sadasivam B, *et al.* ASPIRE (‘Antimicrobial Stewardship Programme In Resource-limited Environment’) Study Group. A multicentre point prevalence survey (PPS) of antimicrobial use amongst admitted patients in tertiary care centres in India. J Antimicrob Chemother.2021 Mar 12;76(4):1094-1101.
18. Mali NB, Deshpande SP, Tullu MS, Deshmukh CT, Gogtay NJ, Thatte UM. A prospective antibacterial utilization study in pediatric intensive care unit of a tertiary referral centre. Indian J Crit Care Med.2018 Jun; 22(6):422-6.
19. Sharma M, Damlin A, Pathak A, Stålsby Lundborg C. Antibiotic prescribing among pediatric inpatients with potential infections in two private sector hospitals in Central India. PLoS One. 2015 Nov 5; 10(11):1-12.
20. Baidya S, Hazra A, Datta S, Das AK. A study of antimicrobial use in children admitted to pediatric medicine ward of a tertiary care hospital. Indian J Pharmacol.2017 Jan; 49(1):10-15.
21. Gandra S, Singh SK, Jinka DR, Kanithi R, Chikkappa AK, Sharma A, *et al.* Point prevalence surveys of antimicrobial use among hospitalized children in six hospitals in India in 2016. Antibiotics.2017 Sep 13; 6(3):19.
22. Llor C, Bjerrum L. Antimicrobial resistance: risk associated with antibiotic overuse and initiatives to reduce the problem. Ther Adv Drug Saf. 2014 Dec; 5(6):229-41.
23. Boone K, Morris SK, Doshi S, Black J, Mohsin M, Ahmed T, *et al.* Antimicrobial prescribing during Infant hospital admissions in a birth cohort in Dhaka, Bangladesh. J Trop Pediatr.2021 Jul 2; 67(3):fmaa093.
24. Song YJ, Kim M, Huh S, Lee J, Lee E, Song KH, *et al.* Impact of an Antimicrobial Stewardship Program on Unnecessary Double Anaerobic Coverage Prescription. Infect Chemother. 2015 Jun 1; 47(2):111-6.

25. Rattanaumpawan P, Morales KH, Binkley S, Synnestvedt M, Weiner MG, Gasink LB, *et al.* Impact of antimicrobial stewardship programme changes on unnecessary double anaerobic coverage therapy. J Antimicrob Chemother. 2011 Nov 1;66(11):2655-8.
26. Conan MacDougall. Protein Synthesis Inhibitors and Miscellaneous Antibacterial Agents. In: Brunton L, Hilal-Dandan R, Knollmann B. (eds.) Goodman & Gilman's the pharmacological basis of therapeutics.13th edition. New York (NY): McGraw-Hill Education; 2018.p1049-1065.
27. Kern SE. Challenges in conducting clinical trials in children: Approaches for improving performance. Expert Rev Clin Pharmacol. 2009 Nov 1; 2(6):609-17.
28. Arya VK. Basics of fluid and blood transfusion therapy in paediatric surgical patients. Indian J Anaesth. 2012 Sep; 56(5):454-62.
29. Ombelet S, Barbé B, Affolabi D, Ronat JB, Lompo P, Lunguya O, *et al*. Best practices of blood cultures in low- and middle income countries. Front Med. 2019 Jun 18; 6:131.
30. WHO guidelines on drawing blood: best practices in phlebotomy. Available from: https://www.euro.who.int/__data/assets/pdf_file/0005/268790/WHO-guidelines-on-drawing-blood-best-practices-in-phlebotomy-Eng. (Accessed on 2021 Sept 29).
31. Haddadin Y, Annamaraju P, Regunath H. Central line associated blood stream infections (CLABSI). StatPearls. Available from: https://www.ncbi.nlm.nih.gov/books/NBK430891/?report=classic. (Accessed on 2021 Oct 4).
32. Bedi N, Gupta P. Antimicrobial stewardship in pediatrics: An Indian perspective. Indian pediatr. 2016 Apr 1; 53(4):293-8.
33. Aizawa Y, Suwa J, Higuchi H, Fukuoka K, Furuichi M, Kaneko T, *et al.* Antimicrobial Stewardship Program in a Pediatric Intensive Care Unit. J Pediatr Infect Dis Soc. 2018 Aug 17; 7(3):156-9.

Supporting information

S1 Table. Isolated pathogens and targeted antimicrobial therapy. Note: B = Blood, U = Urine, TBA = Tracheobronchial aspirate, P = Pus, S = Sputum, ST = Stool. * Denominator taken is total number of isolates (=46).

S1 Fig. Box and Whisker plot for PICU stay by number of antimicrobial agents prescribed. Note: Category 1= Number of patients who were prescribed 1 antimicrobial agent, Category 2 = Number of patients who were prescribed 2 antimicrobial agents, Category 3 = Number of patients who were prescribed ≥3 antimicrobial agents

S2 Fig. Box and Whisker plot for PICU stay by antimicrobial treatment days. Note: Category 1= Antimicrobial treatment days ≤7 days, Category 2= Antimicrobial treatment days 8-14 days, Category 3= Antimicrobial treatment days ≥15 days

S3 Fig. Usage of antimicrobial agents as empirical therapy. Note: *Denominator for ‘overall percentage’: Total number of patients under surveillance (=216) and for ‘percentage of patients prescribed as empirical therapy’: Total number of patients who received respective antimicrobial agent

S4 Fig. Stacked bar chart showing Gram positive organisms isolated and their sensitivity pattern for the antimicrobials in percentage. NOTE: R= RESISTANT, S= SENSITIVE, I= INTERMEDIATE, P= PENICILLIN, OX= OXACILLIN, AM= AMPICILLIN, CRO= CEFTRIAXONE, FEP= CEFEPIME, COT= COTRIMOXAZOLE, VA= VANCOMYCIN, E=ERYTHROMYCIN, DO= DOXYCYCLINE, GM= GENTAMICIN, CM= CLINDAMICIN, NX= NORFLOXACIN, FM= NITROFURANTOIN

S5 Fig. Stacked bar chart showing Gram negative organisms isolated and their sensitivity pattern for the antimicrobials in percentage. NOTE: R= RESISTANT, S= SENSITIVE, I= INTERMEDIATE, P= PENICILLIN, AM= AMPICILLIN, AMC= AMOXICILLIN+CLAVULANATE, TZP= PIPERACILLIN+TAZOBACTAM, CRO= CEFTRIAXONE, CTX= CEFOTAXIME, CAZ= CEFTAZIDIME, CXM= CEFUROXIME, CZ=CEFAZOLIN, CFM=CEFEXIME, FEP= CEFEPIME, COT= COTRIMOXAZOLE, VA= VANCOMYCIN, E= ERYTHROMYCIN, IPM= IMIPENEM, MEM= MEROPENEM, DO= DOXYCYCLINE, AN= AMIKACIN, GM= GENTAMICIN, TM= TOBRAMYCIN, CL= COLISTIN, ATM= AZTREONAM, CM= CLINDAMICIN, LVX= LEVOFLOXACIN, NX= NORFLOXACIN, FM= NITROFURANTOIN

Figure Click here to access/download;Figure;Figures.docx

Figures

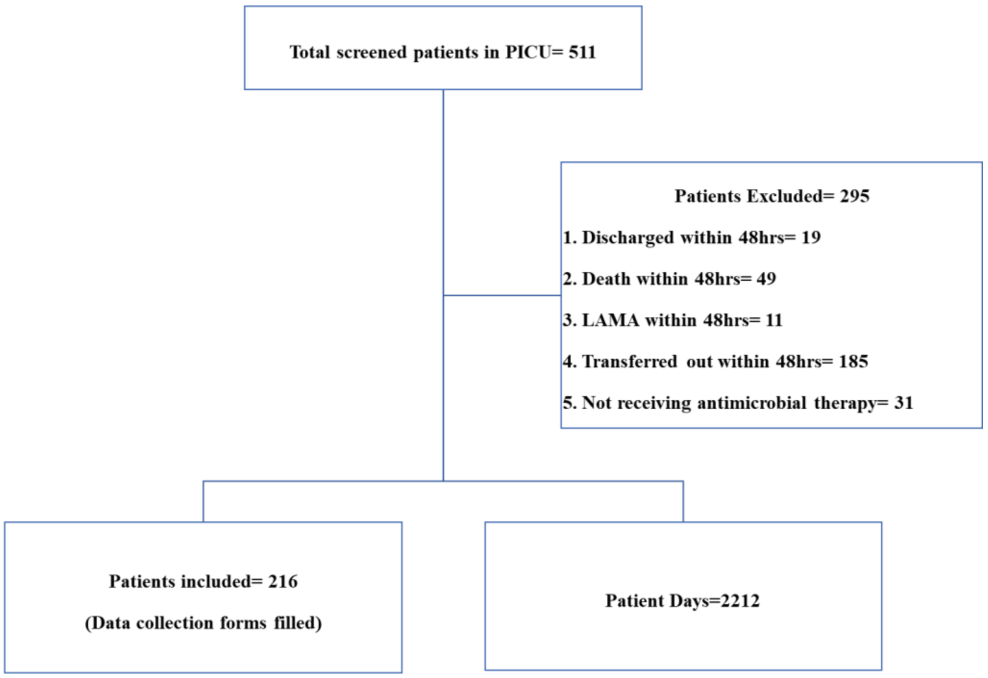

Figure 1. Flow of the study participants

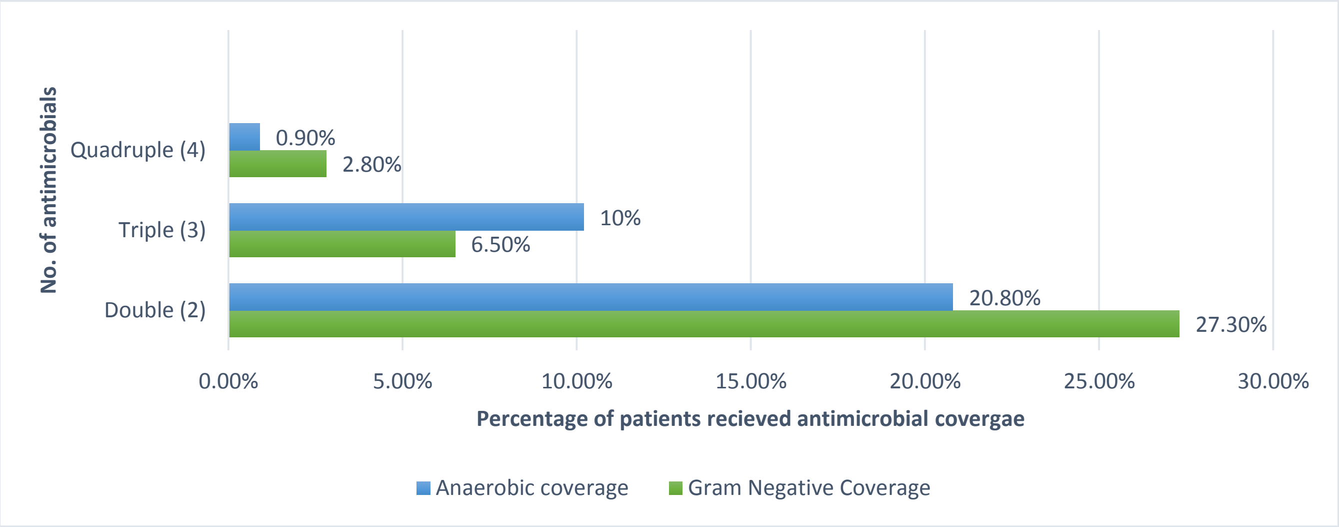

Note: *Denominator: Total number of patients under surveillance (=216)

Figure 2. Data showing percentage of patients who received multiple drug coverage for Gram negative and anaerobic infections

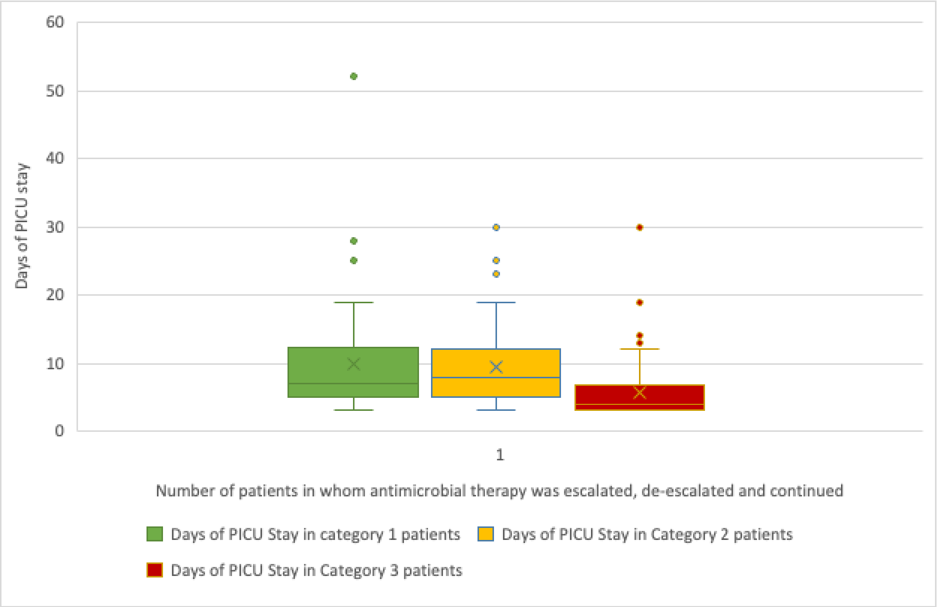

Note: Category 1= No. of De-escalated Patients, Category 2= No. of Escalated Patients, Category 3= No. of Continuation therapy Patients

Figure 3. Box and Whisker plot for PICU stay by number of patients in whom antimicrobial therapy was escalated, de-escalated and continue

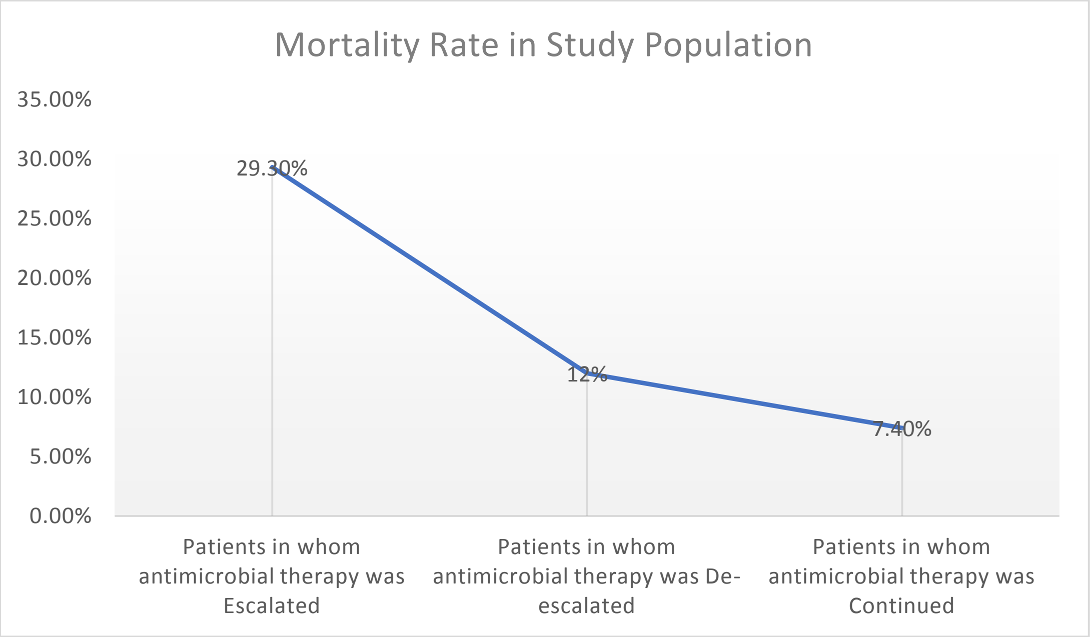

Figure 4. Relationship between mortality rate and escalation, de- escalation and continuation of empirical therapy

Click here to access/download
Supporting Information
Supporting Information.docx
