# Supplementary material for: “Antimicrobial utilization in a paediatric intensive care unit in India: A step towards strengthening antimicrobial stewardship practices"
Source: PLoS One. 2024 Sep 19;19(9):e0310515. doi: 10.1371/journal.pone.0310515 (PMC11412675; doi:10.1371/journal.pone.0310515)
Supplement: Attachment — Submitted filename: Rebuttal Letter.docx [file pone.0310515.s010.docx]

**Rebuttal Letter**

Dear Editor,

We appreciate the opportunity to submit our manuscript titled "Antimicrobial utilization in a paediatric intensive care unit in India: a step towards strengthening antimicrobial stewardship practices" to PLOS One. We thank the reviewers for their insightful comments and suggestions. We have carefully considered each of the reviewers' comments and have addressed them accordingly in this rebuttal letter.

| **S. No.** | **Reviewer Comments** | **Response** |
| --- | --- | --- |
| Reviewer #1 | The study provides valuable insights into antimicrobial use in a pediatric ICU setting and underscores the importance of antimicrobial stewardship.  However, I would like to make some criticisms.   - Excluding patients who were discharged, left against medical advice, or died within 48 hours might skew the understanding of antimicrobial use patterns and outcomes in critically ill patients. | In response to your concern about excluding patients who were discharged, left against medical advice, or died within 48 hours, we would like to provide clarification on our rationale for this decision. Our primary objective was to observe the antimicrobial usage patterns in patients admitted to the PICU using standard metrics. The inclusion of data from patients who received antimicrobials for less than 48 hours may not provide an accurate representation of antimicrobial usage among this population. The culture-sensitivity reports are available after 48 hours of sending microbiological samples at the initiation of empiric therapy. In addition, de-escalation/escalation of antimicrobial therapy is done based on culture-sensitivity report. These observations can be done only in those patients who receive antimicrobial therapy more than 48 hours. Therefore, in contrast to your comment, the inclusion of patients who received antimicrobial therapy and were discharged, left against medical advice, or died within 48 hours could have potentially skewed the results. Therefore, we defined these exclusion criteria at the outset of the study to ensure the integrity of our analysis.  We understand the importance of capturing a comprehensive picture of antimicrobial use in critically ill patients and appreciate your feedback on this matter. However, we believe that our approach aligns with the objectives of our study and provides valuable insights into antimicrobial stewardship practices within the pediatric ICU setting. |
|  | - The study primarily used descriptive statistics for analysis. Incorporating inferential statistical methods could provide deeper insights into the significance of the findings and the relationship between antimicrobial use and clinical outcomes. | We acknowledge your suggestion regarding the incorporation of inferential statistical methods to provide deeper insights into the significance of our findings and the relationship between antimicrobial use and clinical outcomes. However, after careful consideration, we have chosen to primarily utilize descriptive statistics for our analysis.  Our decision to focus on descriptive statistics was intentional and aligned with the objectives of our study.  Moreover, given the nature of our data and study design, we believe that descriptive statistics effectively convey the key findings and trends observed in our study population. |
|  | - The study notes a high rate of empirical use of 'Reserve' antimicrobials, which is alarming. However, it does not delve deeply into the reasons behind this trend (severe infections? serious patients?) or suggest specific strategies for reducing their use. | The high use of 'Reserve' antimicrobials in our critical care setting is primarily due to the severity of infections among our patients, as evidenced by the fact that 21.8% of patients were on mechanical ventilation. Such patients often require reserve group antimicrobials. As a tertiary care center, we also receive many patients referred from other centers who have already been treated with higher antimicrobials, like carbapenems.  However, our primary objective was not to identify risk factors but to establish a benchmark for the use of reserve antimicrobials at our institute and to compare this with other institutes. Any reduction in the use of these antimicrobials can serve as a good indicator of improved practices. While assessing the severity of infections and correlating this with the use of reserve antimicrobials was not within the scope of our current study, this is an important area that could be addressed in future research. Nevertheless, we emphasize that the empirical use of reserve antimicrobials is unwarranted and should be discouraged. |
|  | o The low culture-positivity yield is acknowledged but not sufficiently analyzed. | The culture positivity yield has been discussed at length in our discussion section, where we explore probable reasons for this observation. However, this finding has paved the way for further research aimed at identifying contributing factors. A quality improvement study could be conducted to examine elements such as transportation that may affect culture positivity and to enhance culture yield, thereby increasing the use of targeted therapy. Although this was not our primary objective, it remains an important area for future investigation. Additionally, existing literature supports the prevalence of low culture positivity in critical care settings.  At our tertiary care center, where patients are often referred from other facilities, several factors contribute to the low culture positivity rates observed. Notably, the clinical condition of patients, often severely ill upon arrival, may not align with culture results, leading to negative findings despite the presence of infection. Additionally, in the context of India's healthcare landscape, the lack of a networking database and the prevalent high use of antimicrobials, particularly ‘Watch’ and ‘Reserve’ group of antimicrobials in peripheral centers, can influence the choice and efficacy of antimicrobial therapy at our institution.  To address this issue comprehensively, our study has laid the groundwork for future investigations and stewardship interventions. We recognize the importance of exploring factors contributing to culture negativity, such as the impact of antimicrobial usage patterns and the management of referred patients. Moving forward, we are in the process of developing standard operating procedures (SOPs) and implementing front-end strategies, including antimicrobial restriction protocols. Furthermore, we are planning to introduce stewardship education initiatives, such as 8-hour modules for junior and trainee doctors, aimed at promoting judicious antimicrobial prescribing practices and optimizing therapy based on clinical judgment and available evidence. By leveraging the insights gained from our study, we aim to enhance antimicrobial stewardship efforts and improve patient outcomes in our institution and beyond. |
|  | - While the paper mentions the potential for resistance due to high use of 'Watch' and 'Reserve' antimicrobials, it lacks a detailed discussion on the observed or potential trends in antimicrobial resistance. | While our study primarily focused on antimicrobial usage patterns rather than resistance trends, we understand the importance of a detailed discussion on observed or potential trends in antimicrobial resistance. Notably, the common occurrence of carbapenem resistance underscores the urgent need for carbapenem-sparing strategies to address resistance development effectively.  Although antimicrobial resistance trends were not the primary objective of our study, we have provided detailed antimicrobial resistance patterns in the supplementary files accompanying our manuscript. (Figure S4, S5) The relevant text has been added in the text. Also, current prevalence of resistance in India has been added and compared with our study in discussion section. |
|  | - The paper identifies areas for stewardship interventions but does not provide detailed strategies or frameworks for implementing these interventions (e.g. protocols of use?) | We appreciate the reviewer's observation regarding the identification of areas for antimicrobial stewardship interventions in our study. While we delineated these areas, such as the high use of 'Watch' and 'Reserve' antimicrobials, we acknowledge the need for detailed strategies or frameworks for implementing these interventions. Building upon this recognition, we have already developed comprehensive protocols and guidelines for antimicrobial use, encompassing both empirical and targeted therapy approaches. These protocols are tailored to our institutional context, taking into account local epidemiology, antimicrobial resistance patterns, and clinical practice guidelines.  Moreover, in response to the issue of low culture positivity yield, we have implemented front-end strategies to optimize antimicrobial prescribing practices. Specifically, we are conducting regular clinical rounds involving microbiologists and pharmacologists in collaboration with clinicians to provide audit and feedback on antimicrobial use. Additionally, we recognize the critical role of training programs for healthcare professionals, particularly junior doctors and first-contact clinicians in tertiary care centers, who are often responsible for initiating antibiotics upon patient admission. These periodic training sessions will focus on evidence-based prescribing practices, antimicrobial stewardship principles, and the importance of diagnostic stewardship to enhance culture yield and guide targeted therapy decisions. |
|  | - The conversion of the PICU to a COVID ICU and its impact on antimicrobial use patterns is mentioned but not explored in detail. | It's important to clarify that while our project primarily focused on the PICU, during the COVID-19 pandemic, all ICUs of our institute were converted to COVID ICUs as part of the hospital's response to the crisis. Consequently, the PICU operated at a limited capacity during this period, impacting our data collection.  Given the constraints imposed by the pandemic, including reduced bed availability and operational adjustments, our data collection was limited to the PICU's functioning capacity during this period.  To address this issue, we would like to emphasize that our study focused solely on the antimicrobial usage patterns in non-COVID patients within the PICU setting which was operating with limited bed capacity. To clarify further, our objective was not to assess the antimicrobial use pattern in COVID patients. |
| Reviewer #2 | I agree that your study is very important to establishing a baseline of current antimicrobial use in PICUs and to contribute to the overall data on AM use in order to establish antimicrobial stewardship programs. What was lacking in the current submission was more of a thorough review of your data and why some of your results were significant compared to other similar studies (even in the adult population). What does your data mean basically? As a reader I don't know what the normal antimicrobial use in Indian hospitals is. | Data on antimicrobial consumption in Indian PICUs using standard metrics, such as Days of Therapy (DOT), is either lacking or very scarce. Consequently, it is challenging to ascertain the normal use of antimicrobials. Through this study, we aimed to establish benchmark data using standard metrics in our PICU, which can be compared with data from other facilities. Our observations are significant compared to other studies in adult populations, mainly because data in pediatric populations is very limited. In the absence of pediatric data, we compared our findings with adult studies that used DOT, and we have discussed these significant observations in our publication. The establishment of this benchmark data will be valuable for further studies in the Indian context. |
|  | What do you mean by watch and reserve categories? | Thank you for providing your feedback, and I appreciate the opportunity to address your concerns. In response to your comments, we have taken steps to enhance the clarity and understanding of our study's terminology and methodology.  "Access," "Watch," and "Reserve" categories of antimicrobials are accepted and widely used terminologies as advocated by World Health Organization (WHO). WHO Essential Medicine List also classified antimicrobials into these categories. However, for further clarity, we have included a detailed definition of terms such as "Access," "Watch," and "Reserve" categories of antimicrobials in the supplementary supporting file. We have referenced the WHO's AWaRe classification system, which provides a framework for categorizing antimicrobials and promoting their rational use. This additional information provides readers with a comprehensive understanding of this classification system. Thus, we used this classification system for data analysis which will facilitate future comparisons and contribute to the establishment of baseline data essential for implementing effective antimicrobial stewardship programs in Indian hospitals.  For further information on the AWaRe classification system and its implications for antimicrobial stewardship, readers are encouraged to visit the provided link (<https://www.who.int/publications/i/item/2021-aware-classification>).  Also, definitions of terms such as de-escalation, escalation of antimicrobials have been added in supporting information.  Furthermore, to clarify our methodology, we utilized the Days of Therapy (DOT) metric as a standardized measure for assessing antimicrobial consumption in our study. We recognize that such granular data, particularly in the pediatric ICU setting in Indian hospitals using such metric, is scarce. Without comparable data from other PICUs or benchmarks for normal antimicrobial use, direct comparisons or assessments of significance become challenging. |
|  | A more thorough explanation of some terminology would have been helpful to understand your results: the above terms watch and reserve categories of antimicrobials, de-escalating of antimicrobials are examples. Don't assume the reader will know what this mean. |  |
|  | Some of the tables you included were very busy and hard to read. I would suggest organizing them differently or splitting them into multiple tables if possible. Doing more stats on the data. Were there significant differences in the results. | Thank you for your feedback. While we understand your concern about the readability of some of the tables, we believe that their comprehensive nature was necessary to ensure that all pertinent data was adequately captured and presented.  Our use of descriptive statistics allowed us to effectively summarize and present the data, providing valuable insights into antimicrobial usage without the need for further statistical tests. Therefore, we believe that conducting additional statistical analysis would not significantly enhance the interpretation of our findings and may not be appropriate given the scope and objectives of our study. |
|  | You made some interesting observations and perhaps some conclusions without fully addressing (or including) some things. You noted that longer stays and mortality increased with more antimicrobials? was that because the patients were sicker? you should at least address that in your conclusion and either try to explain it or perhaps say that observation needs to be studied more. | As mentioned in our previous responses, we acknowledge the potential confounding factors, such as the severity of illness, that may influence the relationship between antimicrobial usage and patient outcomes. In this study, we did not record APACHE scores to determine the severity of patients, so correlation of severity was not our primary objective. To prove associations definitively, properly matched studies with multivariate analysis would be necessary. Therefore, we agree that further research is warranted in this area.  We have addressed this observation and emphasized the need for additional research to better understand the complex interplay between antimicrobial usage, patient characteristics, and clinical outcomes in the pediatric ICU setting. By acknowledging this limitation and calling for further study, we aim to contribute to ongoing efforts to improve our understanding of antimicrobial prescribing practices and their impact on patient care.  This study is the first to demonstrate a correlation between prolonged hospital stays and increased mortality rates associated with the use of multiple antimicrobials. We observed a relationship between antimicrobial escalation and higher mortality rates, as well as extended lengths of stay (LOS). Future research is warranted to conduct a detailed analysis of the various factors affecting mortality and LOS. We have added this in our discussion section. |
|  | Another observation was the low culture yield and you suggest that could be a result of previous therapy before they were admitted. Is there a limitation to checking if they had a positive result early on in that previous admission to support this? Could the culture result actually be a true negative? could there be inappropriate treatment just based on not trusting culture results? | While we acknowledge the possibility that previous therapy before admission could influence the culture yield, checking for positive results from previous admissions may indeed provide valuable context. However, there are limitations to this approach. First, obtaining comprehensive medical records from previous admissions, especially in a tertiary care setting where patients may have received treatment at multiple facilities, can be challenging and may not always be feasible. Additionally, even if positive culture results were documented in previous admissions, it may not definitively establish the presence of an ongoing infection at the time of admission to the pediatric ICU.  Furthermore, it's important to consider that low culture yield can also be attributed to various other factors, such as inadequate sample collection, transport, or processing, as well as the presence of non-bacterial infections or non-infectious conditions. Therefore, while previous therapy may contribute to low culture yield, it's essential to interpret negative culture results cautiously and consider the clinical context and other contributing factors.  In some cases, inappropriate treatment decisions based solely on negative culture results without considering the clinical context or trusting the culture results may indeed occur. This underscores the importance of diagnostic stewardship as well as clinical judgement to exercise clinical decisions for clinicians, rather than solely depending on either. |
| **Reply to the Comments made in the manuscript text** | | |
| 1. | limited might be a better word to use here | Accepted and modified. |
| 2. | suggest saying: with the goal or purpose of developing a facility-wide antibiotic policy.... | Accepted and modified. |
| 3. | you don't describe earlier what this group is so instead say 'reduce the overuse of antimicrobials in specific groups' although I think reducing antimicrobials in all groups is important | Accepted and modified. |
| 4. | use a better word here e.g., inappropriate or just say antibiotic overuse | Accepted and modified. |
| 5. | Perhaps explain the difference between antimicrobials and antibiotics since you use them interchangeably in this section | Antibiotic term has been replaced with antimicrobial for uniformity. |
| 6. | Broad spectrum antimicrobials don't cause infections of MDROs but can lead to infections by these organisms | Accepted and modified. |
| 7. | I would argue that they are no more special than any other population of patients. Maybe you mean there are special considerations for paediatric patients? | To clarify, when referring to children as a special population, we recognize that they require unique considerations in healthcare, particularly regarding pharmacokinetics and pharmacodynamics. However, we acknowledge that other populations, such as geriatrics and pregnant individuals, also have specific needs and considerations related to medication management. Therefore, by "special population," we intend to encompass all groups with distinct physiological characteristics that may impact drug therapy, rather than singling out pediatric patients as uniquely special. |
| 8. | Is this an issue in Indian hospitals? (In the context of low culture-yield) | Culture negativity can indeed be a challenge in Indian hospitals, as in healthcare settings worldwide. Several factors contribute to culture negativity, including pre-hospital antimicrobial use, inadequate sample collection, transport, or processing, and the presence of non-bacterial infections or non-infectious conditions. Additionally, variations in laboratory practices and resources may impact culture yield. While culture negativity or inadequate documentation of culture-sensitivity reports is not unique to Indian hospitals, it remains an important consideration in antimicrobial prescribing and diagnostic stewardship efforts, emphasizing the need for comprehensive strategies to optimize culture yield and guide appropriate antimicrobial therapy in clinical practice. |
| 9. | has been poorly documented up until now | Accepted and modified. |
| 10. | why did you only include those who received antibacterial. To understand the overall use of antimicrobials shouldn't you include everyone. By not including all patients you are only getting data on antibacterial use in children who receive antibacterials. | Inappropriate use of antimicrobials is increasingly common, with a high prevalence of carbapenem resistance and a rising trend in colistin resistance. We are facing a critical shortage of effective antimicrobial agents to treat difficult-to-treat bacteria due to this growing resistance. The World Health Organization (WHO) has also emphasized the importance of antibacterial agents, leading to the development of the AWaRe (Access, Watch and Reserve) classification for these agents. Since the inception of our project, our objective has focused exclusively on antibacterials. However, it is essential to extend stewardship efforts to include antifungal and antiviral agents as well.  Focusing on patients who received antibacterial agents allows us to capture detailed information on prescribing practices, including indications for use, duration of therapy, and adherence to antimicrobial stewardship guidelines. This targeted approach enables us to provide meaningful insights into antibacterial utilization trends, identify potential areas for improvement, and inform targeted interventions aimed at optimizing antimicrobial prescribing practices and patient outcomes in the PICU. |
| 11. | Do you reference or explain what these are? and why do these categories matter to the reader? | In response to your comment, we have added clear definitions of the "Watch" and "Reserve" group antimicrobials in the supplementary supporting file. These categories are based on the WHO's AWaRe classification system, which classifies antibiotics according to their importance for human medicine and aims to promote their rational use. For further information, readers can refer to the WHO website, where detailed information on the AWaRe classification system is available (<https://www.who.int/publications/i/item/2021-aware-classification>). |
| 12. | The table is too busy and hard for the reader to know what they are looking at and why. Its obvious you did a lot of analysis but its not clear what is important or significant in the data. Is there data that stands out is important for the reader to know? How does this data compare to other studies in pediatrics? | Thank you for highlighting the importance of key data points in our study. We have noted that cephalosporins were the most commonly prescribed antimicrobials in our pediatric intensive care unit (PICU), which is a significant finding indicating prescribing patterns within our institution. Additionally, we have included data on Days of Therapy (DOT) and average treatment days for individual antimicrobials in the table, providing insights into the duration and frequency of antimicrobial use in our PICU population. This information is crucial for understanding prescribing practices and evaluating the appropriateness of antimicrobial therapy.  Furthermore, we have conducted comparisons of our data with existing literature in the discussion section of our manuscript. By contextualizing our findings and identifying similarities or differences with other studies in pediatrics, we aim to provide a comprehensive analysis of antimicrobial prescribing practices and contribute to the broader understanding of this topic.  We appreciate your input and have ensured that these important data points are highlighted in our study to enhance the clarity and significance of our findings.  Despite the table appearing busy, researchers in this field can easily interpret a comprehensive overview of antimicrobial consumption. |
| 13. | Please explain better what de-escalation of therapy means. Does it mean therapy was stopped? dosage reduced? different antibacterial used? Same with escalation | In response to your comment, we have added clear definitions of "de-escalation" and "escalation" in the supplementary supporting information to provide better understanding. |
| 14. | Difficult for the reader to understand this table. Can it be organized better? What are the columns under the S.N header? it is not intuitive to the reader | Thank you for your feedback. We understand the importance of clarity in presenting the data. The columns under the "S. N." header represent different components related to antimicrobial therapy, including de-escalation, escalation, and continuation. We have simplified it. Legends were added below the table. |
| 15. | what is your criteria for HA MRSA, MDRO etc? Is there a standard surveillance definition you can reference please. How does this compare to other country's rates etc? Its nice to know this but its not useful if there is no comparison. The reader doesn't know if this is high or low. | The criteria for hospital-associated methicillin-resistant Staphylococcus aureus (HA-MRSA), multidrug-resistant organisms (MDROs), and other surveillance definitions are provided in the supplementary supporting information. These definitions typically adhere to established guidelines and standards set forth by reputable healthcare organizations such as the Centers for Disease Control and Prevention (CDC) or the World Health Organization (WHO).  Regarding comparison data, while it would be valuable to contextualize our findings with rates from other countries or institutions, comparative data is often limited or not readily available. However, we acknowledge the importance of benchmarking our rates against established norms or trends reported in the literature to provide a better understanding of the prevalence and significance of HA-MRSA, MDROs, and other antimicrobial-resistant organisms in our setting. |
| 16. | Please explain why it is especially crucial in children? | Explained in the manuscript and justified in previous response (#please check Point 7) |
| 17. | I recommend wording this instead as 'Beta-lactams with or without.......were the most commonly used antibacterials in our study' | Accepted and modified. |
| 18. | Again, it’s not clear to the reader what watch and reserve groups mean and why are they relevant to stewardship | In response to your comment, we have added clear definitions of the "Watch" and "Reserve" group antimicrobials in the supplementary supporting file. These categories are based on the WHO's AWaRe classification system, which classifies antibiotics according to their importance for human medicine and aims to promote their rational use. For further information, readers can refer to the WHO website, where detailed information on the AWaRe classification system is available (<https://www.who.int/publications/i/item/2021-aware-classification>). |
| 19. | Do antimicrobials in the reserve group tend to have longer recommended treatment durations that would explain your observation? Please explain why your results for the reserve group were notable? | Reserve group antimicrobials are last resort drugs. They are definitely prescribed more in critically ill patients admitted in ICUs. However, their use should be limited, and every attempt should be made to de-escalate them. Hence, this observation is meaningful and stewardship strategies regarding reserve group drugs is need of the hour. |
| 20. | how do the authors explain prior antimicrobial use and low microbial yield influencing overuse of antimicrobials?  Do the authors mean low culture positivity rather than low microbiological yield? | Accepted and modified. |
| 21. | is this because the kids were sicker? As a reader I read this statement as the more antimicrobials given the more likely the patients were to have a longer stay or die. Is that correct? | Thank you for pointing that out. You are correct. Our primary objective was to observe antimicrobial usage patterns and their associations with clinical outcomes in the pediatric intensive care unit (PICU) setting. Being intensive care setting, the patient population was critically ill. As such, we did not calculate APACHE scores to determine the severity of illness among patients. However, we acknowledge the importance of considering severity of illness as a potential confounding factor when analyzing the relationship between antimicrobial therapy and clinical outcomes. While we observed a correlation between escalation of therapy and poorer outcomes, such as higher mortality rates and extended PICU stays, further analysis is required to determine causation. It's important to consider that the decision to escalate therapy is often made in response to clinical deterioration or inadequate initial treatment, which may indicate underlying factors contributing to worse outcomes.  Future studies that incorporate APACHE scores or other severity assessment tools may provide valuable insights into this aspect and help elucidate the complex interplay between antimicrobial usage and patient outcomes in the PICU. |
| 22. | could this also be because the infections had resolved and the specimens were truly culture negative? | Indeed, it's possible that some specimens were truly culture negative because the infections had resolved by the time of sampling. However, in the context of our study population being admitted to the pediatric intensive care unit (PICU), it's important to consider that these patients are typically critically ill and often have persistent or unresolved infections contributing to their clinical presentation.  In addition to the possibility of resolved infections, several other factors may contribute to the low culture-positivity yield observed in our study. These factors include pre-hospital antimicrobial use, inadequate sample collection techniques, delays in sample transport or processing, as well as the presence of non-bacterial infections or non-infectious conditions mimicking infectious diseases.  Therefore, while the resolution of infections may contribute to some instances of culture negativity, it's likely that a combination of factors, including the severity of illness and healthcare practices, influences the culture yield in our study population. Further investigation into these factors is warranted to better understand and address the challenges associated with low culture-positivity yield in the PICU setting. |
| 23. | Did you check to see if the patients in your study had positive cultures at the other hospitals if they were previously admitted before receiving them? | In our study, we did not have the tools or a tracking system to systematically check whether patients had positive cultures at other hospitals prior to their admission to our facility, due to unavailability of e-health record system. Unfortunately, in the healthcare landscape of India, there is currently no networked database that allows for the seamless sharing of patient medical records, including previous culture reports, between different healthcare facilities.  Additionally, the high patient load and resource constraints in tertiary care hospitals, such as ours, pose significant challenges to conducting comprehensive retrospective analyses of patients' prior medical histories, including culture results from other institutions.  While accessing previous culture reports from other hospitals would provide valuable insights into the patients' microbiological profiles and antimicrobial susceptibilities, the current limitations in data sharing and resource availability make this endeavour challenging in our setting.  As such, we relied on the culture results obtained during the patients' current admission to our paediatric intensive care unit (PICU) to inform our analysis of antimicrobial usage patterns and clinical outcomes. We acknowledge the potential impact of not having access to previous culture reports on our study findings and encourage further research efforts aimed at addressing these data-sharing challenges in the Indian healthcare system. |

In conclusion, we believe that the revisions made in response to the reviewers' comments have significantly strengthened the quality and clarity of our manuscript. We are confident that the revised version addresses all concerns raised by the reviewers and is now suitable for publication in PLOS One.

Thank you for considering our rebuttal.

Sincerely,

Prof (Dr.) Nitin Rewaram Gaikwad

Professor and Head

Department of Pharmacology

All India Institute of Medical Sciences, Raipur, Chhattisgarh, India

[Nitingaikwad2707@aiimsraipur.edu.in](mailto:Nitingaikwad2707@aiimsraipur.edu.in)
